# Genome-wide CRISPR/Cas9 screen shows that loss of GET4 increases mitochondria-endoplasmic reticulum contact sites and is neuroprotective

**DOI:** 10.1038/s41419-024-06568-y

**Published:** 2024-03-11

**Authors:** Emma L. Wilson, Yizhou Yu, Nuno S. Leal, James A. Woodward, Nikolaos Patikas, Jordan L. Morris, Sarah F. Field, William Plumbly, Vincent Paupe, Suvagata R. Chowdhury, Robin Antrobus, Georgina E. Lindop, Yusuf M. Adia, Samantha H. Y. Loh, Julien Prudent, L. Miguel Martins, Emmanouil Metzakopian

**Affiliations:** 1 UK Dementia Research Institute, University of Cambridge, Clifford Albutt building, Cambridge biomedical campus, Cambridge, CB2 0AH UK; 2 MRC Mitochondrial Biology Unit, University of Cambridge, The Keith Peters building, Cambridge Biomedical Campus, Cambridge, CB2 0XY UK; 3 MRC Toxicology Unit, University of Cambridge, Gleeson Building, Tennis Court Road, Cambridge, CB2 1QR UK; 4 Cambridge Institute for Medical Research, University of Cambridge, The Keith Peters Building, Cambridge Biomedical Campus, Hills Rd, Cambridge, CB2 0XY UK; 5 Cambridge Advanced Imaging Centre, University of Cambridge, Anatomy Building, Downing Site, Cambridge, CB2 3DY UK; 6 bit bio, The Dorothy Hodgkin Building, Babraham Research Campus, Cambridge, CB22 3FH UK

**Keywords:** Mechanisms of disease, Endoplasmic reticulum, Energy metabolism, Molecular neuroscience

## Abstract

Organelles form membrane contact sites between each other, allowing for the transfer of molecules and signals. Mitochondria-endoplasmic reticulum (ER) contact sites (MERCS) are cellular subdomains characterized by close apposition of mitochondria and ER membranes. They have been implicated in many diseases, including neurodegenerative, metabolic, and cardiac diseases. Although MERCS have been extensively studied, much remains to be explored. To uncover novel regulators of MERCS, we conducted a genome-wide, flow cytometry-based screen using an engineered MERCS reporter cell line. We found 410 genes whose downregulation promotes MERCS and 230 genes whose downregulation decreases MERCS. From these, 29 genes were selected from each population for arrayed screening and 25 were validated from the high population and 13 from the low population. GET4 and BAG6 were highlighted as the top 2 genes that upon suppression increased MERCS from both the pooled and arrayed screens, and these were subjected to further investigation. Multiple microscopy analyses confirmed that loss of GET4 or BAG6 increased MERCS. GET4 and BAG6 were also observed to interact with the known MERCS proteins, inositol 1,4,5-trisphosphate receptors (IP3R) and glucose-regulated protein 75 (GRP75). In addition, we found that loss of GET4 increased mitochondrial calcium uptake upon ER-Ca^2+^ release and mitochondrial respiration. Finally, we show that loss of GET4 rescues motor ability, improves lifespan and prevents neurodegeneration in a *Drosophila* model of Alzheimer’s disease (Aβ42Arc). Together, these results suggest that GET4 is involved in decreasing MERCS and that its loss is neuroprotective.

## Introduction

The traditional concept of cellular organelles in eukaryotic cells is that they combine their functions to orchestrate cellular processes but are independent entities compartmentalised in the cytoplasm. However, it is now well established that physical interactions between organelles play fundamental roles in many aspects of cell biology [1]. We understand that organelles form highly complex networks and that their crosstalk is crucial for the normal development and function of cells. Despite being a relatively recent area of study, the field of organelle contact sites has gained increasing attention, and recent research has identified numerous organelle contact sites, such as contacts between the endoplasmic reticulum (ER) and mitochondria.

Mitochondria-ER contact sites (MERCS) are cellular microdomains characterized by a close proximity (approx. 10–80 nm) of the ER and the outer mitochondrial membrane (OMM), without fusion event [2, 3]. They act as signalling ‘hot spots’ and are associated with a large variety of vital cellular functions, including mitochondrial quality control, unfolded protein response, and lipid and calcium (Ca^2+^) homeostasis [2, 4, 5]. For example, Ca^2+^ influx to the mitochondria matrix, through MERCS, is essential for the efficient function of key Ca^2+^ -dependent respiratory enzymes such as isocitrate dehydrogenase, oxoglutarate dehydrogenase and pyruvate dehydrogenase required to produce ATP [6], essential for cell survival [4, 5, 7, 8].

MERCS have a large variety of proteins associated with them, and different tethering complexes, which control the distance between both organelles have been identified [2, 4, 5]. For example, mitofusin 2 (MFN2) an OMM protein regulating mitochondrial fusion, can also be localised to the ER where it regulates MERCS by homodimerisation or heterodimerisation with MFN2 or mitofusin 1, respectively, at the OMM [9]. Other proteins in MERCS include vesicle-associated membrane protein-associated protein B (VAPB) on the ER and protein tyrosine phosphatase-interacting protein 51 (PTPIP51) on the mitochondria. Loss of either VAPB or PTPIP51 can reduce MERCS, disrupt Ca^2+^ signalling [10, 11] and alter MERCS-regulated functions such as autophagy [12, 13] or synaptic activity [14]. In addition, a tetrameric complex composed of inositol 1,4,5-trisphosphate receptors (IP3Rs), glucose-regulated protein 75 (Grp75), voltage-dependent anion-selective channel protein 1 (VDAC1, VDAC2 or VDAC3) and protein deglycase (DJ-1) acts as a functional tether enabling Ca^2+^ transport into the mitochondria [15–17]. Due to their large variety of functions, it comes as no surprise that MERCS are associated with a range of diseases, including metabolic disorders [18], cancer [19, 20] and neurodegenerative diseases including Alzheimer’s disease (AD) and Parkinson’s disease (PD) [4, 5], making them a fascinating area of research.

The gold standard for visualising MERCS is electron microscopy (EM), as it can resolve MERCS on a nanoscale; however, EM cannot easily be conducted on a large scale or in live cells [21]. Split fluorescence protein-based technologies, such as the mVenus-based biomolecular fluorescence complementation (BiFC) system, allows the analysis of MERCS in a high-throughput manner and within living cells [22]. When the mitochondria and ER come into close proximity during MERCS formation, the ER- and mitochondrial-targeted components of the BiFC system refolds, restoring the mVenus fluorescence [22]. Split fluorescence protein-based systems have been used to investigate proteins associated with MERCS [22–24] and separately in large-scale protein‒protein interaction studies [25–27]. Therefore, the simplicity of the fluorescence readout makes the BiFC system useful for both pooled and arrayed functional genetic screens.

Functional genetic screens have improved our understanding of cell biology and human disease mechanisms [28]; however, the power of functional genetic screens has not yet been used to examine pathways that regulate MERCS in human cells. The efficiency of such screens has been refined by clustered regularly interspaced short palindromic repeats (CRISPR)–Cas9 technology, which can induce targeted double-stranded DNA breaks, resulting in the loss of gene function [29, 30]. Specifically, pooled loss-of-function screens using CRISPR/Cas9 technology can be carried out by using large, combined libraries of gRNAs to examine a range of cellular phenotypes and gRNA enrichment. Molecular determinants of diseases and cellular function including viral-host interactions [31], cancer [32] and macrophage integrity [33] have been better defined using CRISPR–Cas9-based loss-of-function screens. Such screens have also been proposed as a tool to investigate new molecular pathways involved in neurodegeneration [4].

To identify novel regulators of MERCS, we combined a pooled CRISPR/Cas9 genome-wide library with a BiFC-based tool to measure MERCS in human cells by flow cytometry. We identified several genes involved in the regulation of contacts between mitochondria and the ER. As the increase in MERCS is proposed to be neuroprotective [34], we focused on the targets that promoted the highest increase in MERCS. Using a series of complementary assays, we show that the loss of GET4 and BAG6 increases MERCS. We found that GET4 and BAG6 can interact with the MERCS tethering proteins IP3R1 and GRP75, respectively. In addition, loss of GET4 increased mitochondrial calcium uptake and concentration ([Ca^2+^]), upon ER-Ca^2+^ release, as well as mitochondrial respiration. Furthermore, loss of *get4*, the *Drosophila* orthologue of GET4, increases MERCS in the adult fly brain. Finally, we show that increasing MERCS by the downregulation of *get4* in a fly model of AD, associated with the overexpression of a toxic form of Aβ (Aβ-Arc), is neuroprotective.

## Results

### A novel split mVenus ER-Mito reporter cell line can detect changes in MERCS

**Fig. 1:**
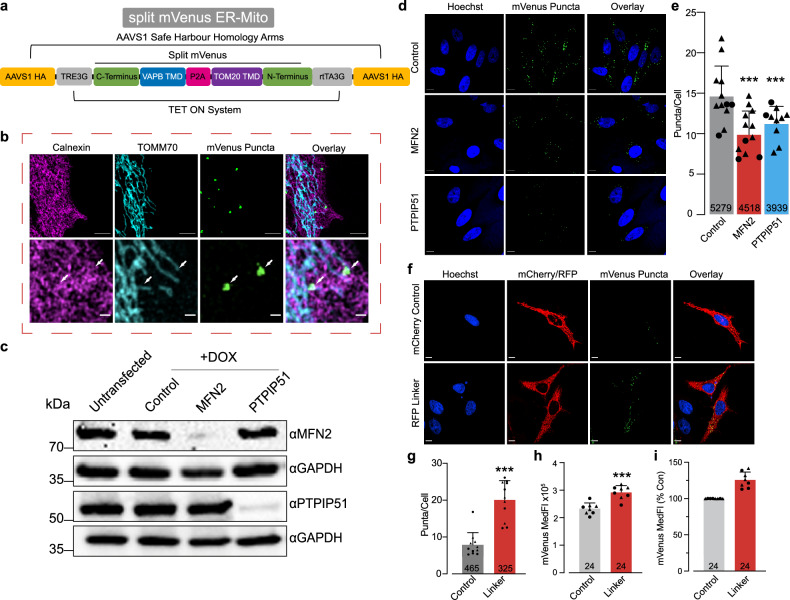
Functional characterisation of an ER-Mito reporter cell line. **a** A scheme of the split mVenus ER-Mito construct used to generate ER-Mito reporter cell lines. The split mVenus system is composed of the transmembrane domains of TOMM20 and VAPB fused to the N- and C-terminal domains of nonfluorescent protein fragments of mVenus, respectively. These constructs were combined within the AAVS1 safe harbour site of the HeLa Cas9 cell line under the control of a promoter regulated by tetracycline. **b** Partial co-localisation of the split mVenus ER-Mito reporter with mitochondria and the ER assessed by immunostaining and N-SIM. Mitochondria were stained with an anti-TOMM70 antibody, and the ER was stained with an anti-Calnexin antibody. Scale bar, 5 μm. **c** Analysis of the downregulation of MFN2 or PTPIP51 in ER-Mito reporter cell lines. ER-Mito reporter cells were transfected with either non-targeting (control), MFN2 or PTPIP51 siRNA for four days and treated with 1 μg/mL doxycycline for 24 h. Cell lysates were analysed by western blotting using the indicated antibodies. **d**, **e** The siRNA-mediated downregulation of MFN2 or PTPIP51 decreases the number of mVenus puncta. ER-Mito reporter cells were transfected with non-targeting (control), MFN2 and PTPIP51 siRNA for 4 days and treated with 1 μg/mL doxycycline for 24 h. Representative images of the mVenus puncta (**d**) detected by spinning disc confocal microscopy in cells co-stained with Hoechst to detect the nuclei and quantified in (**e**). Scale bar 10 μm. Mean ± S.D, number indicates number of cells analysed, *n* = 4 (1× Clone 1, 3× Clone 2), data points = mVenus puncta/cell averaged per coverslip (Con = 12, MFN2 = 12, PTPIP51 = 10), (circles = Clone 1, triangles = Clone 2), data analysed using mixed effects models with significance tests performed using Satterthwaite’s degrees of freedom method with ImerTest. **f**, **g** Overexpression of an artificial RFP linker (composed of N-terminal of mitochondrial protein mAKAP1 and C-terminal, ER localisation sequence of yUBC6 bridged by an RFP (mAKAP1 [34–63]-mRFP-yUBC6) [68]) increases the number of mVenus puncta in ER-Mito reporter cells. Cells were transfected with the RFP Linker or a control-plasmid expressing mito-mCherry (N-terminal of mitochondrial protein mAKAP1 fused to mCherry without the ER localisation sequence) and treated with 1 μg/mL doxycycline for 24 h + 48 h in medium without doxycycline. Representative images (**f**) of mVenus puncta detected by spinning disc confocal microscopy in cells co-stained with Hoechst and quantified in (**g**). Scale bar 10 μm. Mean ± S.D, number indicates number of cells analysed, *n* = 4 (2× Clone 1, 2× Clone 2), data points = mVenus puncta/cell averaged per coverslip (Con = 11, Linker = 11), (circles = Clone 1, triangles = Clone 2), mixed effects models with significance tests performed using Satterthwaite’s degrees of freedom method with ImerTest. **h**, **i** Overexpression of the ER-mito linker increases mVenus fluorescence intensity. ER-Mito reporter cells were transfected with ER-Mito linker (mAKAP1 [34–63]-mRFP-yUBC6) [68] and treated with 1 μg/mL doxycycline for 24 h + 48 h in medium without doxycycline. The MedFI was measured by flow cytometry (**h**) in both clones, and the percentage change in MedFI is shown in (**i**). Mean ± S.D., *n* = 8 (4× Clone 1, 4× Clone 2), data points = mVenus medFI averaged per biological replicate (Con = 8, Linker = 8), (circles = Clone 1, triangles = Clone 2) mixed effects models with significance tests performed using Satterthwaite’s degrees of freedom method with ImerTest.

To gain a comprehensive understanding of genes that regulate MERCS, we used a flow cytometry based, loss-of-function forwards genetic screen employing a CRISPR/Cas9 genome-wide library. To do this we generated a MERCS reporter construct (Fig. 1a) based on a previously published split mVenus system [22] and incorporated it into the AAVS1 safe harbour site of the HeLa-Cas9 cell line under a tetracycline ON promoter, generating two independent clones for screening (see Methods and Supplementary Fig. 1a–c).Throughout this study both clones were treated as independent biological replicates and the combined analysis of both clones is shown in main figures. For completion, the independent analysis of clone 1 and clone 2 alone are shown in Supplementary Figs 7, 8, 9. Next, we determined that the two ER-Mito reporter cell clones used in our screen show efficient Cas9 cutting activity (Supplementary Fig. 1d, e). To determine the levels of mVenus fluorescence that would be suitable for our screen, we performed a time course analysis of doxycycline induction (see Methods and Supplementary Fig. 2a-d). We found that treating ER-Mito reporter cells with doxycycline for 24 h, followed by its removal for an additional 48 h, was optimal for our screen. Using structured illumination microscopy (SIM) to visualise mVenus puncta in the ER-Mito reporter cells, we observed that the mVenus signal colocalised with a subset of mitochondria in close association with the ER, confirming that the mVenus fluorescence can label MERCS (Fig. 1b).

Subsequently, we asked if the ER-Mito reporter cell lines can detect changes in MERCS caused by the suppression of MFN2 or PTPIP51, two well-established tethers whose loss reduces MERCS [9–11, 14, 35]. We found that the siRNA-mediated knockdown of either MFN2 or PTPIP51 decreased their protein levels (Fig. 1c) and reduced the number of mVenus puncta observed by confocal microscopy (Fig. 1d, e, Supplementary Fig. 7a), but not the mVenus median fluorescence intensity (MedFI) measured by flow cytometry (Supplementary Fig. 2e, f). This was further confirmed using PTPIP51 knockout pools, where no decrease in MedFI was found despite the reduction in PTPIP51 protein levels (Supplementary Fig. 2g–i). To determine if the ER-Mito reporter cell lines can detect an increase in MERCS, we expressed a synthetic, RFP tagged ER-mitochondria linker, reported to increase MERCS [36]. Expression of the ER-mitochondria linker resulted in a significant increase in both mVenus puncta (Fig. 1f, g, Supplementary Fig. 7b) and MedFI (Fig. 1h, i, Supplementary Fig. 7c, d) compared to an mCherry control. Together, these data indicate that the ER-Mito cell lines can be used to detect increases in MERCS by microscopy and flow cytometry, however a decrease in MERCS can be only detected by microscopy.

### Identification of BAG6 and GET4 as regulators of MERCS

**Fig. 2:**
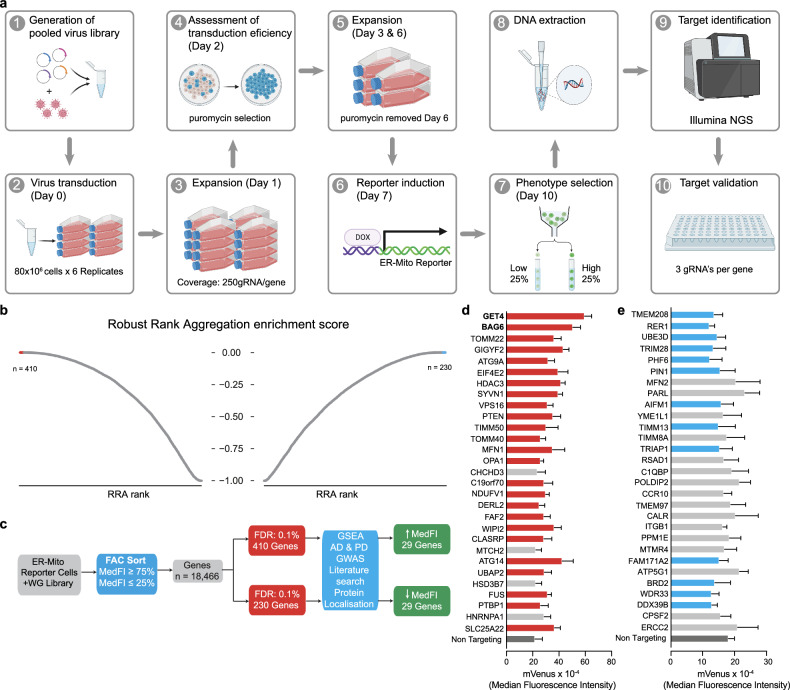
CRISPR‒Cas9 screening identified key genes involved in protein integration into the ER membrane. **a** A scheme showing the key steps used to conduct a pooled genome-wide CRISPR‒Cas9 screen. **b** Enrichment of gRNAs in the populations of cells sorted for either increased or decreased mVenus fluorescence. The gene rank and enrichment score were obtained from the robust rank aggregation method from 6 biological replicates (3× Clone 1 and 3× Clone 2). **c** A scheme showing the process by which the genes were selected for arrayed screening. **d**, **e** Target validation of 29 significantly enriched gRNAs from either the high fluorescence (**d**) or the low fluorescence (**e**) populations, showing that 25 (red) and 13 (blue) guide RNAs significantly (*P* ≤ 0.05) alter mVenus fluorescence. Mean ± S.D, *n* = 4 (2× Clone 1 and 2× Clone 2) mixed effects models with significance tests performed using Satterthwaite’s degrees of freedom method with ImerTest.

To identify new regulators of MERCS, we used our novel MERCS reporter cell line in a pooled CRISPR/Cas9 screen using an unpublished whole genome gRNA library (Supplementary Table 1) as a tool. A pool of gRNA lentivirus was generated and used to transduce 80 million ER-Mito reporter cells (per replicate (*n* = 6)) with a multiplicity of infection of 0.3 and an estimated coverage of 250 gRNA per gene per replicate. The transduced cells were cultured over 10 days, fluorescence-activated cell sorting was conducted on Day 10, and DNA was extracted as shown in Fig. 2a. The high and low MedFI populations were compared to identify 410 genes that positively regulated MERCS and 230 genes that negatively regulated MERCS (FDR = 0.01%) (Supplementary Table 2). This included two known proteins associated with MERCS, MFN2 [9] and TOMM40 [37], as well as neurodegenerative disease-associated genes [38, 39] such as WIPI2, CLASRP, BAG6, SOD1 and FUS which increase MERCS and MTCH2 and PARL which decrease MERCS (Fig. 2b). Gene Ontology (GO)-term analysis identified overrepresentation of specific pathways including autophagy, DNA damage and mitochondria organisation (Supplementary Fig. 3a, b). We next focused on 29 genes from each population for secondary validation. These genes were manually selected based on an assessment of functionalities identified by gene-set enrichment analysis (GSEA), genes implicated in AD and PD GWAS studies, localisation to either mitochondria or ER and their overall function (Fig. 2c). GO-term and functional network analysis using STRING [40] found that the top enriched pathways for the 58 selected genes from both populations consisted of mitochondrial or membrane organisation pathways as well as those involved with protein insertion into membranes (Supplementary Fig. 3c–e). We then performed secondary flow cytometry, CRISPR KO based arrayed validation screen with these 58 genes. We validated an increase in mVenus fluorescence for 25 out of 29 (Fig. 2d) and a decrease in mVenus fluorescence for 13 out of 29 (Fig. 2e). We found that the disruption of either GET4 or BAG6, which form a protein complex in the cytosol that acts to regulate tail-anchored (TA) membrane proteins [41, 42], caused the highest increase in mVenus fluorescence (Fig. 2d). Together, these data demonstrate that we have established a novel CRISPR-Cas9 flow cytometry-based screen from which we have identified potential candidates for the regulation of MERCS.

### Lower expression of GET4 or BAG6 increases MERCS

**Fig. 3:**
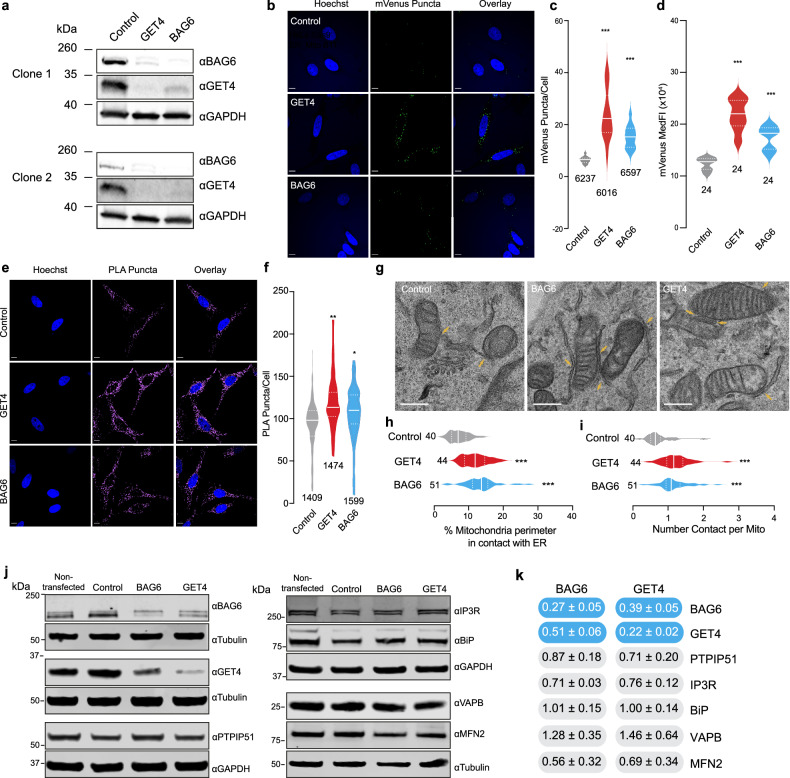
Decreased levels of GET4 or BAG6 increase mitochondria-ER contacts. **a** Analysis of the downregulation of GET4 or BAG6 in ER-Mito reporter cells. ER-Mito reporter cells were transfected with non-targeting (control), GET4 and BAG6 siRNA for 3 days. Cell lysates were analysed by western blotting using the indicated antibodies. **b**–**d** Loss of GET4 or BAG6 results in an increase in mVenus puncta and MedFI. ER-Mito reporter cells were transfected with either non-targeting (control), GET4 or BAG6 siRNA for 7 days and treated with 24 h doxycycline (1 μg/mL) + 48 h normal media before analysis. Representative images of the mVenus puncta (**b**) detected by spinning disc confocal microscopy in cells co-stained with Hoechst to detect the nuclei and quantified in (**c**). Scale bar 10 μm. Median ± quartiles, number indicates number of cells analysed, *n* = 5 (3× Clone 1, 2× Clone 2), data points = mVenus puncta/cell averaged per coverslip (Con = 15, GET4 = 13, BAG6 = 15), (circles = Clone 1, triangles = Clone 2), mixed effects models with significance tests performed using Satterthwaite’s degrees of freedom method with ImerTest. The MedFI was measured using flow cytometry in (**d**). Median ± quartiles, number indicates number of wells analysed, *n* = 8 (4× Clone 1, 4× Clone 2), data points = mVenus medFI averaged per biological replicate (Con = 8, GET4 = 8, BAG6 = 8), mixed effects models with significance tests performed using Satterthwaite’s degrees of freedom method with ImerTest for a combination of the two clones. **e**, **f** Loss of GET4 or BAG6 results in an increase in PLA puncta. ER-Mito cells were transfected with non-targeting (control), GET4 and BAG6 siRNA for 3 days, fixed and stained to examine MERCS via PLA. Representative images of the PLA puncta (**e**) detected by immunofluorescence and spinning disc microscopy in cells co-stained with Hoechst to detect the nuclei and quantified (**f**). Scale bar 10 μm. Median ± quartiles, number indicates number of cells analysed, *n* = 3 (1× Clone 1, 2× Clone 2), data points = mVenus puncta/cell averaged per coverslip (Con = 7, GET4 = 8, BAG6 = 8), analysed using mixed effects models with significant tests performed using Satterthwaite’s degrees of freedom method with ImerTest. **g**–**i** The loss of GET4 and BAG6 results in an increase in the percentage of ER in contact with the mitochondria and the number of contact sites per mitochondria. ER-Mito cells were transfected with non-targeting (control), GET4 and BAG6 siRNA for 3 days, fixed, embedded and MERCS examined by TEM. Representative electron microscopy images of the ER-Mito reporter cells in (**g**) with yellow arrows pointing to contacts between mitochondria and the ER (Scale bar 250 nm). The percentage of mitochondrial perimeter in contact with mitochondria and number of contact sites per mitochondrion quantified in (**h**) and (**i**), respectively. Median ± quartiles, number represents the number of cells analysed, Mitochondria analysed (Con = 727, GET4 = 692 and BAG6 = 735), *n* = 3 (1× Clone 1 and 2× Clone 2). Analysed used mixed effects models with significant tests performed using Satterthwaite’s degrees of freedom method with ImerTest. **j**, **k** Analysis of known regulators of MERCS upon downregulation of GET4 or BAG6. ER-Mito reporter cells were transfected with non-targeting (control), GET4 and BAG6 siRNA for 3 days. Cell lysates were analysed by western blotting (**j**) using the indicated antibodies and quantified using Empiria Studio 3.0 (**k**). Mean ± SEM, *n* = 3, protein of interest expression normalised to loading control. One-way ANOVA, Dunnett’s Multiple Comparison test (BAG6, GET4, PTPIP51, BiP, VAPB or MFN2) or Kruskal-Wallis test followed by Dunn’s Multiple Comparison test for non-normal distributions (IP3R). Blue and grey indicate, respectively statistically significant or not-significant (*P* ≤ 0.05 or *P* > 0.05) alterations in protein expression relative to control siRNA.

We next sought to confirm that the disruption of BAG6 and GET4 altered contacts between mitochondria and the ER. The cytosolic BAG6-UBL4A-GET4 protein complex acts to regulate tail-anchored (TA) membrane proteins [41] and is involved in the quality control of membrane-tethered proteins that are located in the ER [42]. Downregulating either GET4 or BAG6 using siRNAs reduced the levels of both GET4 and BAG6 (Fig. 3a), suggesting that the downregulation of these individual components of the BAG6-UBL4A-GET4 protein complex can promote the degradation of the entire complex.

We next determined the consequences of the siRNA-mediated downregulation of GET4 or BAG6 on MERCS using three independent microscopy approaches to measure MERCS. The knockdown of GET4 or BAG6 in our ER-Mito reporter cell line resulted in a significant increase in both mVenus puncta/cell and mVenus MedFI (Fig. 3b–d, Supplementary Fig. 7e–g). Similarly, proximity ligation assay (PLA), a commonly used method to quantify membrane contact sites [43, 44], also showed a significant increase in GRP75-IP3R [44] puncta upon GET4 or BAG6 knockdown (Fig. 3e, f, Supplementary Figs. 7h & 9). Finally, using transmission electron microscopy (TEM), we found that knockdown of either GET4 or BAG6 increased the percentage of mitochondrial perimeter in contact with the ER and the number of MERCS per mitochondrion (Fig. 3g–i, Supplementary Fig. 7i, j). To try and identify the mechanism of action responsible for the increase in MERCS, we measured the protein levels of known MERCS tethers following BAG6 and GET4 knockdowns. However, no statistically significant changes in protein expression were observed for PTPIP51, VAPB, IP3R, and MFN2 (Fig. 3j, k).

To confirm that the increase in MERCS was directly associated with the loss of GET4 or BAG6 and not due to gross changes in either the ER or mitochondria, we examined the morphology of these organelles and the protein expression of ER stress marker BIP. We did not observe any changes in BIP protein levels (Fig. 3j, k) or gross qualitative changes in ER morphology upon suppression of GET4 or BAG6 (Supplementary Fig. 4a, b). We next examined mitochondrial morphology and architecture by confocal microscopy using MitoMAPR [45] algorithm and by TEM examining the aspect ratio of mitochondria, and found no significant changes in cells depleted for GET4 or BAG6 compared to control (Supplementary Fig. 4c–h, Supplementary Fig. 7k–n). Together, these results confirm that loss of GET4 and BAG6 leads to an increase of MERCS, independently of organelle morphologies.

### BAG6 and GET4 interact with the two MERCS-associated proteins IP3R and GRP75

**Fig. 4:**
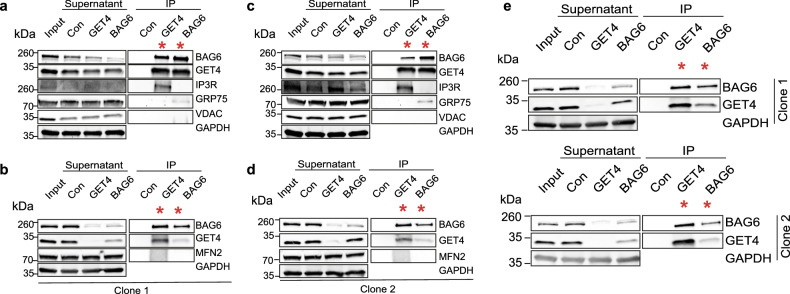
GET4 and BAG6 interact with the mitochondrial ER contact site proteins IP3R and GRP75, respectively. **a**, **b** GET4 and BAG6 interact with IP3R and GRP75, respectively. ER-Mito reporter cell lysates prepared from either clone 1 (**a**, **b**) or clone 2 (**c**, **d**) were incubated with Dynabeads^TM^ and either anti-GET4 or anti-BAG6 antibodies. Cell lysates were analysed with the indicated antibodies. **e** IP-mass spectrometry samples for GET4 and BAG6 detection. ER-Mito reporter cell lysates were incubated with Dynabeads^TM^ and either anti-GET4 or anti-BAG6 antibodies. These were analysed by western blotting, probed with the indicated antibodies and sent for mass spectrometry analysis.

The protein complex containing BAG6 and GET4 acts to sort proteins that fail to properly insert into the ER membrane [42, 46]; however, little is known about a possible role for this complex at MERCS. We therefore asked if GET4 and BAG6 can interact with any key MERCS proteins using GET4- and BAG6-specific antibodies to pull-down GET4/BAG6 complexes by coimmunoprecipitation (Co-IP). We found that the canonical MERCS protein IP3R1 Co-IPs with GET4 and GRP75 Co-IPs with BAG6; however, we failed to detect enrichment for either VDAC1 or MFN2 in either GET4 or BAG6 Co-IPs (Fig. 4a–d).

**Fig. 5:**
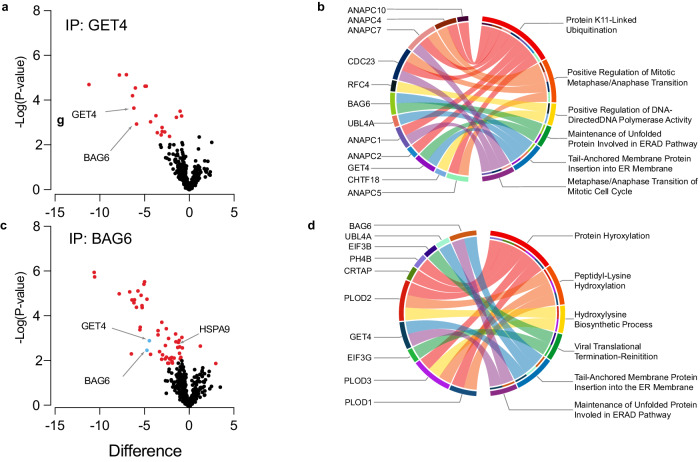
Network analysis of GET4 and BAG6 interactors. **a** Volcano plot showing statistically significant GET4 interactors (red). Control IP samples were compared to GET4 IP samples (Control – GET4) to identify proteins that interact with GET4. GET4 and BAG6 are highlighted. *N* = 4 (2× Clone 1 and 2× Clone 2), pairwise t-tests were performed with a 5% FDR. **b** Chord plots representing the STRING analysis of GET4 interactors and their top 6 biological processes. **c** Volcano plot showing statistically significant BAG6 interactors (red). Control IP samples were compared to GET4 IPs samples (Control – BAG6) to identify proteins that interact with BAG6. GET4, BAG6 and GRP75 are highlighted. *N* = 4 (2× Clone 1 and 2× Clone 2), pairwise *t*-tests were performed with a 5% FDR. **d** Chord plots representing the STRING analysis of BAG6 interactors and their top 6 biological processes.

Next, we used proteomics to identify further GET4 or BAG6 interactors in an unbiased manner. We first confirmed that GET4 and BAG6 were detected from both BAG6 and GET4 Co-IP’s which were subjected to mass spectrometry (Fig. 4e). We identified 19 proteins coimmunoprecipitating with GET4 and 53 proteins interacting with BAG6 (Supplementary Tables 5, 6 and Fig. 5a, c). Among the proteins enriched in GET4 Co-IPs were UBL-containing protein 4 A (UBL4A), HSPA1A and RPS27A, which have roles in the insertion of proteins into the ER membrane. UBL4A is also a common interactor between GET4 and BAG6 (Fig. 5b and Supplementary Table 5). Many GET4 and BAG6 interactors are associated with the ERAD pathway and insertion of proteins into the ER membrane (Fig. 5b, d). In support of our Co-IP analysis of known MERCS components, GRP75 (HASA9) was also enriched in our proteomic analysis of BAG6 Co-IP, confirming the interaction between the GRP75 and BAG6 (Fig. 4a, b). However, IP3R was not detected in our proteomics analysis. Finally, we examined the overlap between GET4 or BAG6 interactors and proteins identified at MERCS by different proteomic studies [47] and found 15 BAG6 and 3 GET4 interactors to be associated with MERCS (Supplementary Fig. 5a–c and Supplementary Tables 4, 5). HSPA8 was identified as an interactor of GET4 and BAG6 and was also present in MERCS [47]. Together, these data establish that GET4 or BAG6 interacts with known components of MERCS.

### Loss of GET4 increases mitochondrial calcium uptake and shifts bioenergetics towards mitochondrial ATP production

**Fig. 6:**
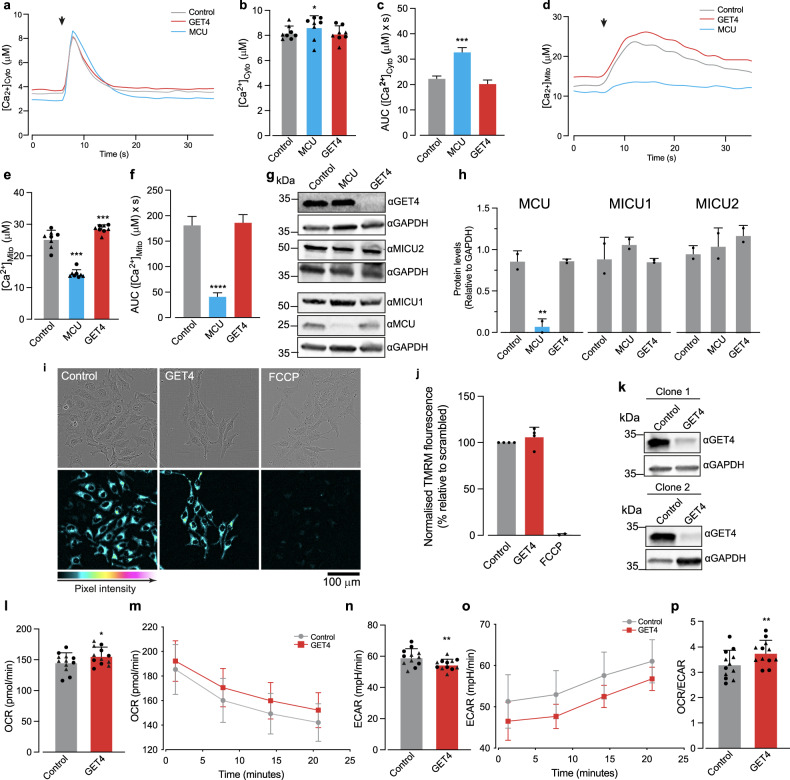
Loss of GET4 increases mitochondrial calcium flux and ATP-linked respiration. **a**–**f** Loss of GET4 increases mitochondrial [Ca^2+^]. ER-Mito cells were transfected with non-targeting (control), GET4 and MCU siRNA for 3 days and analysed using cytosolic and mitochondrial targeted aequorin probes to measure the cytosolic (**a**–**c**) and mitochondrial (**d**–**f**) [Ca^2+^]. Curves showing the cytosolic (**a**) and mitochondrial (**d**) [Ca^2+^] over the first 35 sec with max peak for either cytosolic or mitochondrial [Ca^2+^] shown in (**b**, **e**). Histamine addition at 10 s (arrow). Mean ± S.D., *n* = 8 (4× Clone 1, 4× Clone 2), data points = max peak [Ca^2+^] averaged per plate, Clone 1 (circles) and Clone 2 (triangles), analysed using mixed effects models with significant tests performed using Satterthwaite’s degrees of freedom method with ImerTest. The Area under the Curve (AUC) from baseline (average of values from first 6 s) shown in (**c**, **f**). Mean ± S.E.M., *n* = 8 (4× Clone 1, 4× Clone 2), analysed using One Way ANOVA with Bennetts significance test. **g**, **h** The loss of GET4 does not alter the expression of the mitochondrial calcium uniporter complex proteins. ER-Mito reporter cells were transfected with non-targeting (control), MCU and GET4 siRNA for 3 days. Cell lysates were analysed by western blotting (**g**) using the indicated antibodies MCU, MICU1 and MICU2 and protein levels quantified by densitometry in (**h**), Mean ± S.D (*n* = 2) (1× Clone and 1× Clone 2), One-way ANOVA, Dunnett’s Multiple Comparison test. **i**, **j** knockdown of GET4 does not alter Δψm in cultured ER-Mito reporter cells. ER-Mito reporter cells were transfected with non-targeting (control), GET4 siRNA for 3 days or pre-treated with FCCP for 15 mins. Representative images (**i**) were visualised using a five-tone heat map, and quantitative analysis (**j**) of TMRM fluorescence were performed. Mean ± S.D *n* = 4, one sample *t* test. FCCP is included for reference only, no statistics were conducted. **k** Western blot analysis of the downregulation of GET4 in ER-Mito reporter cells. Cell lysates were analysed by western blotting using the indicated antibodies. ER-Mito reporter cells were transfected with non-targeting (control) or GET4 siRNA for 3 days. Cell lysates were analysed by western blotting using the indicated antibodies. **l**–**p** The loss of GET4 increases basal respiration. ER-Mito reporter cells were transfected with non-targeting (control) and GET4 siRNA for 3 days, and the OCR and ECAR were determined via a seahorse mitochondrial stress assay. Change in the OCR (**l**, **m**), ECAR (**n**, **o**) and OCR/ECAR ratio (**p**) assessed over 20 min. Time points 14 and 21 were combined in (**m**, **o**, **p**) where measurements were least variable and analysed. Mean ± S.D., data is a combination of Clone 1 (circles) and Clone 2 (triangles), *n* = 6 (3× Clone 1 and 3× Clone 2), data points = average of OCR. ECRA or OCR:ECAR ratio averaged per plate for each clone. Data analysed, mixed effects models with significance tests performed using Satterthwaite’s degrees of freedom method with ImerTest.

MERCS have intrinsically been linked to Ca^2+^ homeostasis and ATP production [4, 7, 8], with the loss of MERCs being associated to a decrease of mitochondrial uptake upon ER-Ca^2+^ release [11, 48], which can impact mitochondrial bioenergetics [49]. As we have shown that GET4 can interact with IP3R and that GET4 loss led to increased MERCs, we examined the impact of the loss of GET4 on Ca^2+^ transfer from the ER to mitochondria. We measured intracellular Ca^2+^ concentrations using aequorin probes, that bind coelenterazine and Ca^2+^ to produce luminescence relative to [Ca^2+^] [42]. We depleted either GET4 or the mitochondrial Ca^2+^ uniporter (MCU), a key component of the mitochondrial calcium uptake machinery, and measured the concentration of either cytosolic or mitochondrial Ca^2+^ after treatment with 100 µM histamine to induce ER Ca^2+^ release [50]. Upon histamine stimulation, we observed a small but significant increase in cytosolic max peak of [Ca^2+^] and area under the curve (AUC) with loss of MCU (Fig. 6a–c, Supplementary Fig. 8a–c), which was accompanied by a significant decrease in peak mitochondrial [Ca^2+^] and AUC (Fig. 6d–f and Supplementary Fig. 8d–f). However, following depletion of GET4 and histamine stimulation, while we found no significant change in the cytosolic [Ca^2+^] following the depletion of GET4 (Fig. 6a–c), we observed a small but significant increase in mitochondrial max peak of [Ca^2+^], suggesting an upregulation of Ca^2+^ transfer from the ER to mitochondria (Fig. 6d–f, Supplementary 8d–f). To exclude the possibility that the increased effect observed was associated with upregulation of the different mitochondrial calcium uniporter complex components, we examined their protein levels in GET4 depleted cells. We found no changes in the expression of either MCU, or its main associated regulators MICU1 and MICU2 in cells with suppressed GET4 expression (Fig. 6g, h).

To rule-out that the increased uptake in mitochondrial Ca^2+^ was not due to alterations in mitochondrial membrane potential (Δψm), we used TMRM to measure Δψm following the siRNA-mediated downregulation of BAG6 and GET4. This analysis showed that silencing either BAG6 or GET4 did not cause significant changes in Δψm (Fig. 6i, j).

Ca^2+^ plays an important role in regulating mitochondrial metabolism by stimulating the activity of several mitochondrial TCA cycle enzymes, controlling mitochondrial respiration and energy production [51]. To determine whether the increase in MERCS and mitochondrial Ca^2+^ with the loss of GET4 (Fig. 6k) could be associated with an increase in basal respiration, we measured the oxygen consumption rate (OCR) and extracellular acidification rate (ECAR), reflecting glycolytic activity, using a seahorse XFe96 analyser. We found that the depletion of GET4 led to an increase in the OCR (Fig. 6l, m, Supplementary Fig. 8g), decrease in ECAR (Fig. 6n, o, Supplementary Fig. 8h) and increase in OCR/ECAR ratio compared to the control (Fig. 6p, Supplementary Fig. 8i). Together, these data suggest that by increasing MERCS, the loss of GET4 results in an increase in mitochondrial Ca^2+^ flux, and an increase in mitochondrial respiration.

### Suppressing *get4* is neuroprotective in a *Drosophila* model of Alzheimer’s disease

**Fig. 7:**
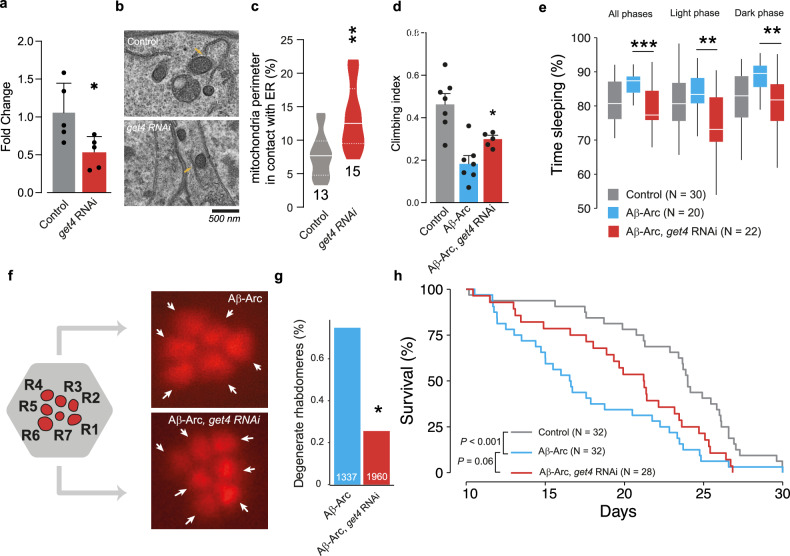
Increasing MERCS by knocking down *get4* alleviates Alzheimer’s disease-related phenotypes in a fruit fly model. **a** RNAi-mediated suppression of *get4* using a neuronal driver reduced the mRNA levels of *get4* in fly heads (means ± SDs; asterisks, Student’s *t* test). **b**, **c** RNAi-mediated suppression of *get4* using a neuronal driver increases MERCS in the adult fly brain. Representative electron microscopy images of the ER-Mito reporter cells in (**b**) with yellow arrows pointing to contacts between mitochondria and the ER. Percentage of mitochondrial surface in contact with the ER (**c**) quantified in (median and interquartile range; asterisks, Student’s *t* test). **d** *get4* RNAi reduces the motor defects caused by Aβ42Arc expression (means ± SDs; asterisks, Student’s *t* test). **e** *get4* RNAi rescued the sleep defects caused by Aβ42Arc expression (means ± SDs; asterisks, Student’s *t* test). Sleep was recorded between Days 10 and 15 posteclosion. **f**, **g** *get4* RNAi reduces the degeneration of photoreceptors in Aβ42Arc-expressing flies (chi-square test, 95% confidence interval). Representative images, together with an illustration of the typical layout of the visible photoreceptors (red, R1–R7) at the surface of the adult *Drosophila* ommatidium (grey hexagon, white arrows indicate individual photoreceptors) in (**f**) and quantification in (**g**). **h** Get4 RNAi extends the lifespan of Aβ42Arc-expressing flies (Wilcoxon rank sum test, with Benjamini‒Hochberg correction). Flies were aged 10 days (**a**–**e**, **h**) or 15 days (**f**, **g**) post-eclosion. Genotypes: elavGal4; +; + (control), elavGal4; +; UASAβ42Arc (Aβ-Arc) and elavGal4; UAS get4RNAi; UAS Aβ42Arc (Aβ-Arc, get4RNAi).

MERCS are dysregulated in AD as well as other neurodegenerative diseases [4, 5]. Familial AD is associated with the toxic aggregation of amyloid-β (Aβ) and can be modelled in *Drosophila melanogaster* by neuronal expression of a disease-associated Aβ (1–42) with an Arctic mutation (Glu22Gly) (Aβ-Arc). Importantly, increasing MERCS in Aβ42-Arc-expressing *Drosophila* can rescue AD-linked phenotypes [34]. We therefore hypothesised that the suppression of *get4* could rescue the AD-linked phenotypes of flies expressing Aβ42-Arc by increasing MERCS in vivo. First, we measured the transcript levels of *get4* in the heads of flies and confirmed the successful RNAi-dependent suppression of *get4* using a neuronal driver (Fig. 7a). Ultrastructural analysis of fly brains showed that the neuronal suppression of *get4* resulted in a significant increase in the surface of mitochondria in contact with the ER (Fig. 7b, c). Next, we tested whether the targeted neuronal suppression of *get4* by RNAi could be neuroprotective in flies expressing Aβ42-Arc by assessing phenotypes associated with Aβ toxicity. We found that the neuronal suppression of *get4* in flies expressing Aβ42-Arc improved motor performance (Fig. 7d), decreased excessive sleep (Fig. 7e), reduced rhabdomeres degeneration (Fig. 7f, g) and improved survival (Fig. 7h). From this, we conclude that the neuronal suppression of *get4* is neuroprotective in this fly model of AD, and this likely occurs through the increase in MERCS.

## Discussion

MERCS have previously been implicated in a range of diseases, including metabolic disorders [18], cancer [19, 20] and neurodegenerative diseases [4, 5] however, how they are modulated, and their disease mechanism is not well understood. Therefore, we employed a split mVenus system [22] to conduct a genome-wide CRISPR/Cas9 screen and identified novel genetic regulators of MERCS. By combining this system with a genome-wide CRISPR/Cas9 loss-of-function library, we identified 410 genes whose loss-of-function increased MERCS and 230 that decreased MERCS. The top two candidates whose loss increased MERCS, GET4 and BAG6, were shown to interact with key MERCS proteins IP3R1 and GRP75, respectively, and GET4 was found to be neuroprotective in a *Drosophila* model of AD.

The BiFC system has been previously used in large-scale protein–protein interaction studies [25–27, 52] however, this is its first use in a flow cytometry-based whole-genome screen investigating modulators of MERCS in an unbiased manner. A potential limitation of our screen is that it was performed in a polyploid immortalised cell line, so the true degree of ER-Mito reporter incorporation into the genome is difficult to determine. To address this issue, we used two independent ER-Mito reporter clones that generated comparable results. Induced pluripotent stem cell-derived cortical neurons have recently been used to conduct both pooled and arrayed screens [53], and it would have been informative to determine whether a screen using cortical neurons can confirm our results.

Our ER-Mito reporter system can detect alterations in MERCS when contact sites are genetically manipulated (Fig. 1). However, it lacks sensitivity for the detection of the decrease in MERCS by flow cytometry (Supplementary Fig. 2e, f). The main limitation of split fluorescence complementation-based systems, such as the BiFC system, is that the formation of the fluorescent protein process is energetically favourable and therefore lacks reversibility [54]. Once the fluorescent protein is stably folded, it is less prone to “split” in response to stimuli that reduce MERCS. This makes it less sensitive for the detection of screen hits that reduce MERCS.

GET4 and BAG6 were the top hits in the pooled screen, and their role in regulating MERCS was further validated in a secondary arrayed screen. The downregulation of either GET4 or BAG6 increased MERCS (Fig. 3) without altering the structure of either the mitochondria or the ER (Supplementary Fig. 4). GET4 and BAG6 form a complex with UBL4A to sort TA membrane proteins for either degradation or insertion into the ER [41, 42, 55]. Mutations in GET4 can decrease the levels of BAG6 in patient fibroblasts, disrupting the transmembrane domain recognition complex pathway [56] and the insertion of TA membrane proteins into organelles. We found that the downregulation of GET4 reduces the levels of BAG6 and vice versa (Fig. 3a); Therefore, it is possible that lowering the levels of a single component of the BAG6-UBL4A-GET4 protein complex is sufficient to reduce the levels of the entire protein complex. Furthermore, mutations in the yeast GET complex alter the insertion of pex15, a TA protein, into the ER and compromise peroxisome formation [57, 58]. Therefore, loss-of-function of GET4 or BAG6 may be able to alter the ratio of other MERCS-related TA proteins, such as VAPB, PTPIP51 or MFN2. We conducted western blotting to examine changes in MERCS tethering proteins MFN2, PTPP51, VAPB and IP3R but saw no significant change in their cellular expression (Fig. 3j, k), however their expression in MAM and other subcellular compartments was not examined. Investigating this may allude to more subtle changes in TA protein expression within subcellular compartments. BAG6 is also known to promote the degradation of MFN2 [59] and regulates mitophagy [60]; Although how GET4 and BAG6 regulate MERCS is not known it is possible that reducing the activity of the BAG6-UBL4A-GET4 protein complex increases the levels of MERCS tethers regulating MERCS indirectly. In summary, the mechanism by which GET4 or BAG6 regulate the formation of MERCS is unclear but we reason that our study sets the stage for further studies to understand how the aberrant sorting of TA proteins alters organellar contacts.

MERCS regulate mitochondrial morphology as they act in mitochondrial fission and fusion [61, 62]. It has been shown that increasing MERCS in *Drosophila* by forced expression of an artificial tether decreases mitochondrial length [34]. We however found no overt morphological changes in either mitochondria or the ER upon loss of GET4, suggesting that the role MERCS play in mitochondrial morphology is independent of GET4 or BAG6. However, GET4 and BAG6 may impact a subset of MERCS with alternative functions [2] such as calcium buffering (Fig. 6). It is also possible that the loss of GET4 or BAG6 compromises mitochondrial dynamics of transport, but we did not measure these features in our study.

We found that GET4 interacts with IP3R, the main Ca^2+^ channel in the ER, and observed an interaction between BAG6 and GRP75, a chaperone protein that forms a complex with DJ-1, IP3R and VDAC1 to regulate Ca^2+^ homeostasis at MERCS. The BAG6-GRP75 interaction was confirmed by IP-mass spectrometry; however, the GET4-IP3R interaction was not. This is likely to reflect technical difficulties with the detection of IP3R by mass spectrometry in our settings. We found no interactions between GET4 and GRP75 or BAG6 and IP3R, despite previous reports of interaction between GET4 and BAG6 and separately between IP3R and GRP75. This may indicate that they may a diverse range of downstream pathways independent of their main functions within TA protein insertion or calcium regulation. A common interactor between GET4 and BAG6, which has been found in MERCS [47], is HSP70 (HSPA8). HSP70 is a chaperone protein that regulates the folding of core proteins associated with cellular survival and homeostasis [63] and is also associated with guided entry into tail-anchored proteins [64]. BAG6 is a cochaperone for HSP70 and can negatively regulate HSP70 protein folding ability [65]. Both BAG6 and HSP70 have been associated with a range of neurodegenerative diseases. HSP70 upregulation was observed in mouse models of PD with α-synuclein overexpression and has been shown to inhibit assembly of α-synuclein fibrils [66] and prevent parkin misfolding [67]. Together, these data may suggest additional roles for BAG6 in the aetiology of neurodegenerative diseases.

Alterations in MERCS have previously been shown to impact Ca^2+^ flux from the ER to mitochondria, with the expression of an RFP linker increasing the transfer of Ca^2+^ from the ER to the mitochondria via IP3R, VDAC and MCU [68]. The loss of either PTPIP51 or VAPB can also result in decreased Ca^2+^ transfer [10, 11]. Alterations in Ca^2+^ can impact mitochondrial function because some enzymes involved in the citric acid cycle in the mitochondrial matrix are regulated by Ca^2+51^. From our studies, we observed a small increase in mitochondrial [Ca^2+^] and respiration with the loss of GET4, suggesting that this increase in mitochondrial [Ca^2+^] maybe due to an increase in MERCS. We are, however, using a wild-type system where respiration and Ca^2+^ homeostasis is optimal, hence leaving only a small range of changes in function to be detected.

MERCS have been associated with various neurodegenerative diseases, including PD, AD and ALS [4, 5]. More specifically, the expression of an artificial ER-Mito tether (RFP linker) increases MERCS and rescues climbing ability and survival in a *Drosophila* AD model (Aβ42Arc) [34]. Our data showed that knockdown of GET4 in Aβ42Arc *Drosophila* increases MERCS and rescues motor ability and survival and is neuroprotective. This suggests that GET4’s role in MERCS regulation is conserved in *Drosophila* and that increasing MERCS via loss of GET4 is beneficial in a *Drosophila* model of AD. Interestingly, however, other data have suggested that a decrease in MERCS, owing to the loss of PDZD8, is able to rescue AD mutant phenotypes [69]. The literature surrounding this has been conflicting; exposure of oligomeric Aβ in primary hippocampal neurons and AD patient fibroblasts shows an increase MERCS and altered Ca^2+^ homoeostasis [70, 71]. However, in AD, rat hippocampal neurons showed a decrease in MERCS, correlating with a reduction in lipid metabolism and specific alterations in mitochondrial lipids [72]. This raises the hypothesis that both increasing and decreasing MERCS can be protective and might be dependent on specific pathways and proteins.

We were unable to conduct these experiments with loss of BAG6, as the *bag6* RNAi line had a phenotype associated with the misintegration of the RNAi construct [73]; however, it would have been informative to conduct these experiments.

The involvement of GET4 in AD and neurodegeneration is limited, with few roles associated with it. However, the GET4 gene has altered poly(A) site usage as a result of WT tau [74], a protein known to make fibrils in AD, that can bind disease-causing fragments of TDP-43^219^ and contribute to ALS [75]. Furthermore, GET4 and BAG6 can interact with FOBOX7, a PD-causing gene [76], suggesting that GET4 can be linked to neurodegeneration, but its role in MERCS modulation is unknown. Future work is needed to determine if the expression levels of either *GET4* or *BAG6*, as well as potential DNA variants in their coding sequences that alter their biological activity are linked to neurodegeneration in humans.

In summary, we have utilised a split mVenus system to generate an ER-Mito reporter cell line and designed a novel whole genome screen to identify modulators of MERCS. The cell line and data generated from this screen are of great benefit to the wider scientific community, as the screening hits can open avenues of new research into MERCS modulation. There are also opportunities to progress this further with additional screening examining the effect of specific insults or drug treatments. This work has highlighted novel genes involved in MERCS regulation and provided a variety of potential modulators for further research, allowing us to gain a better understanding of MERCS biology.

## Methods

### Cloning and plasmids

#### Generation of the inducible ER-Mito reporter

A split mVenus system was a gift from the Skehel lab [22]. From this, we generated a single ER-Mito reporter construct: Each of the two fragments (TOMM20-mVenus and VAPB -mVenus) of the split mVenus system were amplified via PCR using specific Gibson assembly primers that also incorporated a self-cleaving T2A site between the two fragments. The backbone is based on a doxycycline NGN2 inducible construct [77]. The backbone consisted of a 3^rd^ generation TET-ON system, allowing temporal control via doxycycline, a LoxP flanked removable mCherry-Puromycin for clonal selection and AAVS1 safe harbour homology arms for incorporation into the AAVS1 safe harbour locus. NGN2 was removed via restriction enzyme digest, and the split mVenus constructs with homology arms were combined using Gibson assembly mix (NEB, E2611) for 1 h(h) at 50 °C. These were transformed into NEB 10-beta competent E. coli, colonies picked, and Sanger sequenced to confirm insertion of the full-length reporter construct.

#### Arrayed cloning of gRNAs

The top 3 gRNAs of each gene were selected from the pooled screen, and the sequences were acquired. For each gRNA, a forwards and reverse oligo was designed, including specific BBS1 complementary overhang sequences. Forwards (caccg- gRNA-gt) Reverse (ttaaac-gRNA-c). The forwards and reverse primers were annealed and then ligated into the BBS1 cut U6-sgRNAv2-ccdb-BFP-PURO plasmid. These were then transformed into NEB 10-beta competent *E. coli* (NEB, C3019I) using 20 µL of competent cells per well in a 96-well clear round bottom deep well plate (Axygen, P-2ML-SQ-C) by applying heat shock (1 min at 42 °C) followed by incubation for 1 h at 37 °C with 1 mL of SOC media. Twenty microlitres of the transformation mix was transferred to 24 deep-well culture plates (Axygen, 12537837) with 5 mL media and incubated overnight for 24 h at 30 °C. Cells were pelleted, and DNA was extracted in 96-well plates. DNA was quantified using Quant-iT™ PicoGreen™ (Thermo, P7589) and normalised to 10 ng/µL ready for use in virus generation.

#### Cell culture

HeLa cells were maintained at 37 °C with 5% CO_2_ in a tissue culture incubator in minimum essential medium (MEM) + Glutamax (Gibco, 42360-032) substituted with 10% fetal bovine serum (FBS) (Sigma, F9665) and 1× nonessential amino acids (NEAA). A total of 1.8 µg of Cas9-Blast PiggyBac and 200 ng of transposase (pBac) were electroporated into the HeLa cell line using the Amaxa SF Cell line Nucleofector kit (Lonza, V4XC-2012). Cas9-positive cells were selected using 10 µg/mL blasticidin S HCl (blast) (Gibco, A11139-03) over 10 days to generate a pool of HeLa Cas9-blast cell lines. A gRNA targeting the AAVS1 safe harbour (GGG GCC ACU AGG GAC AGG AU), 2 µg donor ER-Mito mCherry Puromycin reporter construct and 20 µg of Alt-R™ S.p. HiFi Cas9 Nuclease (IDT, 1081060) was nucleofected using the Amaxa SF Cell line Nucleofector Kit (Lonza, VCA-1003) and placed at 32 °C for 48 h. ER-Mito mCherry-positive cells were selected using 5 µg/µL puro. Cells were maintained in Puromycin (Gibco, A1113803). and blast to generate a Cas9-blast mCherry-puro population. The ER-Mito mCherry-Puro pool was then treated with Cre recombinase to remove the Lox P-flanked mCherry-puro. Clones were picked, and their DNA was extracted before storage. The clones were genotyped using two primer pairs (Supplementary Fig. 1a) that can detect a loss of the Lox-P-flanked mCherry-puro region. The PCR products of 3 clones along with genomic DNA from control cells (con) that were not treated with Cre and still contained the mCherry-Pruo construct were compared. With primer set 1, there was a shift in the band, while primer set 2 showed a loss of a PCR product, showing that mCherry-puro was removed (Supplementary Fig. 1a, b). Flow cytometry was used to select mVenus-positive and mCherry-negative cells for expansion and further testing (Supplementary Fig. 1c). Clones 1 and 2 were taken forwards, but Clone 3 was not. To determine the levels of mVenus fluorescence that would be suitable for our screen, we performed a time course analysis of doxycycline induction (Supplementary Fig. 2a–d). We found that treating ER-Mito reporter cells with doxycycline for 24 h, followed by its removal for an additional 48 h, resulted in levels of mVenus fluorescence that were lower than the maximum observed levels at 24 h, indicating that the fluorescence at 48 h was not saturated.

#### Virus generation

Flasks (pooled gRNA library) and plates (arrayed gRNA library) were coated with 25 µg/mL PDL (Gibco, A38904-01) for 3 h or overnight, washed with PBS and Hek cells plated at 80–90% confluence. Transfection was conducted using Lipofectamine LTX (Invitrogen, 15338) reagent as per these ratios for one well of a 96-well plate. Reaction A (20 µl Optimem, 19.12 ng psPAX2, 12.5 ng pMD2. G, 0.1 µL PLUS reagent) and 25 ng gRNA plasmid combined with Reaction B (5 µL optimem and 0.3 µL Lipofectamine LTX). Plates or flasks were spun down (300 × *g* for 5 min), and the medium was aliquoted (arrayed gRNA Library) or centrifuged at 7000 g at 4 °C overnight, and the pellet was resuspended in PBS.

#### Next generation sequencing

Pooled next-generation sequencing (NGS) screening was conducted as described in the results and depicted in Fig. 2a. Once FAC sorted via their MedFI, the DNA was extracted from each population. Cells were lysed for 4 h at 55 °C in DNA lysis buffer (50 mM Tris pH 8, 100 mM NaCl, 10 mM EDTA, 1% SDS and 0.5 mg/ml proteinase K (Thermo, EO0491)). After 4 h, DNA was precipitated using an equal volume of isopropanol and resuspended in TE. The gDNA was quantified using quBit, and the whole sample was used in multiple PCRs to enrich gRNA cassettes (2 µg DNA, 1.5 µL 10 µM primers, 25 µL Q5 master mix (NEB, M0492S)).

**Table:** 

Pi7_PLVPBnewSeq	GTCTCGTGGGCTCGGAGATGTGTATAAGAGACAGACTCGGTGCCACTTTTTCAA
Pi5_PLVPBnobarcode	TCGTCGGCAGCGTCAGATGTGTATAAGAGACAGTCTTGTGGAAAGGACGAAACA

The PCRs were pooled, bead purified, and the product was used in a second PCR to add index adaptors (25 µL KAPA HiFi Hot start ready mix, 1 µL 10 µM primers i5 and i7 combinations). The product was quantified using the NEBNext Library Quant Kit for Illumina (NEB, E7630S) diluted to 4 nM and sequenced by NextSeq (Illumina) using the manufacturer’s instructions for NextSeq 500/550 High Output Kit 75 Cycles (Illumina, 20024906).

### Whole genome CRISPR screen analysis

#### gRNA counting

21 nucleotides (nt) long sequencing reads were exported to FASTQ format from bcl format using bcl2fastq v2.2.0. The reads were mapped to a CRISPR library containing 91,536 gRNA sequences with a length of 20 nt that targeted coding regions of the human genome. Each read was mapped to a gRNA sequence by using a sliding window method that generated 20-mers and an exact match lookup on the CRISPR library gRNA table. Reads without an exact match were discarded. After mapping the gRNAs, we counted gRNA occurrences and merged individual sample count vectors into a unified count table.

#### Quality control

The proportion of gRNAs without an exact match was 21–23% in the generated samples. Samples were inspected for proper gRNA infection and coverage. All samples had sufficient coverage and sequencing depth (Supplementary Fig. 6).

#### Gene enrichment analysis

Enriched genes were identified using the MAGeCK package using Robust Rank Aggregation (RRA) mode [78]. Our experimental conditions included 6 samples that were split into three conditions during sorting (high, low and unsorted). To this end, we ran three different analyses with MAGeCK using the samples in paired mode. The non-targeting gRNAs were used as a negative control in the analysis using the --control-sgRNA option. To investigate enriched genes, we considered the High vs. Low analysis.

#### Arrayed screening

The arrayed screen was conducted in the same way as the pooled screen. On Day 0, 18,500 ER-Mito reporter cells were transduced with supernatant from the arrayed gRNA library. Each gene has three associated gRNAs that were pooled upon transduction. Cells were split on Day 1 to maintain 90% confluency, and BFP expression was confirmed on Day 2. Cells were selected with 5 μg/μL puromycin from Days 2–7. Doxycycline (1 μg/mL) was added on Day 7 for 24 h, and on Day 10, the fluorescence intensity was assessed by flow cytometry.

#### Cell transfections

ER-Mito cells were cultured until 70% confluent and transfected with Lipofetamine LTX (Invitrogen, 15338) in 24-well plates. For each well, Reaction A (120 µL) Optimem (Gibco, 31985-062), 0.6 µL Lipofectamine plus and 500 ng DNA (RFP Linker and Control) was combined with Reaction B (30 µL Optimem and 1.8 µL Lipofectamine LTX) for 20 min at room temperature (RT). The transfection mix was then added to the cells for 24 h. These were scaled up or down depending on the plate surface area.

ER-Mito cells were reverse transfected with 20 nM siRNA using Lipofectamine RNAi Max (13778-075) according to the manufacturer’s instructions. The medium was changed after 1 day, and the cells were harvested, fixed (see immunofluorescence) and stained on Days 3-4. For knockdowns from 72–96 h, 20 nM siRNA was retransfected on Day 4 to maintain the knockdown for up to 7 days.

#### siRNAs

The siRNAs used in this study are listed in Supplementary Table 7.

#### Coimmunoprecipitation

The Dynabeads^TM^ Antibody Coupling Kit (Invitrogen, 14311D) was used following the manufacturer’s instructions, with any deviations highlighted. Five micrograms of antibody per 1 mg of beads was cleaned up using Bio-Spin 30 Tris columns (Bio-Rad, 7326231). For mass spectrometry, 4 mg of beads were used per IP. Purified antibody was incubated with Dynabeads at 37 °C for 24 h at 1500 rpm on a heated shaker. Protein lysates were harvested in protein lysis buffer (20 mM Tris pH 7.5, 100 mM NaCl, 1% Triton, 10% glycerol, 10 nM MgCl_2_ and 1× Halt TM protease inhibitor cocktail, (Thermo, 1861278)) for 20 min, centrifuged at 20,000 x *g* and quantified using a Pierce^TM^ BCA protein assay kit (Thermo, 23225). Protein (3–5 mg) was incubated with antibody-coupled beads for 24 h at 4 °C. Beads were washed 4 times with protein lysis buffer and eluted in 20–50 µL 1× loaded buffer (5 min 95 °C). Input and supernatants were collected, and 20 µL of each ran alongside the IP sample via WB.

#### Mass spectrometry

Data were acquired on a Q Exactive Plus (Thermo Scientific) with an EASY spray source coupled to an RSLC3000 with mobile phases A (0.1% formic acid) and B (80% acetonitrile, 0.1% formic acid). Peptides were fractionated using a 50 cm C18 column (PepMap, Thermo Scientific) using a gradient rising from 3–40% solvent B over 10 min. The spray voltage was 1.3 kV, and MS/MS spectra were acquired using a DIA strategy with an isolation window of 10 m/z scanning from 350 to 900 m/z. Data were processed in DIA-NN[1] 1.8.1 [79] using library generation with the UP000005640 database (downloaded 23/09/20) and a list of common contaminants (MaxQuant). The resulting proteinGroups were processed in Perseus 1.6.2.1. Intensity values were log2 transformed, and the data were filtered to require a minimum of three valid values across all samples. Missing values were replaced with normally distributed values, and pairwise t tests were performed with a 5% false discovery rate (FDR).

#### Western blotting

Protein lysates were harvested in RIPA buffer (Sigma, R0278) and 1× Halt TM protease inhibitor cocktail (Thermo, 1861278) for 15 min, centrifuged at 20,000 x *g* and quantified using a Pierce^TM^ BCA protein assay kit (Thermo, 23225). Samples were mixed with 4× Laemli loading buffer (Bio-Rad, 1610747) and loaded into 12-well PROTEAN TGX Precast Gel (Bio-Rad, 446-9035). Typically, 30 µg of protein was loaded to check knockdown and up to 100 µg for Co-IP. These were run using the Bio-Rad system and transferred using either the Trans-Blot Turbo Rapid semi dry system or the Mini Tans-Blot Electrophoretic Transfer cell (Bio-Rad, 1703930). Transfers were completed using 0.2 µm PVDF single application transfer packs (Bio-Rad, 1704157) with either the high (10 min), mixed (7 min) molecular weight settings or using 0.45 μm nitrocellulose transfer membranes (Scientific Laboratory Supplies, 10600003). Blocking was conducted using 5% milk (VWR, 84615.0500) in PBS for 1 h at RT. Primary antibody incubation was performed in 2% milk in PBS Tween (0.05%) (PBS-T). All primary antibodies were incubated overnight and then washed in PBS-T (3 × 10 min) until secondary antibodies were added in 2% milk PBS-T for 1 h at RT. The membranes were washed in PBS-T (3 × 10 min) and developed using SuperSignal Chemiluminescence substrate (Thermo, 34578) on Bio-Rad ChemiDoc^TM^ MP or Invitrogen iBrightCL1000. Densitometry analysis was conducted using ImageJ (Fig. 6) or Empiria Studio 3.0 (Fig. 3).

#### Antibodies

The primary antibodies used in this study are listed in Supplementary Table 8.

#### Proximity ligation assay

ER-Mito cells were plated, fixed, quenched and permeabilized using the immunofluorescence protocol. PLA was conducted using Duolink In Situ PLA Probe kits, Duolink In Situ detection reagents far red (Sigma, DUO92013-100RXN) and Duolink in Situ Wash buffer (Sigma, DUO82049) according to the manufacturer’s instructions. Blocking was conducted using 1x blocking buffer for 1 h at 37 °C. Primary antibodies were added and incubated at 4 °C in antibody dilutant overnight in a humidified chamber. The next day, the cells were washed with Duolink Buffer A (2 × 5 min washes) and incubated with Duolink anti-mouse plus and anti-rabbit minus probes for 1 h at 37 °C. The coverslips were further washed in buffer A (2 × 5 min) and incubated with 1x ligase for 30 min at 37 °C. After two 5 min washes, the coverslips were incubated with 1x polymerase for 100 min at 37 °C. After this last incubation, the coverslips were washed in Duolink buffer B (2 × 10 min), in 0.01x buffer B for 1 min, then in ddH2O and incubated with Hoechst if needed. Coverslips were mounted using Dako Mounting media (S3023).

#### Immunofluorescence

Immunofluorescence was performed as previously described in Nagashima et al. [80]. Cells were plated on coverslips at a density of 20,000-25,000 cells per well in a 24-well plate. The next day, the cells were fixed with warmed paraformaldehyde (PFA) in PBS (pH 7.4) for 15 min at 37 °C, treated with 50 mM NH_4_Cl_2_ for 10 min at RT to quench autofluorescence and incubated with 0.1% Triton for 10 min at RT. Blocking was conducted using 10% FBS-PBS for 20 min at RT. Primary antibodies were incubated in humidity boxes overnight at 4 °C in 5% FBS-PBS. The next day, the coverslips were washed three times in 5% FBS-PBS and incubated with secondary antibodies for 1 h at RT in 5% FBS-PBS. Cells were washed three times in 1x PBS, incubated with Hoechst if needed, and then rinsed in ddH2O before mounting on slides using Dako Mounting media (S3023) or Prolong gold Diamond (Thermo, P36965) for spinning disk confocal or structured illumination microscopy (SIM), respectively.

#### Electron microscopy

On Day 0, ER-Mito cells were siRNA transfected, as per the above siRNA transfection protocol. On Day 2, the cells were replated into cell culture imaging dishes (Ibidi, 81156). On Day 3, samples were fixed in situ in Ibidi dishes in fixative (2% glutaraldehyde/2% formaldehyde in 0.05 M sodium cacodylate buffer pH 7.4 containing 2 mM calcium chloride) overnight at 4 °C. After washing 5x with 0.05 M sodium cacodylate buffer pH 7.4, samples were osmicated (1% osmium tetroxide, 1.5% potassium ferricyanide, 0.05 M sodium cacodylate buffer pH 7.4) for 3 days at 4 °C. After washing 5× in deionised water (DIW), samples were treated with 0.1% (w/v) thiocarbohydrazide/DIW for 20 min at RT in the dark. After washing 5 times in DIW, samples were osmicated a second time for 1 h at RT (2% osmium tetroxide/DIW). After washing 5 times in DIW, samples were block-stained with uranyl acetate (2% uranyl acetate in 0.05 M maleate buffer pH 5.5) for 3 days at 4 °C. Samples were washed 5 times in DIW and then dehydrated in a graded series of ethanol (50%/70%/95%/100%/100% dry) and 100% dry acetonitrile, 3 times in each for at least 5 min. Samples were infiltrated with a 50/50 mixture of 100% dry acetonitrile/Quetol resin mix (without BDMA) overnight, followed by 3 days in 100% Quetol (without BDMA). Then, the sample was infiltrated for 5 days in 100% Quetol resin with BDMA, exchanging the resin each day. The Quetol resin mixture was 12 g Quetol 651, 15.7 g NSA (nonenyl succinic anhydride), 5.7 g MNA (methyl nadic anhydride) and 0.5 g BDMA (benzyldimethylamine; all from TAAB). Samples were placed in embedding moulds and cured at 60 °C for 3 days.

Sample blocks were cut from the Ibidi dishes using a hacksaw. After sectioning through the plastic coverslip using a glass knife, thin sections through the cell monolayer were cut with a diamond knife using an ultramicrotome (Leica Ultracut E), and the sections were placed on bare copper TEM grids. Samples were imaged in a Tecnai G2 TEM (FEI/Thermo Fisher Scientific) run at 200 keV using a 20 µm objective aperture to improve contrast. Images were acquired using an ORCA HR high-resolution CCD camera (Advanced Microscopy Techniques Corp, Danvers USA).

For the electron microscopy in flies, 5-day old adult fly brains were fixed overnight in 0.1 M sodium cacodylate buffer (pH = 7.4), containing 2% paraformaldehyde, 2.5% glutaraldehyde and 0.1% Tween-20 at RT for 2 h and at 4 °C overnight, with constant rotation. After fixation, the samples were stained with 5% aqueous uranyl acetate overnight at RT; then, they were dehydrated via a series of ethanol washes and embedded in TAAB epoxy resin (TAAB Laboratories Equipment Ltd., Aldermaston, UK). Semi-thin sections were stained with toluidine blue, and areas of the sections were selected for ultramicrotomy. Ultrathin sections were stained with lead citrate and imaged using a MegaView 3 digital camera and iTEM software (Olympus Soft Imaging Solutions GmbH, Münster, Germany) with a Jeol 100-CXII electron microscope (Jeol UK Ltd., Welwyn Garden City, UK).

#### Confocal microscopy

Confocal images were acquired using a Nikon Eclipse TiE inverted microscope with appropriate lasers coupled to an Andor Dragonfly 500 spinning disk system equipped with a Zyla 4.2 PLUS sCMOS camera (Andor). Images were acquired using Fusion software. Seven stacks of 0.2 μm each were acquired using the 60x or 100x objective (NA 1.4). Images from the same experiment, acquired under the same conditions of laser intensity and exposure time, were then compiled using “max projection” in FIJI software.

#### Structured illumination microscopy (SIM)

Super resolution images (Fig. 1b) were obtained using a Nikon SIM microscope using an SR Apo TIRF 100× 1.49 N.A. oil objective and a DU897 Ixon camera (Andor). Eleven *z*-stacks of 0.2 μm were acquired from each region of interest and then computationally reconstructed using the slice reconstruction system from NIS-Elements software (Nikon).

#### mVenus or PLA puncta analysis

Cells underwent the above immunofluorescence and/or PLA protocol and image acquired as stated on an Andor Dragonfly confocal microscope with a 60× objective. Ten to fifteen z-stacks were taken per coverslip, 2/3 coverslips were analysed per condition, and this was conducted for a minimum of three biological replicates using a mix of clone 1 and clone 2 (please see figure legends for specific details regarding number of biological replicates, clones, coverslips and cells). Channels were split, and the number of nuclei and puncta were assessed as described in this ref. [43]. The puncta/cell were then determined per field of view.

#### MERCS analysis

Cells underwent the above electron microscopy fixation and embedding process before imaging. 10–15 cells were imaged per condition and three biological replicates were conducted.

MERCS were analysed in imageJ to analyse Mitochondrial perimeter, MERCS (the length of the ER ≤ 50 nm from the mitochondria), number of mitochondria and number of MERCS per mitochondria. MERCS length by mitochondrial perimeter were quantified using the freehand line tool in ImageJ (NIH, USA) and percentage of surface area of mitochondria in contact with the ER was calculated by dividing MERCS length by mitochondrial perimeter and multiplied by 100.

For flies, 25 random pictures were taken where mitochondria were found. A MERCS was considered when the distance between ER and mitochondria was ≤30 nm. MERCS length by mitochondrial perimeter were quantified using the freehand line tool in ImageJ (NIH, USA) and % of surface area of mitochondria in contact with the ER was calculated by dividing MERCS length by mitochondrial perimeter and multiplied by 100.

#### Mitochondrial morphology analysis

ER-Mito cells underwent the above immunofluorescence protocol and were imaged on an Andor Dragonfly confocal microscope with a 100× objective. Ten to fifteen z-stacks were taken per coverslip, 2/3 coverslips were analysed per condition. Two to three region of interests were taken per image and underwent batch MitoMAPR [45] analysis in ImageJ. The parameters measured from the ROIs were as follows: number of objects (number of mitochondrial particles with no junction points), number of networks (number of objects that contain at least 1 junction point), number of junction points (a junction point is defined as a single pixel in the skeletonised image that has more than three neighbouring pixels, thus being a node from which a branch arises), number of junction points per network and mitochondrial length.

Cells underwent the above electron microscopy fixation and embedding process before imaging. Mitochondrial morphology was analysed by examining the aspect ratio, the ratio between the height and width of a mitochondrion, using TEM electrographs. The height and width was determined using the line tool in image J and the height/width. This was conducted on 12 cells from each experimental condition analysing a total of over 100 mitochondria. please see figure legends for specific details regarding number of clones, coverslips, mitochondria, and cells.

#### Calcium quantification

On Day 0, cells were siRNA transfected, as per the above siRNA transfection protocol. On Day 2, the cells were replated into white solid 96-well plates (Thermo, 136101) at a seeding density of 20,000 cells per well and transduced with adeno-associated virus containing either cytosolic or mitochondrial aequorin. Twenty-four hours after transduction, the cells were washed with 3 times BSS buffer (120 mM NaCl, 5.4 mM KCl, 0.8 mM MgCl_2_, 6 mM NaHCO_3_ 5.6 mM D-glucose, 2 mM CaCl_2_, 25 mM HEPES, pH 7.3) and incubated for 90 min with 5 µM coelenterazine (Thermo, C2944). Cells were then washed an additional three times with BSS buffer and read on a CLARIOstar Plus (BMG LabTech) plate reader. Histamine (100 µM) was added at 10 s, while 10 µM digitonin and calcium chloride were added at 45 s. The mitochondrial and cytosolic [Ca2+] were calculated using the method presented in Bonora et al. [50].

#### Microscopy-based assessment of mitochondrial membrane potential

ER-Mito cells were transfected with siRNA constructs according to the previously outlined protocol. Forty-eight hours after transfection the cells were replated into 96 well plates at a seeding density of 10,000 cells per well. Twenty-four hours after plating cells were treated with 40 nM tetramethylrhodamine methyl ester perchlorate (TMRM, Thermo, T668) for 30 min at 37 °C. IncuCyte S3 (Live-Cell Analysis System, Essen BioScience) was used for fluorescence microscopy analysis of TMRM uptake to provide a qualitative readout for Δψm. FCCP corresponds to un-transfected cells pre-treated with an uncoupler (10 μM FCCP, Abcam, ab120081 for 15 mins). TMRM fluorescence was quantified using Incucyte base analysis software (Sartorius) and the total integrated intensity was calculated and normalised to confluency.

#### Genetics and *Drosophila* strains

Fly stocks and crosses were maintained on standard cornmeal agar media at 25 °C. The strains used were elavGAL4, UAS_Aβ42ARC and w; +; 86 F (described in Yu et al. [81]) and UAS get4RNAi (Vienna *Drosophila* RNAi Center, VDRC_P{KK102820}VIE-260B). All experiments on adult flies were performed using males.

#### Locomotor assays and lifespan analysis

Locomotor and lifespan were assessed as previously described in Yu et al. [81] and in Popovic et al. [82]. Adult male flies were aged to 10 days old post eclosion and individually loaded in glass tubes (80 mm × 5 mm × 3 mm) containing the same food used for rearing. The flies were grown and analysed in a light/dark 12 h/12 h cycle at 25 °C. The total number of recorded midline crossings per minute was recorded using the Drosophila Activity Monitoring System (Trikinetics, Waltham, MA), and the data were analysed using Rethomics [83]. The analysis started at the first ZT0 to allow acclimation. Sleep was calculated for the first 5 days and the data of flies that died were discarded. Sleep is defined as 5 min of inactivity. The data for lifespan analysis are presented as Kaplan–Meier survival distributions. We recorded the entire lifespan of the flies from 10 days post-eclosion until their death.

#### Pseudopupil analysis

Measurements of pseudopupils as a marker of neurodegeneration was performed as previously described [81]. The heads of 5-day-old flies were directly fixed on standard microscope slides using quick-dry transparent nail varnish. A Zeiss Axioplan 2 microscope equipped with a ×63 oil immersion objective was used to visualise the ommatidia. Around 5 flies per condition were examined, to obtain a total number of around 200 ommatidia or ~1400 rhabdomeres. The percent abnormal rhabdomeres was calculated as the number of degenerate rhabdomeres over the total number of rhabdomeres: (A × 1 + B × 2 + C × 3)/N, where A = number of ommatidia with 6 rhabdomeres, B = number of ommatidia with 5 rhabdomeres, C = number of ommatidia with 4 rhabdomeres and N = total number of ommatidia counted. Statistical significance was determined using two-tailed chi-squared test.

#### Climbing assay

Climbing assays were performed using a counter-current apparatus equipped with six chambers as previously described [82]. A total of 10–15 male flies were placed into the first chamber, tapped to the bottom, and then allowed 20 s to climb a distance of 10 cm. The flies that successfully climbed 10 cm or beyond within 20 s were then transferred to a new chamber, and both sets of flies were given another opportunity to climb the 10-cm distance. This procedure was repeated a total of five times. After five trials, the number of flies in each chamber was counted to calculate the climbing index. A video demonstrating this technique can be found at https://youtu.be/vmR6s_WAXgc. The climbing index was measured using a weighted average approach with the following formula:

$$\frac{[(0\times {n}_{0})+(1\times {n}_{1})+(2\times {n}_{2})(3\times {n}_{3})(4\times {n}_{4})(5\times {n}_{5})]}{[5\times SUM({n}_{0}:{n}_{5})]}$$

n_0_ corresponds to the number of flies that failed the first trial, and n_1_-n_5_ are the numbers of flies that successfully passed each successive trial.

#### RNA extraction and quantitative real-time PCR with reverse transcription

This assay was performed as previously described [82]. Total RNA was extracted from 30 fly heads per sample using TRIzol (Ambion), and quantified by spectrophotometric analysis (Nanodrop, Thermo Scientific). Quantitative real-time PCR with reverse transcription (RT–qPCR) was performed with a real-time cycler (Applied Biosystems 7500, Fast Real-Time PCR Systems) using the SensiFAST SYBR Lo-ROX One-Step Kit (Bioline). Fold change was calculated using the comparative Ct method. For RT–qPCR we measured the coefficient of variation (CV) of the technical replicates and excluded from statistical analysis any samples with CV > 3%. Gene-specific primers were designed with FlyPrimerBank (DRSC FlyPrimerBank (flyrnai.org)), and subsequently obtained from Sigma:

*Get4*: forward, 5′-TACGGCGCAGAAACGCTATC-3′,

reverse 5′-GCTTTCCTGTTCCTTGGCAATAA-3′.

*rp49* was used as the housekeeping gene:

forward, 5′-TGTCCTTCCAGCTTCAAGATGACCATC-3′,

reverse 5′-CTTGGGCTTGCGCCATTTGTG-3′.

#### Statistics

Data in main figures are shown as the mean ± SD and uses the combination of both clones 1 and 2 for analysis, treating each clone as biological replicates. The clones were compared in Supplemental Figs. 7, 8 for any clonal variation. No values were excluded from the analyses.

For when clones were combined and quantified, significance tests were performed using linear mixed effects models and Satterthwaite’s degrees of freedom method with the ImerTest [84] R package. The formula for the linear mixed effect models was ${Response\; variable} \sim {Condition}+{Clone\; group}+(1{|random\_effect\_}1)+(1{|Randome\_effect\_}2)$, where the *response variable* is the assayed phenotype (e.g., MERCS, [Ca2+], OCR or mitochondrial morphology), *condition* is the genetic manipulation (e.g., BAG6 or GET4 siRNA), *clone group* refers to one of the two clones with successful integration (described in Supplementary Fig. 1), *random effects* refers to either *replica* (biological replicate), *coverslip or well* (technical replicates) or *plate*. Significance is indicated as *P* < 0.1, * *P* < 0.05, ** *P* < 0.01, *** *P* < 0.001 and ns for *P* ≥ 0.05. The full code and raw data are available in our GitHub repository.

For multiple comparisons involving two groups where the clones were not combined, Prism (V9 GraphPad) was used to analyse data using Shapiro-Wilk normality test and Two-way ANOVA with Dunnett’s multiple comparison test or One-way ANOVA, Dunnett’s Multiple Comparison test or Kruskal–Wallis test followed by Dunn’s Multiple Comparison test for non-normal distributions., * *P* < 0.05, ** *P* < 0.01, *** *P* < 0.001, **** *P* < 0.0001 and ns for *P* ≥ 0.05.

For comparisons involving only 2 groups (TMRM analysis) a one sample unpaired t-test was used * *P* < 0.05, ** *P* < 0.01, *** *P* < 0.001, **** *P* < 0.0001 and ns for *P* ≥ 0.05.

Proteomics data were processed in Perseus 1.6.2.1. Intensity values were log2 transformed, and the data were filtered to require a minimum of 3 valid values across all samples. Missing values were replaced with normally distributed values, and pairwise t tests were performed with a 5% FDR.

### Supplementary information

Supplementary Table 1

Supplementary Table 2

Supplementary Table 3

Supplementary Table 4

Supplementary Table 5

Supplementary Table 6

Supplementary Table 7

Supplementary Table 8

Supplementary Information

Full Western Blot Scans

AJ Stats Checklist

Author Change Approval

## Data Availability

Source date files, including raw numerical data, descriptive statistics, normality tests and statistical analysis used in the manuscript, are available in our GitHub repository at https://m1gus.github.io/MitoER-CRISPR-Screen/. The proteomics data were deposited to the ProteomeXchange Consortium with the dataset identifier PXD042710. All other data are available upon reasonable request.
