# Supplementary material for: Genome-wide CRISPR/Cas9 screen shows that loss of GET4 increases mitochondria-endoplasmic reticulum contact sites and is neuroprotective
Source: Cell Death Dis. 2024 Mar 11;15(3):203. doi: 10.1038/s41419-024-06568-y (PMC10928201; doi:10.1038/s41419-024-06568-y)
Supplement: Supplementary file 9 — Supplementary Information [file 41419_2024_6568_MOESM9_ESM.pdf]

Supplementary Information

Genome-wide CRISPR/Cas9 screen shows that loss of GET4 increases mitochondria-endoplasmic reticulum contact sites and is neuroprotective

Willson et al.

Supplementary Figures and Legends

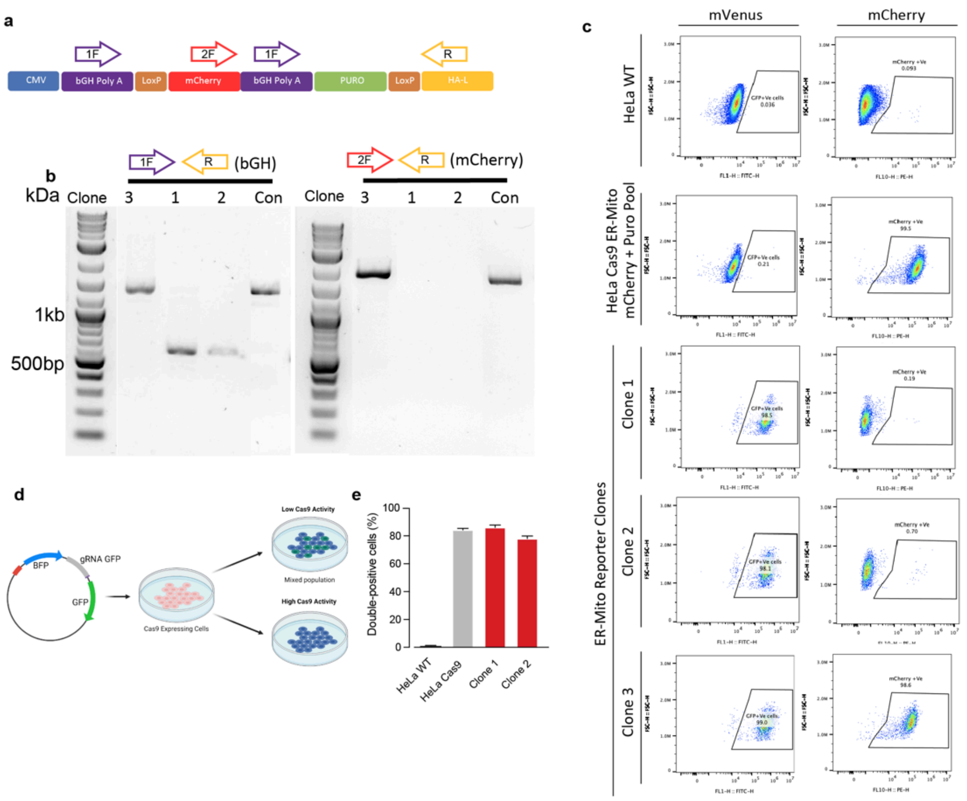

Supplementary Figure 1: HeLa cells show Cas9 activity and stable genomic integration of the ER-Mito reporter construct.

(**a**) A schematic diagram representing two primer pairs (1F and R or 2F and R) used to detect loss of the Lox-P-flanked mCherry Puro region inserted in the genome of ER-Mito reporter cell clones. PCRs with the 1F and R or 2F and R primer pairs are used to detect the loss of the mCherry Puro region caused by the Cre-mediated recombination of that region, which is flanked by LoxP sites. (**b**) ER-Mito cell clones 1 and 2 successfully integrate into the genome of HeLa Cas9 cells. Genomic DNA from three Cre-treated ER-Mito clones was extracted and analysed by PCR using the aforementioned primer pairs highlighted in **(a)**. (**c**) ER-Mito Clones 1 and 2 show robust mVenus expression and loss of mCherry expression. HeLa, ER-Mito mCherry Puro pool and three independent ER-Mito reporter clones were treated with 1 µg/mL doxycycline for 24 h, and mVenus and mCherry expression was analysed by flow cytometry. (**d**) A schematic diagram of the BFP-GFP cutting assay used to assess Cas9 activity. The DNA construct codes for BFP, GFP and a gRNA targeting GFP. When Cas9 is active, the GFP gRNA forms a complex with Cas9. Next, through complementary base pairing, the Cas9-gRNA complex cuts the GFP coding sequence, preventing the expression of GFP. Cas9 expression is therefore monitored by the suppression of GFP fluorescence in cells that are fluorescent for BFP. If there is inefficient Cas9 cutting, then only a proportion of GFP is silenced, and both BFP and GFP will be detected. (**e**) ER-Mito clone 1 and clone 2 show high levels of Cas9 cutting activity. HeLa, HeLa Cas9 pool and ER-Mito reporter cell clones were transduced

with the BFP-GFP gRNA construct, and the percentage of BFP-only cells was analysed by flow cytometry 4 days post transduction.

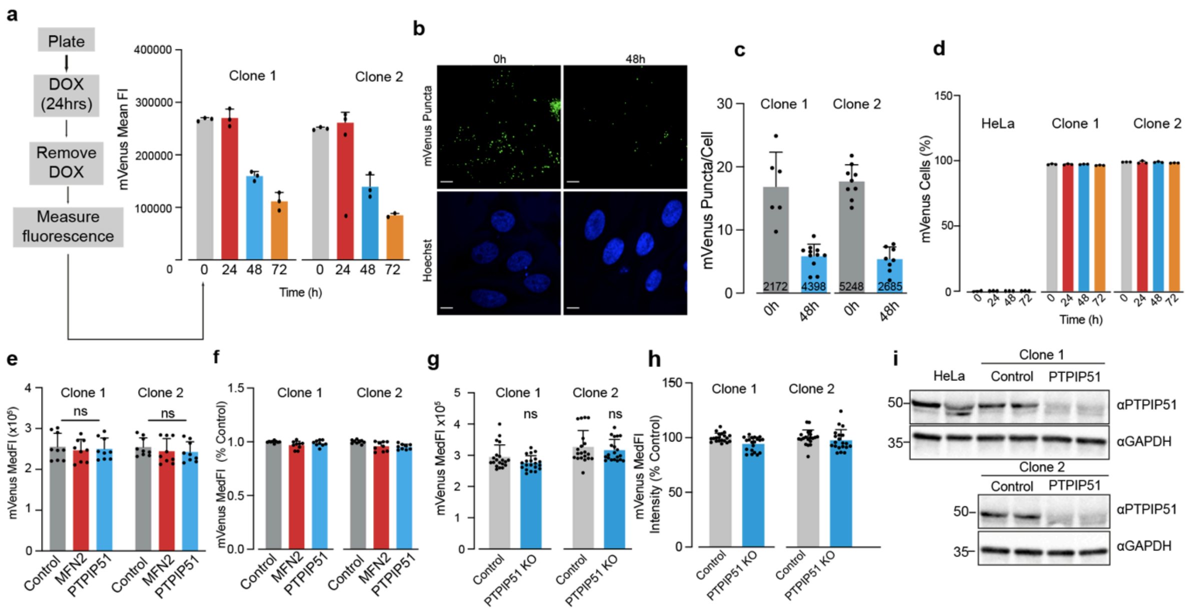

Supplementary Figure 2: Optimisation of the ER-Mito reporter cell line.
(**a**) The optimal mVenus reporter induction conditions are treatment with doxycycline (1 µg/mL) for 24 h followed by removal of doxycycline for 48 h. ER-Mito cells were treated with doxycycline for 24 h, and then basal medium was added for 0 h, 24 h, 48 h or 72 h before analysis of the mean FI by flow cytometry. (**b** and **c**) There is a decrease in mVenus puncta with the addition of 48 h doxycycline-free medium compared to not adding doxycycline-free medium. ER-Mito cells were treated with doxycycline (1 µg/mL) for 24 h, and then doxycycline-free medium was added for either 0 h or 48 h. Representative images of mVenus puncta (**b**) detected by spinning disc confocal microscopy in cells co-stained with Hoechst to detect the nuclei and quantified in (**c**). Scale bar 10 µm, mean ± SD., number indicates number of cell analysed, Clone 1 n = 4 and Clone 2 n = 4, data points represent cover slips (Colone 1 (+0h = 6, +48h = 11), Clone 2 (+0h = 9, +48 = 8). (**d**) mVenus MedFI can be detected in 95-100% of ER-Mito reporter clones. ER-Mito cells were treated with doxycycline (1 µg/mL) for 24 h, and then basal medium was added for 0 h, 24 h, 48 h or 72 h before analysis by flow cytometry. Mean ± SD., (**e** and **f)** A reduction in MFN2 or PTPIP51 does not decrease mVenus MedFI. ER-Mito reporter cells were transfected with non-targeting (control), MFN2 and PTPIP51 siRNA for 3 days and treated with 1 µg/mL doxycycline for 24 h before analysis of MedFI by flow cytometry (**e**) and percentage changed calculated (**f**). Mean ± S.D., data points represent each well analysed, Clone 1 n = 3 and Clone 2 n = 3, data points represent technical replicates for 3 biological replicates, data analysed using Shapiro-Wilk normality test and Two-way ANOVA with Dunnett's multiple comparison test. (**g-i**) Loss of PTPIP51 does not result in a decrease in mVenus MedFI. ER-Mito reporter cells were transduced with either non-targeting (control) or PTPIP51-targeting gRNAs and selected with puro to generate non-targeting or PTPIP51 knockout pools. These were analysed by flow cytometry to examine MedFI (**g**), percentage change (**h**), and protein levels were examined via western blotting (**i**). Mean ± SD, Clone 1 n = 3 and Clone 2 n=3, data points represent technical replicates, ordinary two-way ANOVA with Sidak's multiple comparisons test, with single pooled variance.

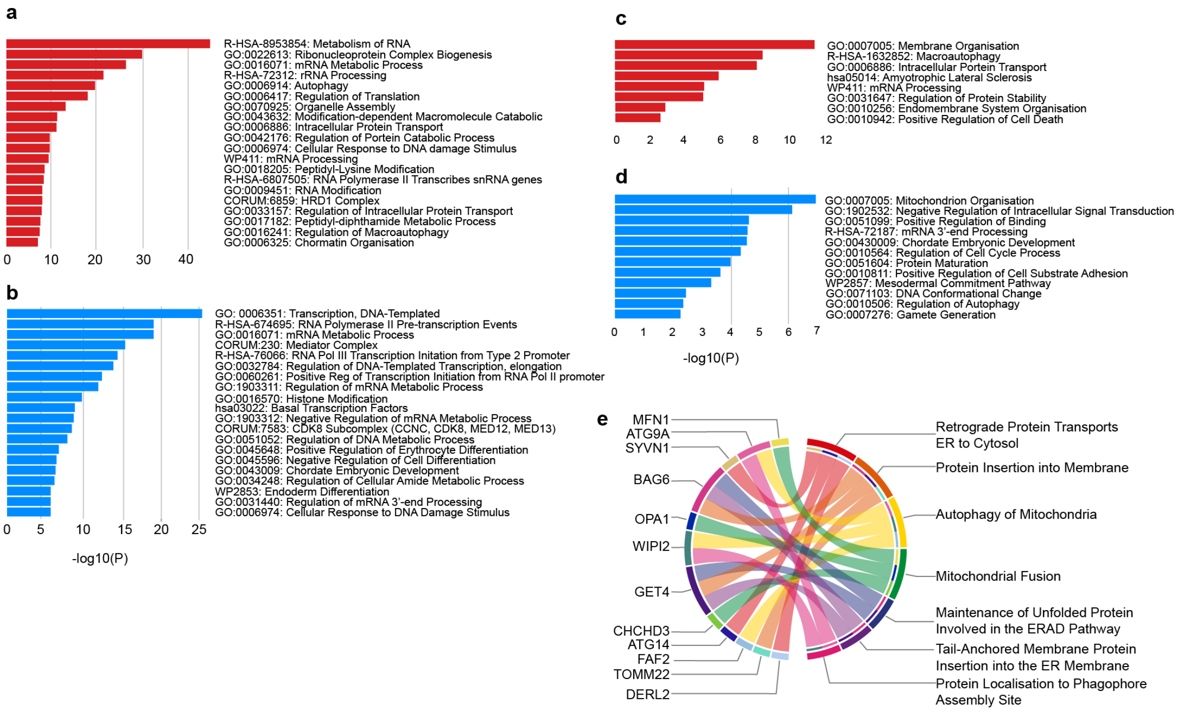

Supplementary Figure 3: Pathway enrichment analysis of genes regulating MERCS

(**a** and **b**) Top pathways enriched in the selected populations of 410 genes that either increase (**a**) MERCS or 230 genes that decrease (**b**) MERCS. (**c** and **d)** Top pathways enriched in the genes selected for validation (29 for each set) in the MERCS increase (**c**) or decrease (**d**) sets. We used Metascape for the analysis with an FDR cut-off of 0.0001 and the adjusted term p value (−log10) is shown**.** Selected results from Gene Ontology (GO) KEGG, GO Biological Processes, Reactome Gene Sets, Canonical Pathways, CORUM, WikiPathways, and PANTHER. **(e**) Pathway analysis of the 29 top selected genes that increase MERCS. Analysis was conducted using STRING and used to generate a cord plot showing the top biological pathways associated with the 29 chosen genes that increase MERCS.

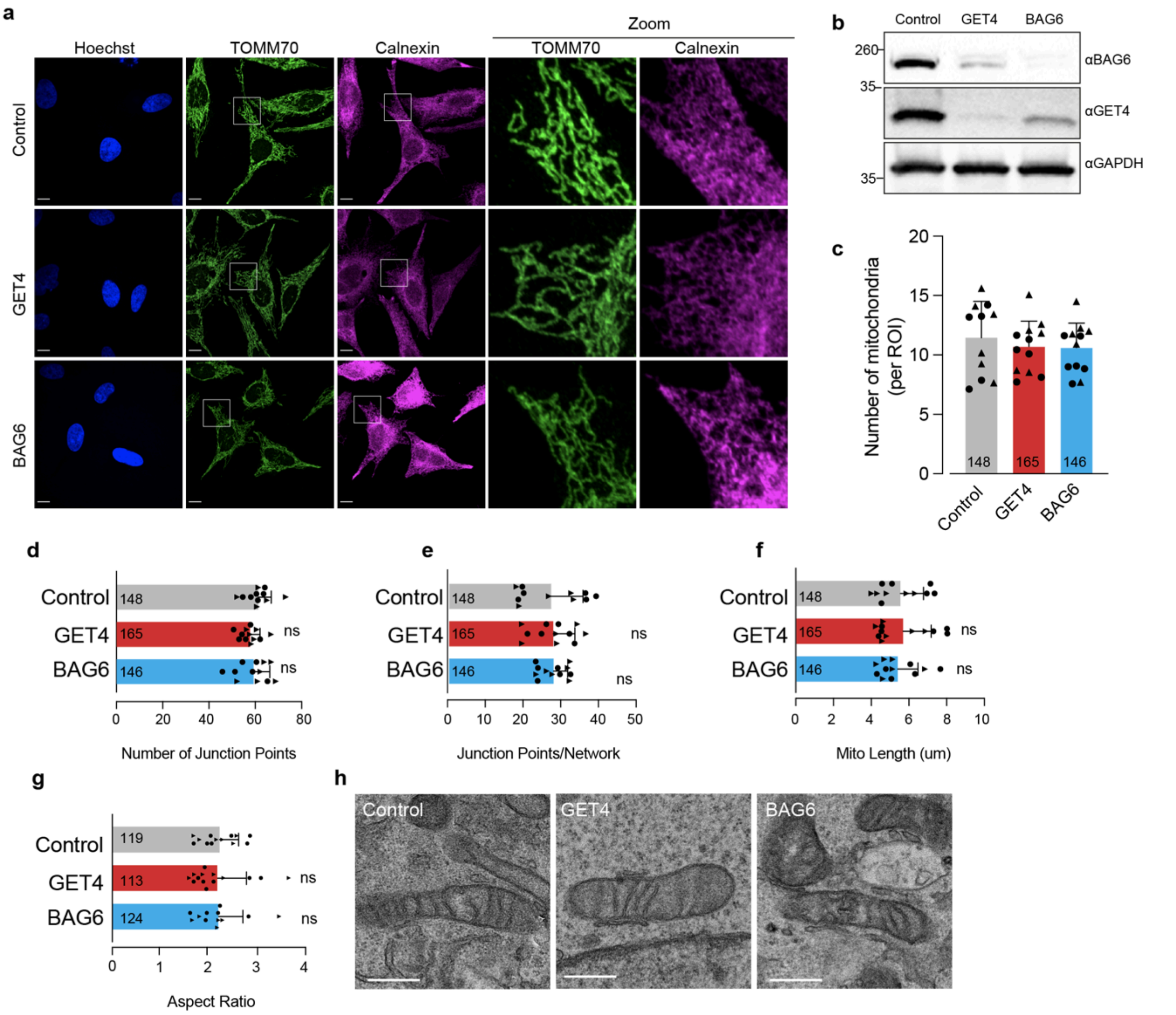

Supplementary Figure 4: Absence of gross ER or mitochondrial morphology changes upon knockdown of GET4 or BAG6

(**a** to **f**) Loss of GET4 or BAG6 does not result in changes to mitochondria or ER morphology. ER-Mito reporter cell lines were transfected with non-targeting (control) GET4 or BAG6 siRNA for 3 days before fixation, staining and analysis. (**a**) Representative immunofluorescence images showing mitochondria stained with an anti-TOMM70 antibody, the ER, stained with an anti-Calnexin antibody and co stained with Hoechst to detect the nuclei. Scale bar 10 µm. (**b**) GET4 and BAG6 show robust knockdown with siRNA transfection. ER-Mito reporter cell lines were transfected with non-targeting (control) GET4 or BAG6 siRNA for 3 days, lysed, analysed by western blotting, and probed with the indicated antibodies. Mitochondrial morphology was analysed using the MitoMAPR[39] tool looking at the number of objects per region of interest (ROI) (no junction points) (**c**), ratio of networks to junction points (**e**) and mitochondrial length (**f**). Scale bar 10 µm. Mean ± S.D., number indicates number of cells analysed, n=4 (2x Clone 1, 2x Clone 2), data points = mitochondria morphology parameters averaged per coverslip (Con = 11, GET4 = 12, BAG6 =12), (circles= Clone 1, triangles = Clone 2), data analysed using mixed effects models with significance tests performed using Satterthwaite's degrees of freedom method with ImerTest. (g) Mitochondrial morphology was analysed examining the aspect ratio (ratio between the height and width) of the mitochondria using electron microscopy images under the loss of GET4 and BAG6. Scale bar 500 nm. Mean ± SD., number indicates number of mitochondria analysed, n=2 (1x Clone 1, 1x Clone 2), data points = aspect ratio averaged of each Mitochondria averaged per cell (Con = 13, GET4 = 14, BAG6 =12), (circles = Clone 1, triangles = Clone 2), data analysed using mixed effects models with significance tests performed using Satterthwaite's degrees of freedom method with ImerTest

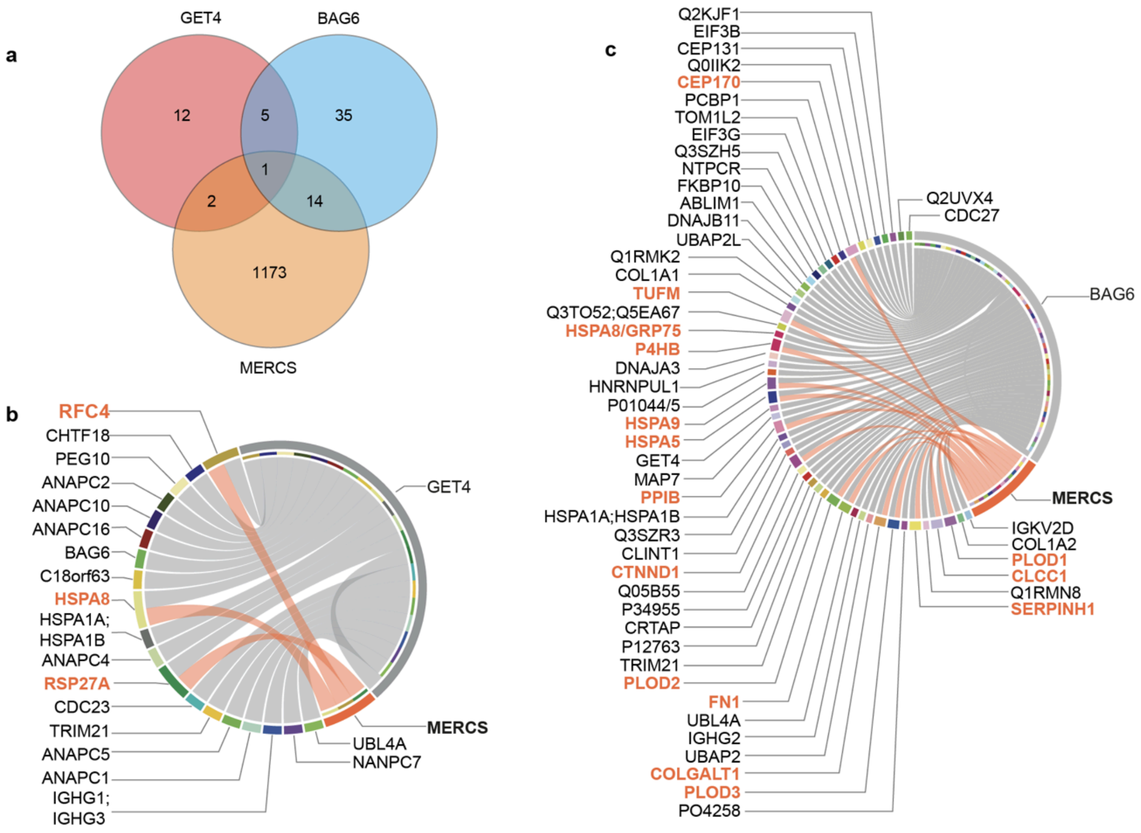

Supplementary Figure 5: Interactors of GET4 or BAG6 are associated with MERCS.
(**a** to **c**) GET4 and BAG6 interactors are associated with MERCS. Co-IP of GET4 and BAG6 was analysed by mass spectrometry and overlapped with proteins found in MERCS. (**a**) A Venn diagram showing the overlap between known MERCS proteins and GET4 or BAG6 interactors. (**b** and **c**) Chord plots representing the STRING analysis of the GET4 (**b**) and BAG6 (**c**) interactors examining their overlap with MERCS proteins.

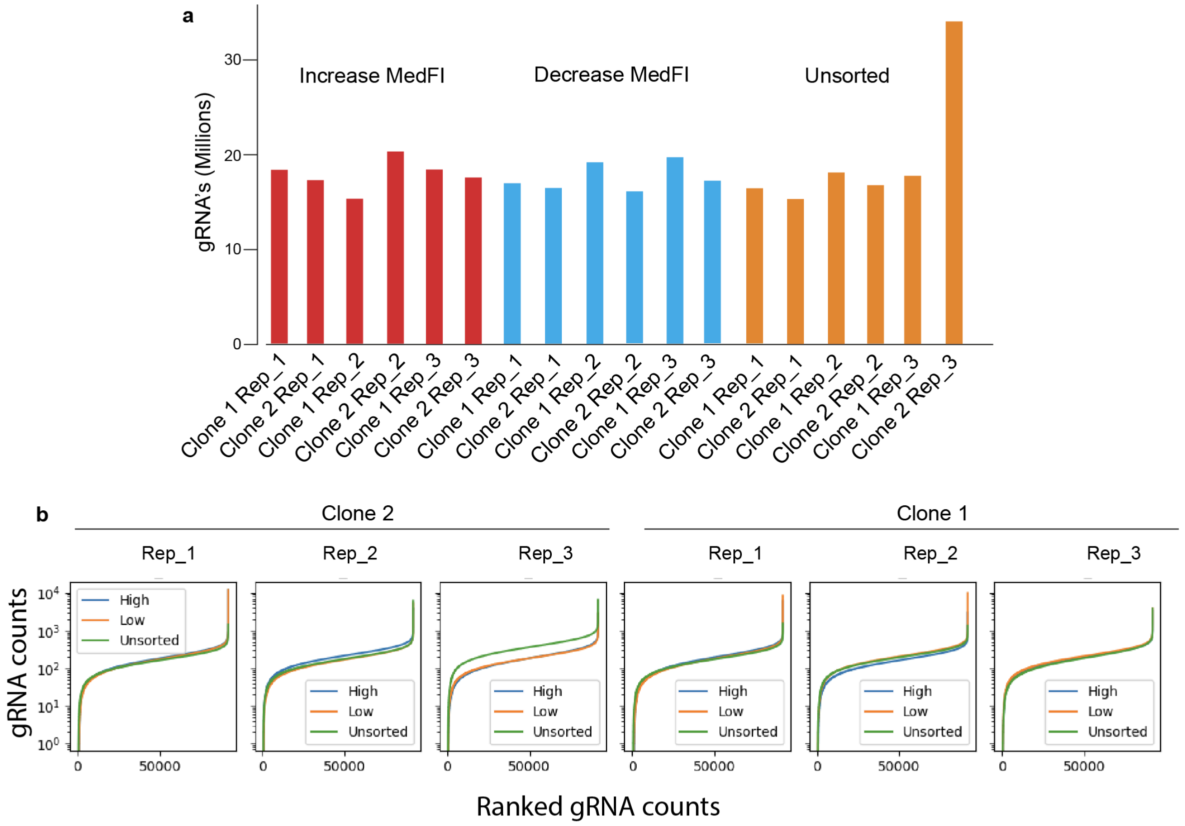

Supplementary Figure 6: Quality control of pooled CRISPR/Cas9 genome-wide screen
(**a**) Number of valid gRNAs per sample acquired after next-generation sequencing. (**b**) gRNA representation plot showing the coverage of the transfected libraries. In each sample, gRNA counts are ranked from lower to higher (x-axis represents rank), and they are plotted against their respective counts (y-axis). Plot panels are grouped according to biological samples, and the different sorted populations are shown by different line colours.

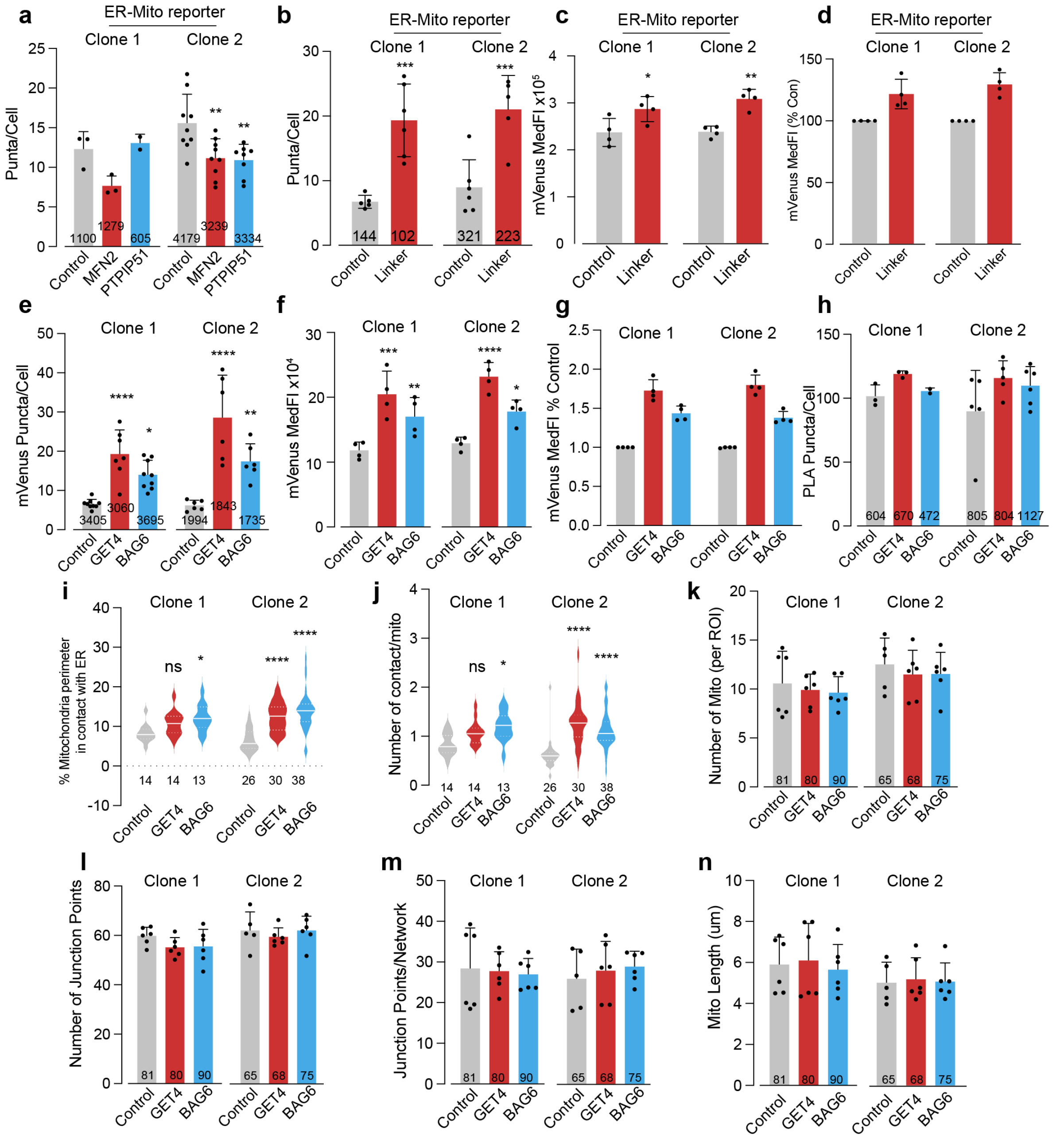

Supplementary Figure 7: mVenus puncta, PLA puncta and MedFI flow cytometry analysis of Clones 1 and Clone 2 individually

(a) The siRNA-mediated downregulation of MFN2 or PTPIP51 decreases the number of mVenus puncta. ER-Mito reporter cells were transfected with non-targeting (control), MFN2 and PTPIP51 siRNA for 34 days and treated with 1 µg/mL doxycycline for 24 h. Images of the mVenus puncta detected by spinning disc confocal microscopy were quantified in (**a**). Mean ± S.D., number indicates number of cells analysed, Clone 1 n = 1, Clone 2 n = 3, data points = mVenus puncta/cell averaged per coverslip (Clone 1 (Con = 3, MFN2 = 3, PTPIP51 =2), Clone 2 (Con = 9, MFN2 = 9, PTPIP51 = 8)), data analysed using Shapiro-Wilk normality test and Two-way ANOVA with Dunnett's multiple comparison test. (**b**) Overexpression of the ER-Mito linker increases mVenus fluorescence intensity. ER-Mito reporter cells were transfected with ER-Mito linker (mAKAP1 [34–63]-mRFP-yUBC6)[68] or control-plasmid expressing mito-mCherry and treated with 1 µg/mL doxycycline for 24 h + 48 h in medium without doxycycline. Representative images of mVenus puncta were detected by spinning disc confocal microscopy and mVenus puncta quantified in (**b**). Mean ± S.D., number indicates number of cells analysed, Clone 1 n = 2, Clone 2 n = 2, data points = mVenus puncta/cell averaged per coverslip ((Clone 1 (Con = 5, Linker = 6), Clone 2 (Con = 6, Linker = 5)) data analysed using Shapiro-Wilk normality test and Two-way ANOVA with Dunnett's multiple

comparison test. (**c** and **d**) Overexpression of the ER-mito linker increases mVenus fluorescence intensity. ER-Mito reporter cells were transfected with ER-Mito linker (mAKAP1 [34–63]-mRFP-yUBC6)[68] and treated with 1 µg/mL doxycycline for 24 h + 48 h in medium without doxycycline. The MedFI was measured by flow cytometry (**c**) and the change relative to control calculated (**d**). Mean ± S.D., Clone 1 n = 4, Clone 2 n = 4, data points = mVenus medFI averaged per biological replicate, data analysed using Shapiro-Wilk normality test and Two-way ANOVA with Dunnett's multiple comparison test. (**e, f** and **g**) Loss of GET4 or BAG6 results in an increase in mVenus puncta and MedFI. ER-Mito reporter cells were transfected with either non-targeting (control), GET4 or BAG6 siRNA for 7 days and treated with 24 h doxycycline (1 µg/mL) + 48 h normal media before analysis. Representative images of the mVenus puncta detected by spinning disc confocal microscopy and mVenus puncta quantified in (**e**). Scale bar 10 µm. Mean ± S.D., number indicates number of cells analysed, Clone 1 n = 3, Clone 2 n = 2, data points = mVenus puncta/cell averaged per coverslip (Clone 1 (Con = 9, GET4 = 7, BAG6 = 9) Clone 2 (Con = 6, GET4 = 6, BAG6 = 6)), data analysed using Shapiro-Wilk normality test and Two-way ANOVA with Dunnett's multiple comparison test. The MedFI was measured using flow cytometry in (**f** and **g**). Mean ± SD, Clone 1 (n = 4), Clone 2 (n = 4), data points = mVenus medFI technical replicates averaged per biological replicate, data analysed using Shapiro-Wilk normality test and Two-way ANOVA with Dunnett's multiple comparison test. (**h**) Loss of GET4 or BAG6 results in an increase in PLA puncta. ER-Mito cells were transfected with non-targeting (control), GET4 and BAG6 siRNA for 3 days, fixed and stained to examine MERCS via PLA. Representative images of the PLA puncta were detected by immunofluorescence and spinning disc microscopy and quantified in (**h**). Mean ± S.D., number indicates number of cells analysed, n = 3 (1x Clone 1, 2x Clone 2), data points = mVenus puncta/cell averaged per coverslip, Clone 1 (Con = 3, GET4 = 3, BAG6 = 2) and Clone 2 (Con = 4, GET4 = 5, BAG6 = 6), data analysed using Shapiro-Wilk normality test and Two-way ANOVA with Dunnett's multiple comparison test. (**i** and **j**) The loss of GET4 and BAG6 results in an increase in the percentage of ER in contact with the mitochondria perimeter and the number of contact sites per mitochondria. ER-Mito cells were transfected with non-targeting (control), GET4 and BAG6 siRNA for 3 days, fixed, embedded and MERCS examined by TEM. The percentage of mitochondrial perimeter in contact with ER and number of contact sites per mitochondrion were quantified in (**i**) and (**j**), respectively. Median ± quartiles., Clone 1; n = 1, Clone 2; n = 2, number in figure represents the number of cells analysed, Mitochondria analysed Clone 1 (Con = 228, GET4 = 205 and BAG6 = 157) and Clone 2 (Con = 499, GET4 = 487 and BAG6 = 578), data analysed using Shapiro-Wilk normality test and Two-way ANOVA with Dunnett's multiple comparison test. (**k** to **n**) The loss of GET4 and BAG6 results in no gross morphological changes in mitochondria. ER-Mito cells were transfected with non-targeting (control), GET4 and BAG6 siRNA for 3 days, fixed, and mitochondrial morphology examined by spinning disc confocal microscopy. Mitochondrial morphology was analysed using the MitoMAPR[39] tool looking at the number of objects per ROI (no junction points) (**k**), ratio of networks to junction points (**l** and **m**) and mitochondrial length (**n**). Mean ± SD., Clone 1 n = 2, Clone 2 n = 2, number in figure represents the number of cells analysed, data analysed using Shapiro-Wilk normality test and Two-way ANOVA with Dunnett's multiple comparison test.

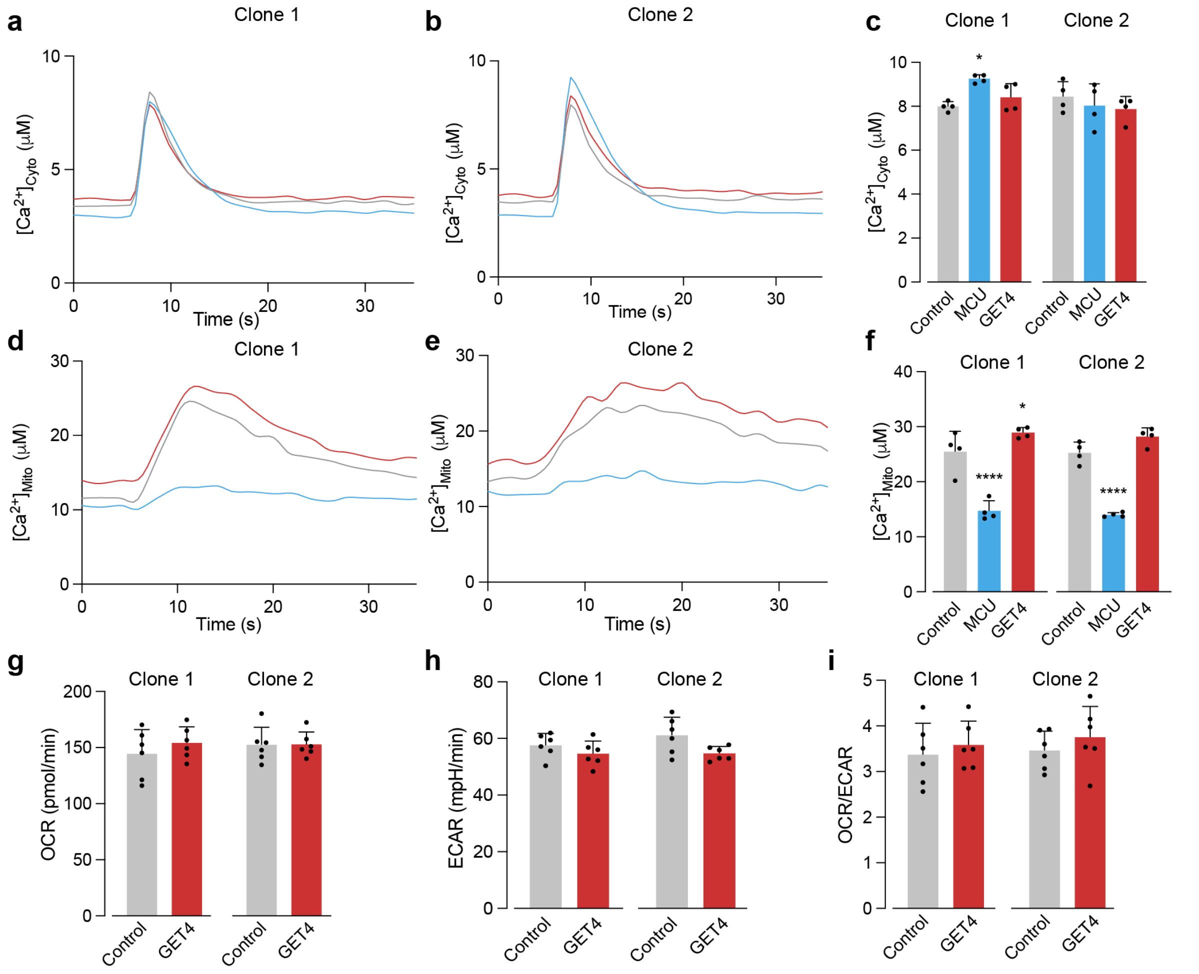

Supplementary Figure 8: Calcium and Seahorse analysis of clones 1 and Clone 2 individually
(**a** to **f**) Loss of GET4 increases mitochondrial $[Ca^{2+}]$. ER-Mito cells were transfected with non-targeting (control), GET4 and MCU siRNA for 3 days and analysed using cytosolic and mitochondrial targeted aequorin probes to measure the cytosolic (**a, b** and **c**) and mitochondrial (**d, e** and **f**) $[Ca^{2+}]$. Curves showing the cytosolic $[Ca^{2+}]$ over the first 35 s for Clone 1 (**a**) and Clone 2 (**b**) with max peak of $[Ca^{2+}]$ for either Clone 1 or Clone 2 shown in (**c**). Mean ± S.D., Clone 1 ($n = 4$) and Clone 2 ($n = 4$). Independent analysis of clones using, using Shapiro-Wilk normality test and Two-way ANOVA with Dunnett's multiple comparison test. Curves showing the mitochondrial $[Ca^{2+}]$ over the first 35 s for Clone 1 (**a**) and Clone 2 (**b**) with max peak of mitochondrial $[Ca^{2+}]$ for either Clone 1 or Clone 2 shown in (**c**). Mean ± S.D., Clone 1 ($n = 4$) and Clone 2 ($n = 4$). Independent analysis of clones using, using Shapiro-Wilk normality test and Two-way ANOVA with Dunnett's multiple comparison test (**g**, **h** and **i**) The loss of GET4 increases basal respiration. ER-Mito reporter cells were transfected with non-targeting (control) and GET4 siRNA for 3 days, and the OCR and ECAR were determined via a seahorse mitochondrial stress assay. Change in the OCR (**g**), ECAR (**h**) and OCR/ECAR ratio (**i**) assessed over 20 min. Time points 14 and 21 were combined per clone. Mean ± S.D., Clone 1 ($n = 3$) and Clone 2 ($n = 3$). Independent analysis of clones using, using Shapiro-Wilk normality test and Two-way ANOVA with Dunnett's multiple comparison test.

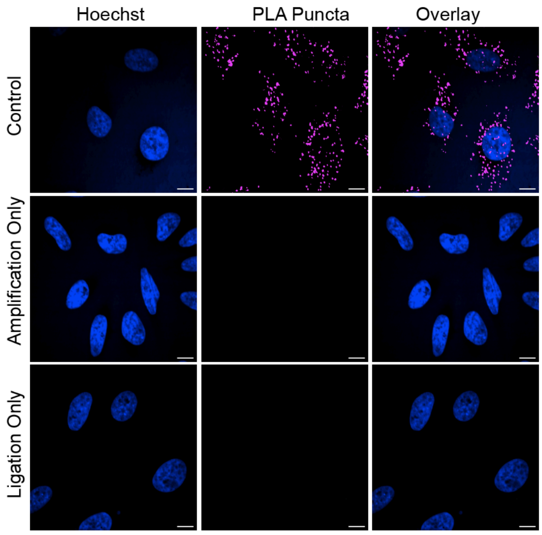

Supplementary Figure 9: PLA Ligation and Amplification Controls
The PLA puncta are specific to GRP75 and VDAC puncta and not associated with ligation or amplification steps. ER-Mito cells were fixed and stained to examine MERCS via PLA. Representative images of the PLA puncta were detected by immunofluorescence and spinning disc microscopy. All images were taken with the same exposure.

Supplementary Table Legends

Supplementary Table 1: Whole genome pooled library
A table showing the sequence and relevant information for all gRNAs used in the whole genome pooled screen. (a) Chromosome number of the giRNA targets, (b) gRNA start position in the genome, (c) gRNA end position in the genome, (d) DNA strand gRNA targeting, (e) gRNA DNA sequence forwards strand, (f) PAM sequence, (g) gRNA DNA sequence on reverse strand, (h) gene name/identifier, and (i) exon identifier.

Supplementary Table 2: Analysis of whole-genome pooled CRISPR/Cas9 screen
A table showing the results of the whole-genome pooled screen displayed by genes (sheet 1) or gRNAs (sheet 2). (a) gRNA identifier, (b) gene name, (c) total number of gRNAs per gene (5 gRNAs per gene per replica (5x6 = 30), (d) score for gRNAs enriched in the population which had a decrease in MedFI, (e) p value for gRNAs enriched in the population which had a decrease in MedFI, (f) false discovery rate for gRNAs enriched in the population which had a decrease in MedFI, (g) rank for gRNAs enriched in the population which had a decrease in MedFI, (h) number of different gRNA;s that were found to be enriched in population which had a decrease in MedFI, (i) Log fold change of gRNAs enriched in the population which had an increase in MedFI, (j) score for gRNAs enriched in the population which had an increase in MedFI, (k) p value for gRNAs enriched in the population which had an increase in MedFI, (l) false discovery rate for gRNAs enriched in the population which had an increase in MedFI, (m) rank for gRNAs enriched in the population which had an increase in MedFI, (n) number of different gRNA;s that were found to be enriched in population which had an increase in MedFI, and (o) Log fold change of gRNAs enriched in the population which had an increase in MedFI

Supplementary Table 3: The Arrayed gRNA Library
This table shows the DNA sequences for the arrayed library used in this study. (a) Gene name, (b) gRNA identifier in WG library, (c) forwards DNA sequence of gRNA including complementary overhangs (lowercase) for BFP puro backbone cut with BBS1, and (d) reverse DNA sequence of gRNA including complementary overhangs (lowercase) for BFP puro backbone cut with BBS1.

Supplementary Table 4: IP-Mass Spectrometry results investigating interactors of GET4 or BAG6.

Supplementary Table 5: Interactors of GET4
Table of gene names (a) with p values (b) and the difference in fold change compared to control. Those that overlap with MERCS proteins are stated in Column (d) and highlighted in bold. Those proteins that overlap with BAG6 interactors are stated in Column (e) and underlined.

Supplementary Table 6: Interactors of BAG6
Table of gene names (a) and the difference in fold change compared to control (b). Those that overlap with MERCS proteins are stated in Column (c) and highlighted in bold. Those proteins that overlap with GET4 interactors are stated in Column (d) and underlined.

Supplementary Table 7: siRNAs used in this study

Supplementary Table 8: Antibodies used in this study
