# Supplementary material for: Genome-wide CRISPR/Cas9 screen shows that loss of GET4 increases mitochondria-endoplasmic reticulum contact sites and is neuroprotective
Source: Cell Death Dis. 2024 Mar 11;15(3):203. doi: 10.1038/s41419-024-06568-y (PMC10928201; doi:10.1038/s41419-024-06568-y)
Supplement: Supplementary file 10 — Full Western Blot Scans [file 41419_2024_6568_MOESM10_ESM.pdf]

Figure 1c: MFN2 and PTPIP51 knockdown

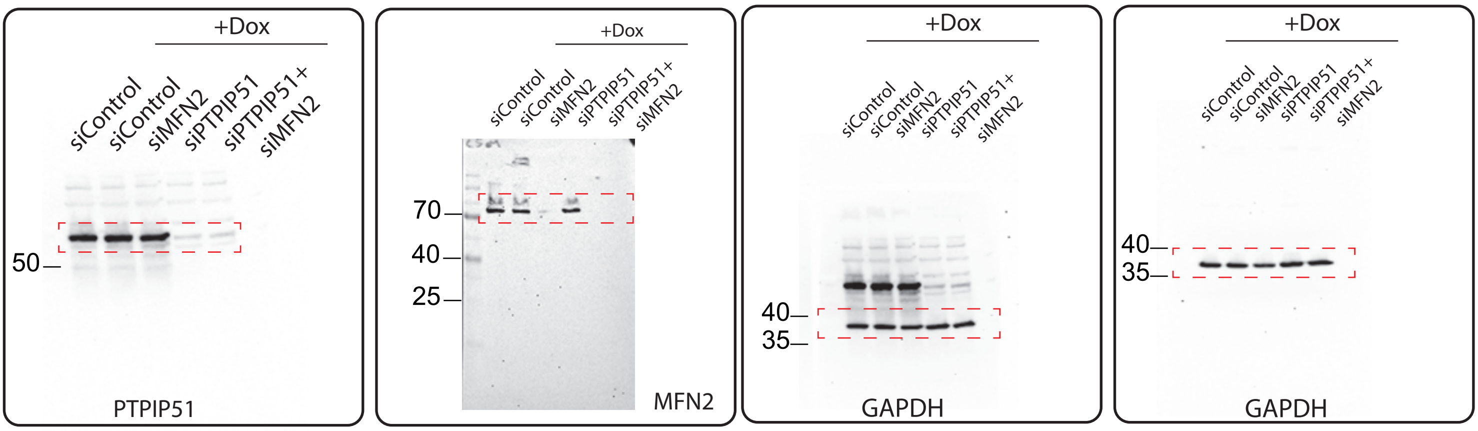

Figure 3d: GET4 and BAG6 knockdown after 7 days

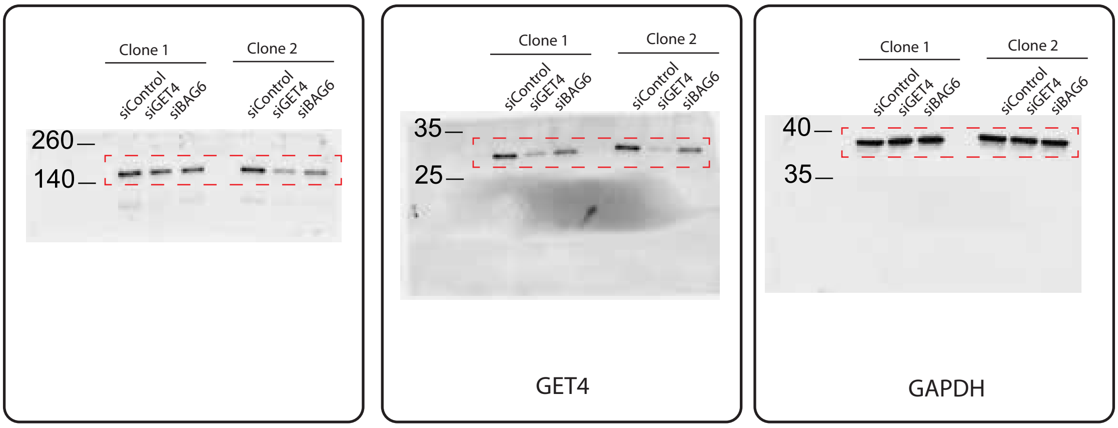

Figure 3d: GET4 and BAG6 knockdown after 3 days

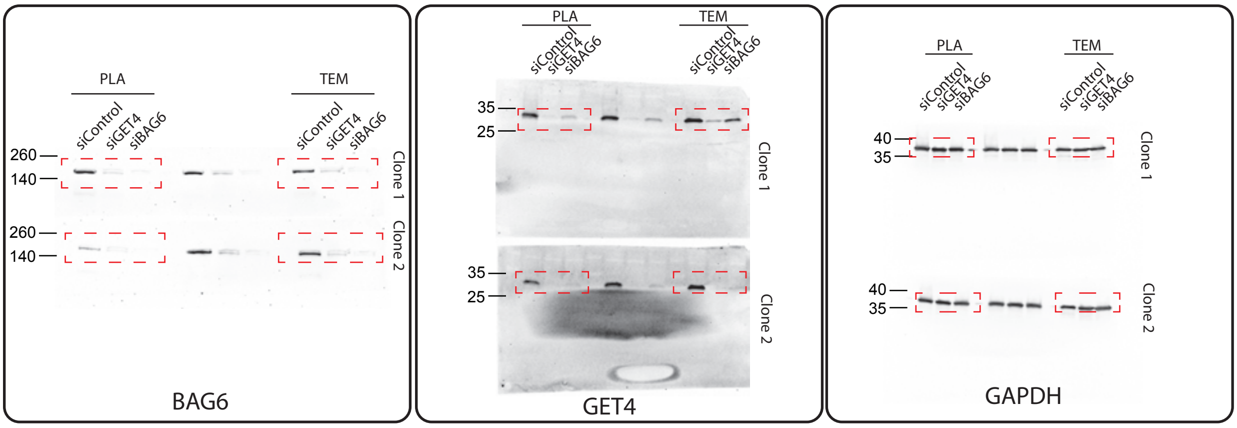

Figure 4a and b: GET4 and BAG6 interact with IP3R and GRP75 Respectivly

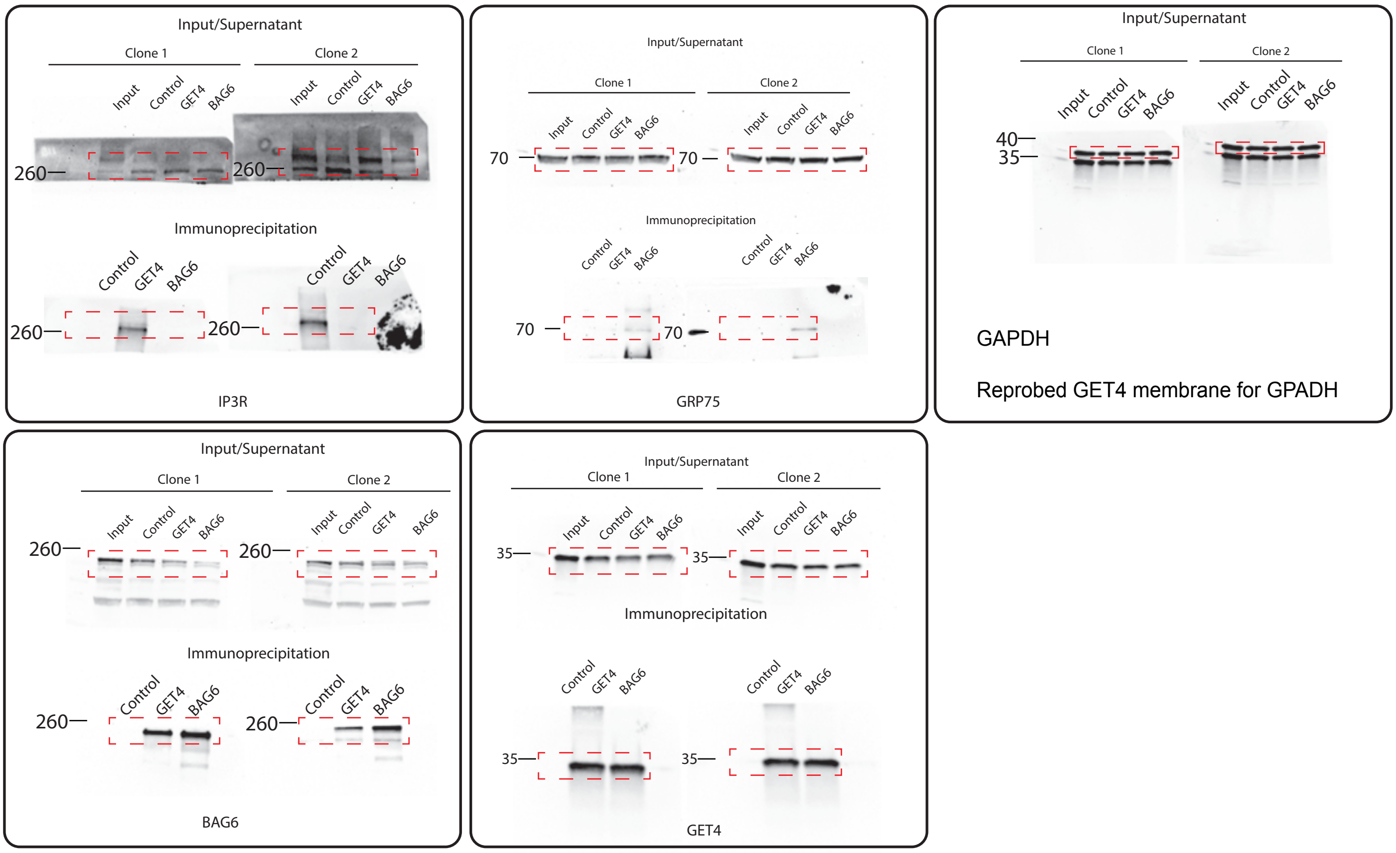

Figure 4a and b: GET4 and BAG6 interact does not interact with MFN2

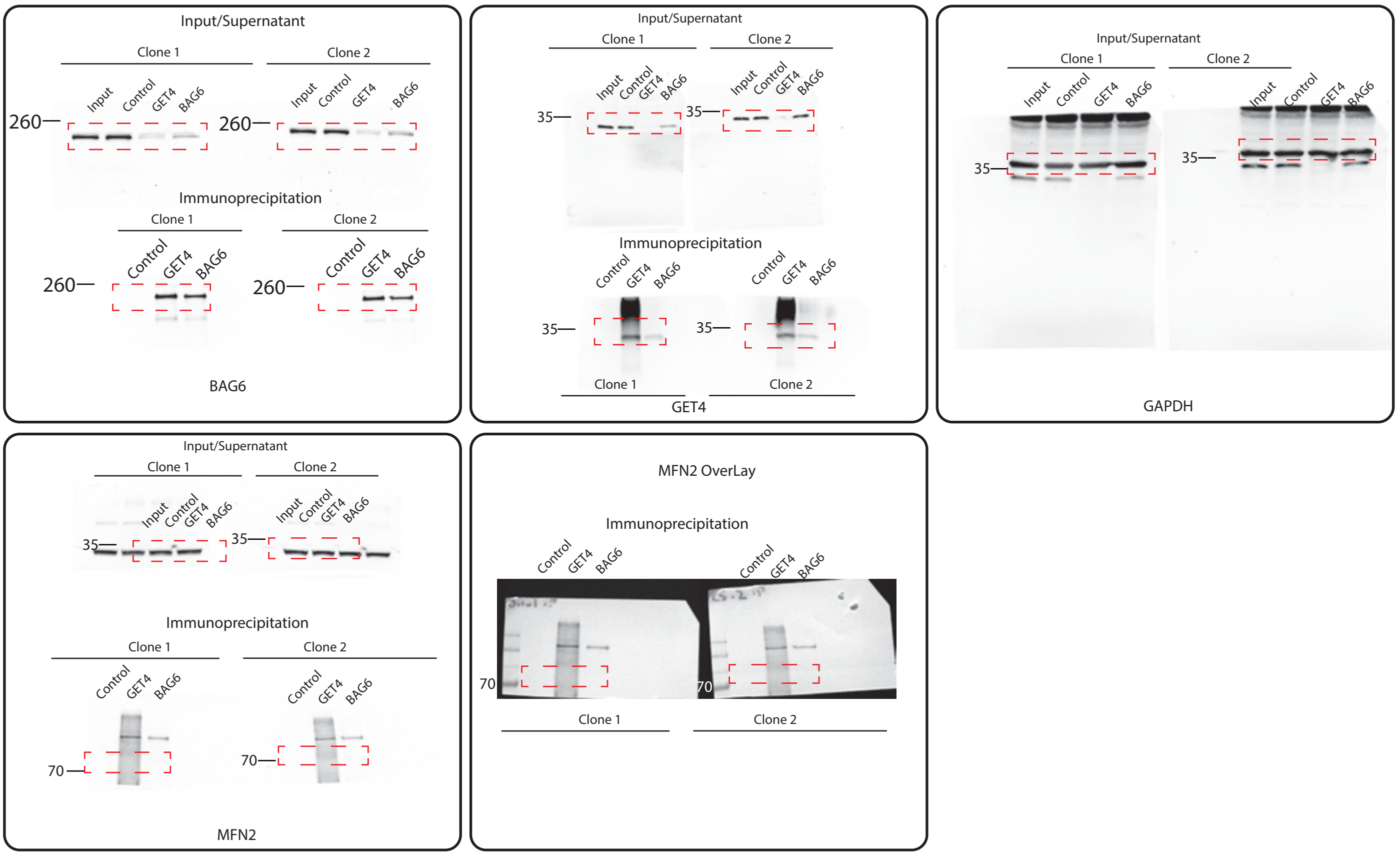

Figure 4c: GET4 and BAG6 interact in samples sent to Mass spec.

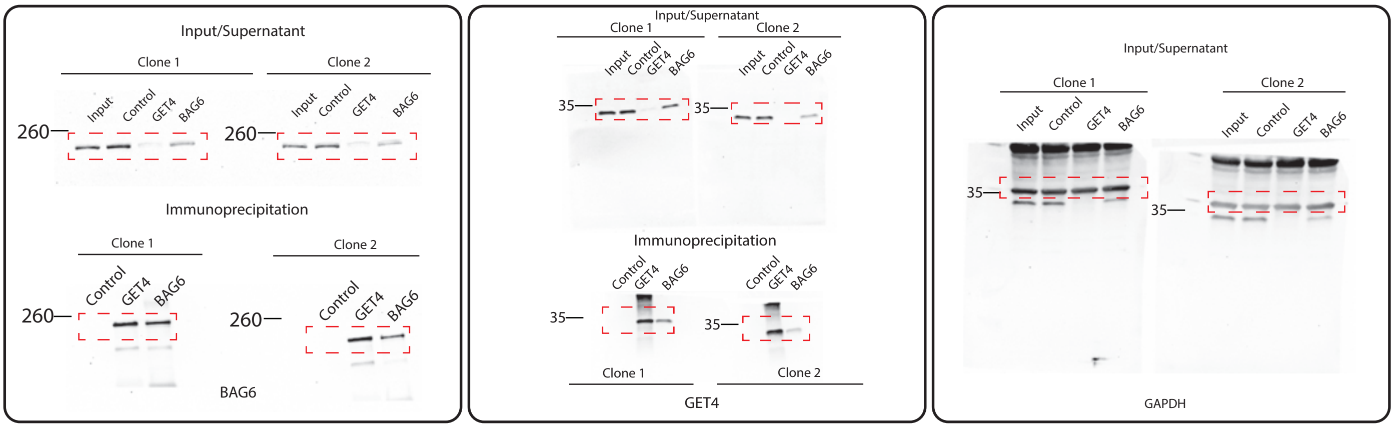

Figure 6c: GET4 Does not alter MICU1 or MICU2.

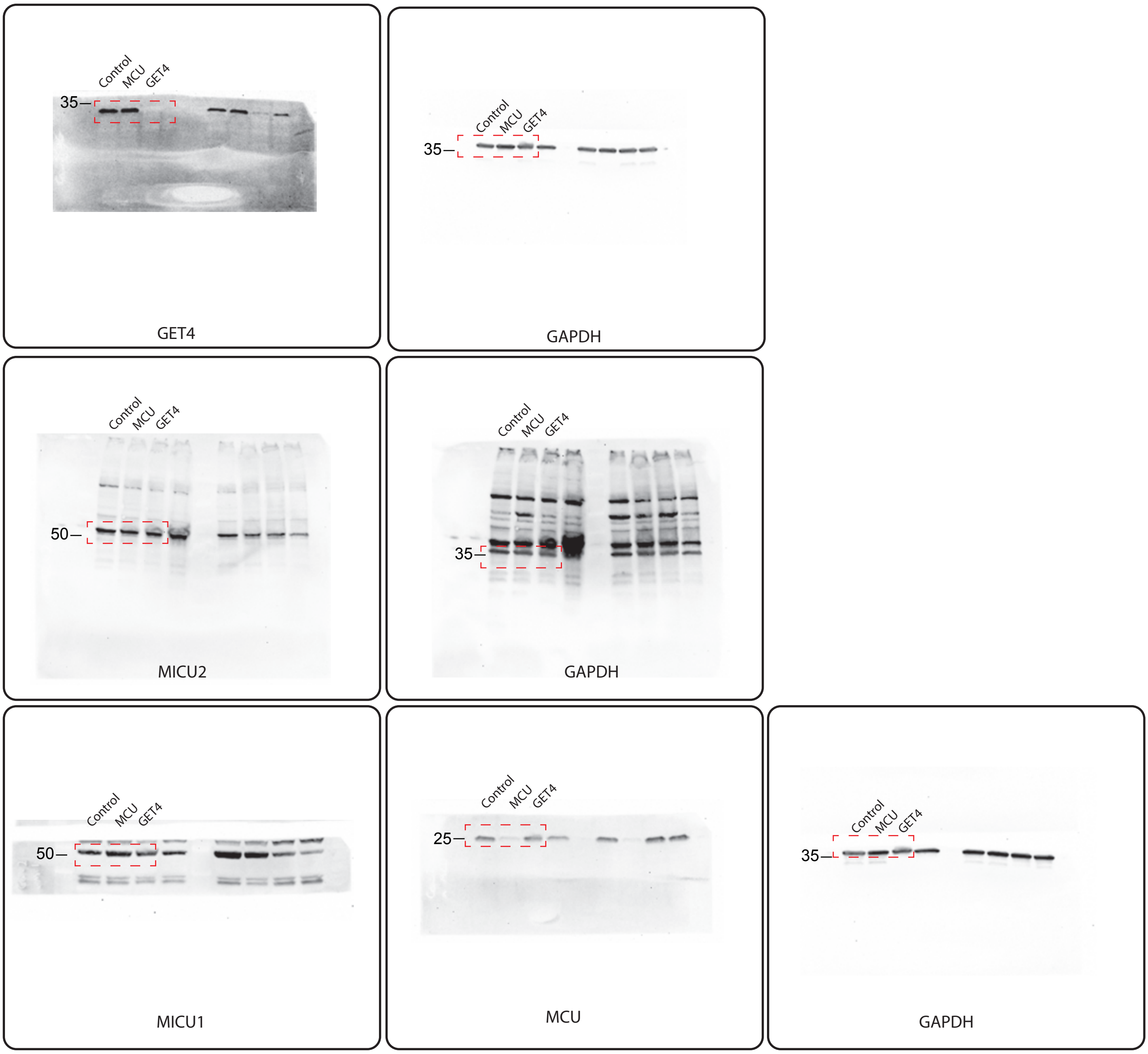

Figure 4a: GET4 and BAG6 can localise to MAM

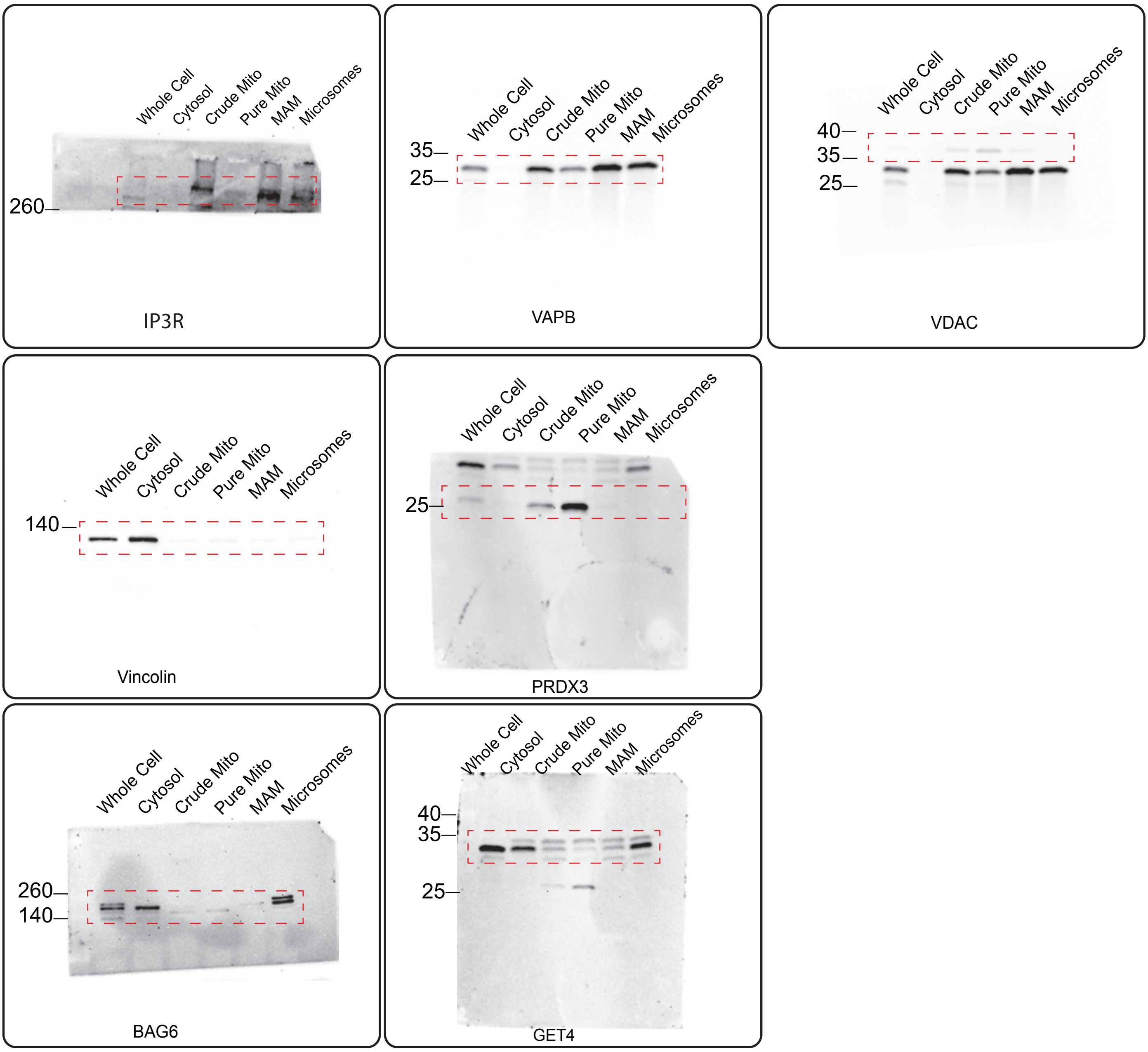

Figures 3 j and k BAG6 and GET4 knockdown

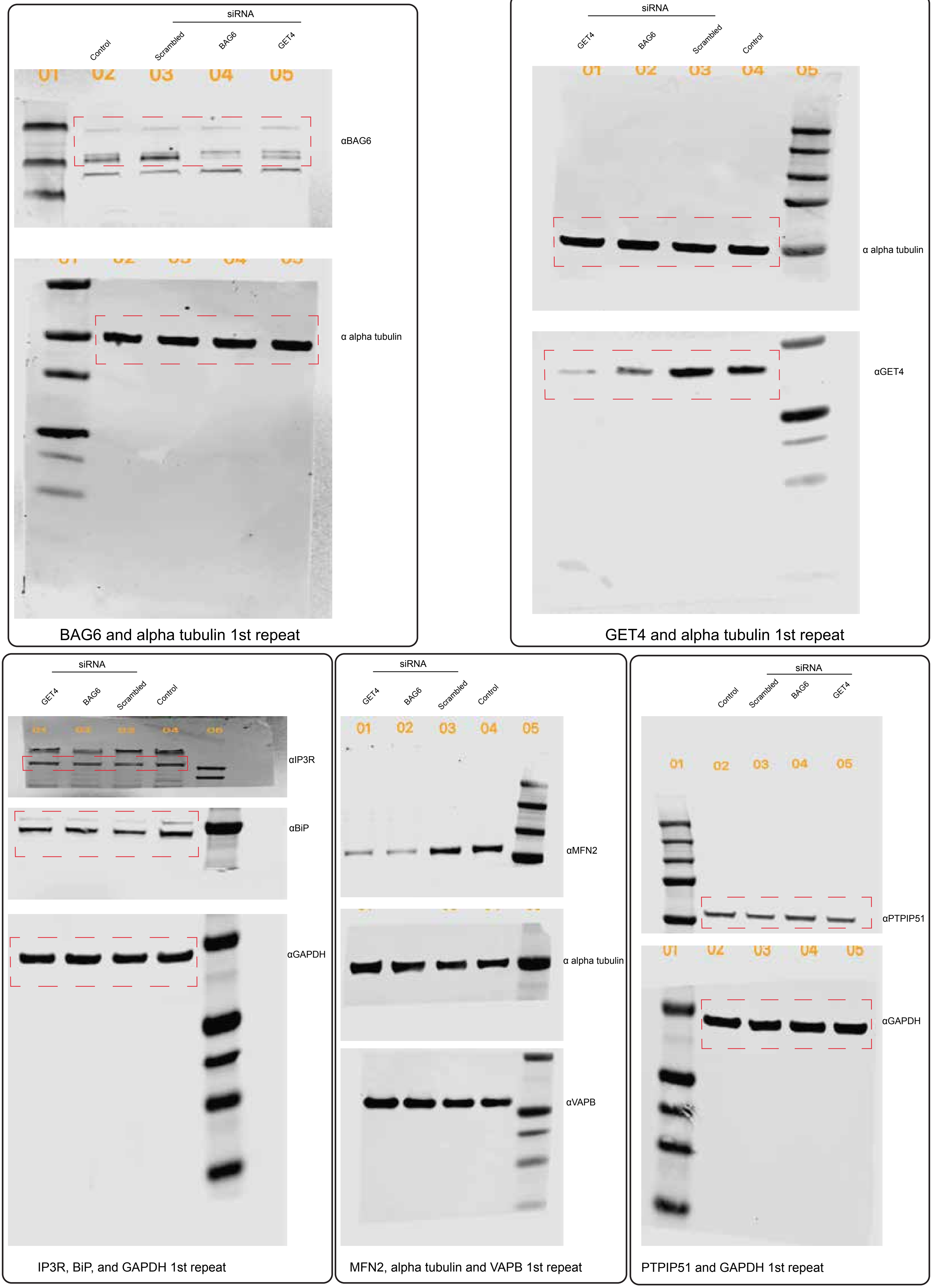

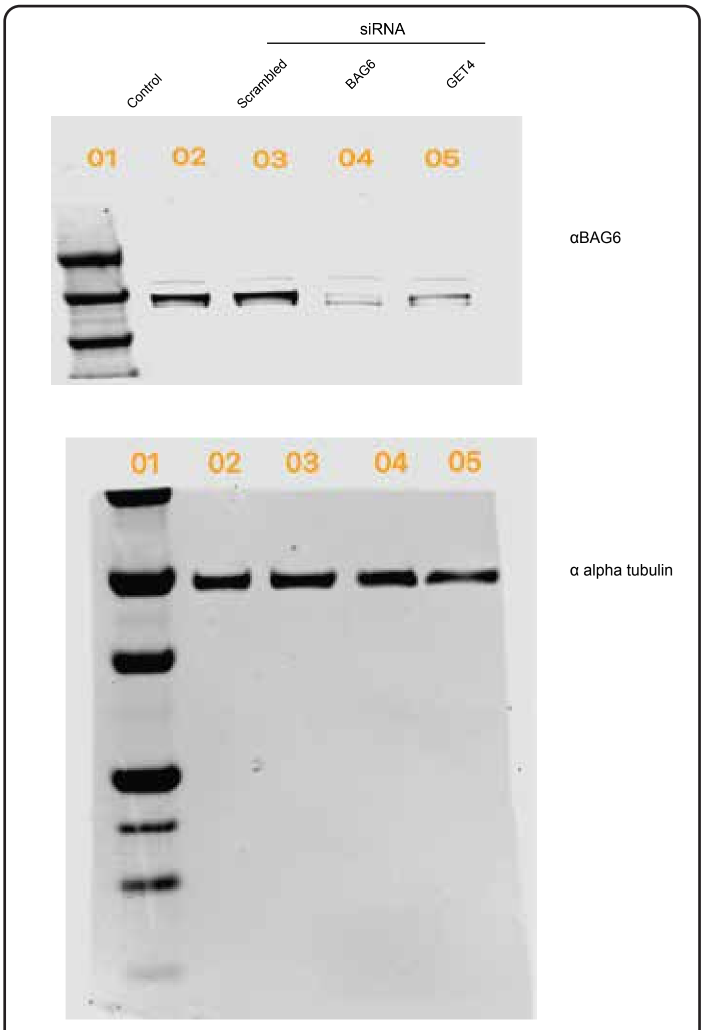

BAG6 and alpha tubulin 2nd repeat

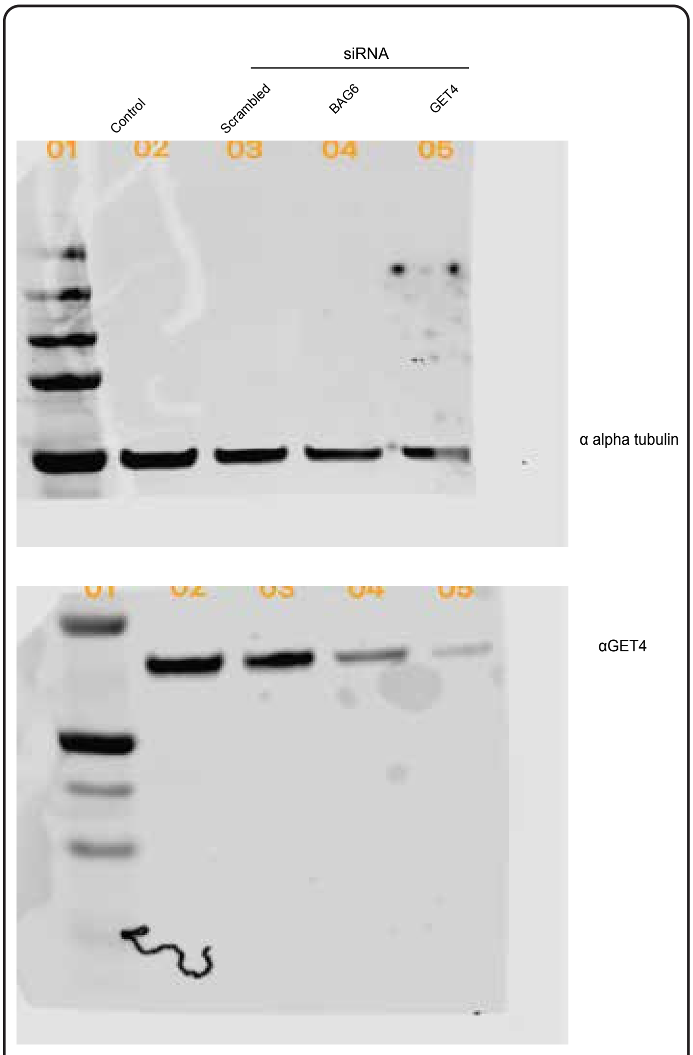

GET4 and alpha tubulin 2nd repeat

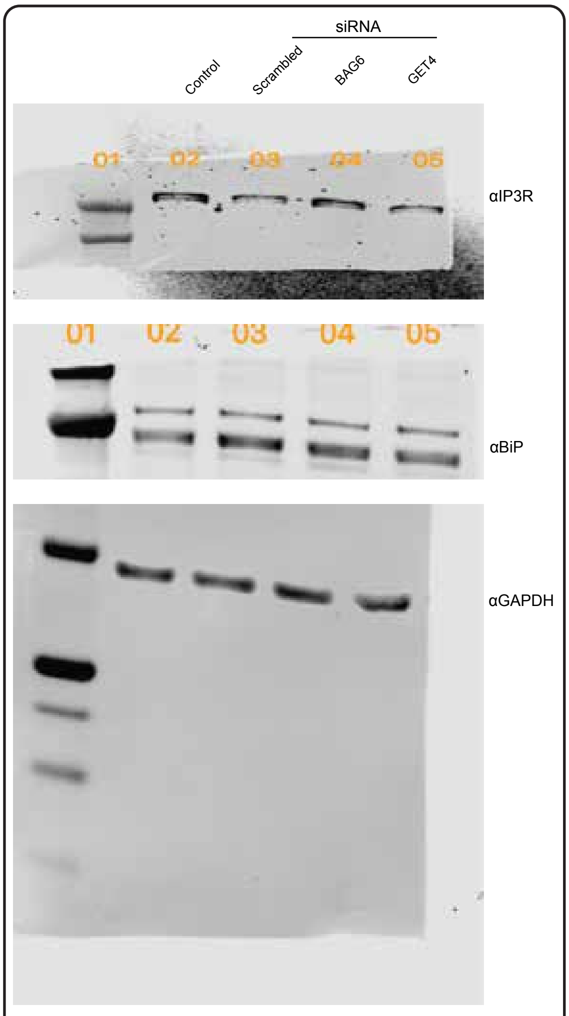

IP3R, BiP, and GAPDH 2nd repeat

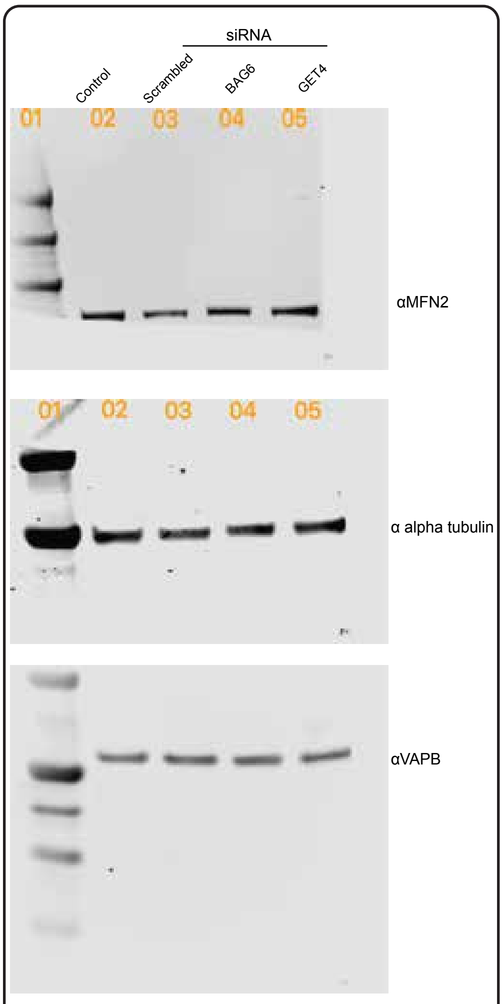

MFN2, alpha tubulin and VAPB 2nd repeat

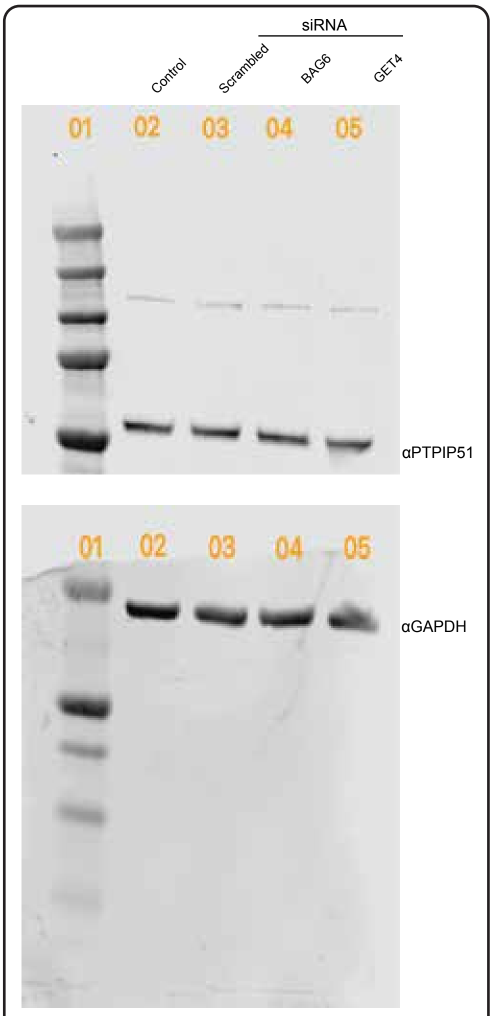

PTPIP51 and GAPDH 2nd repeat

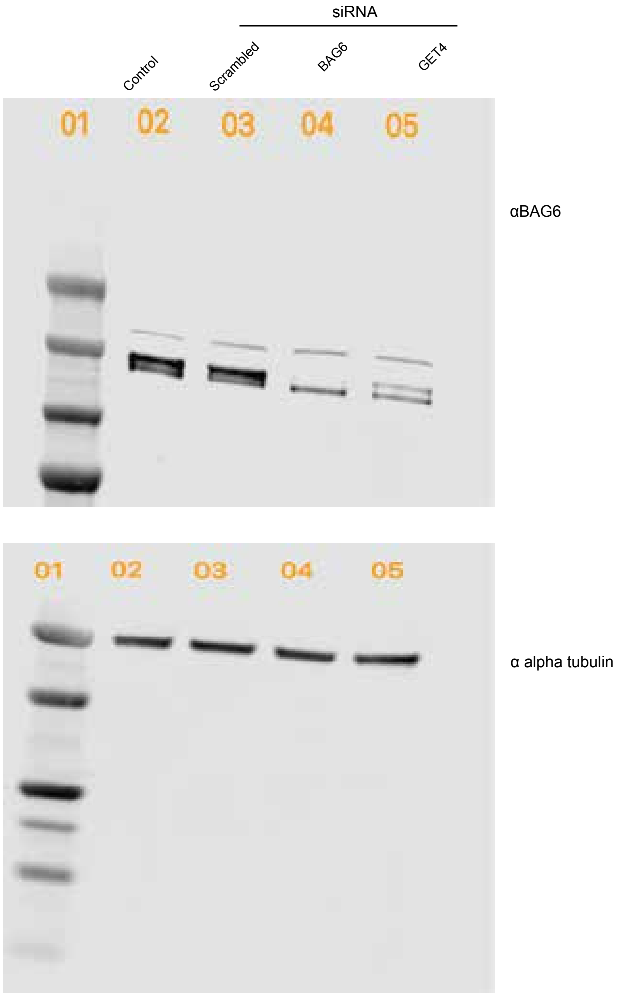

BAG6 and alpha tubulin 3rd repeat

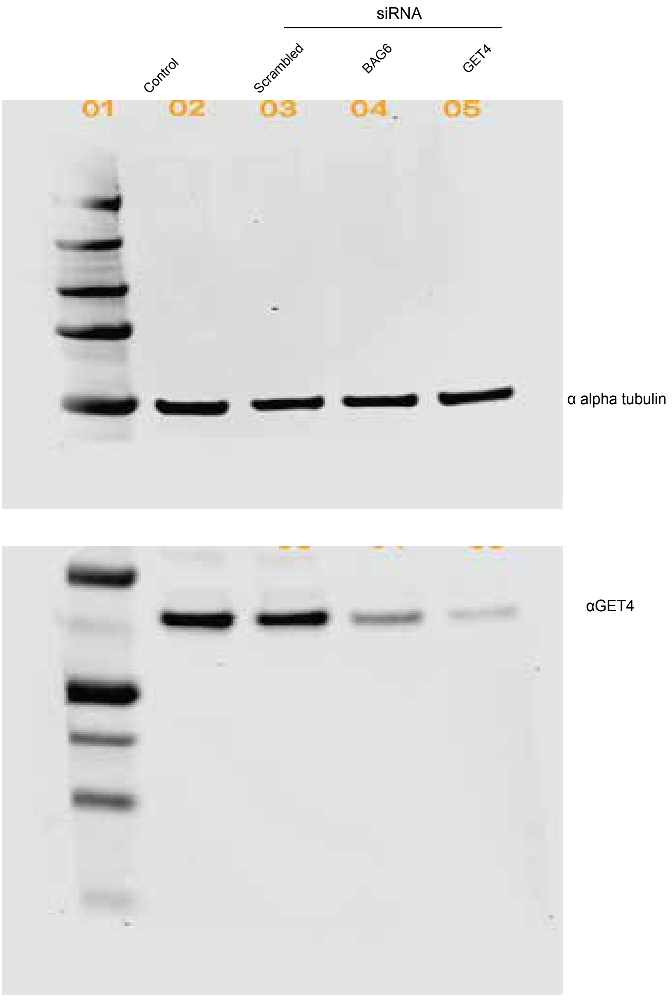

GET4 and alpha tubulin 3rd repeat

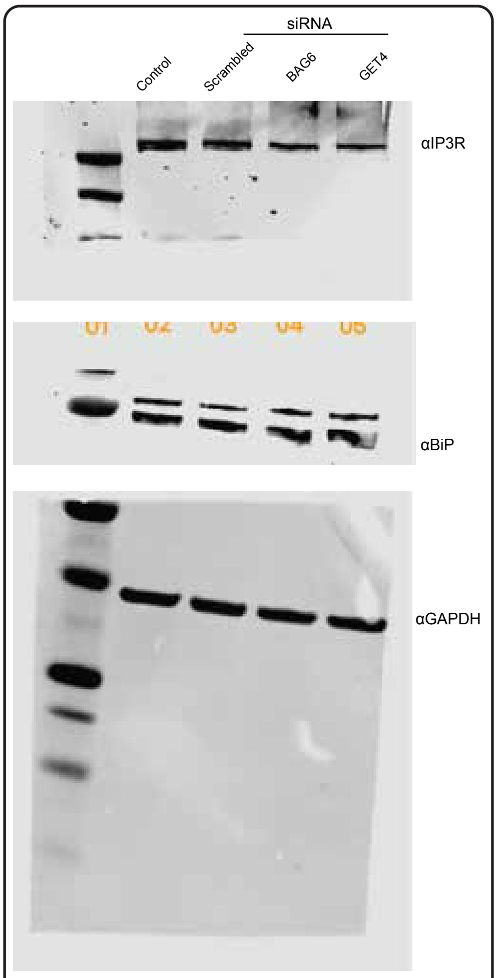

IP3R, BiP, and GAPDH 3rd repeat

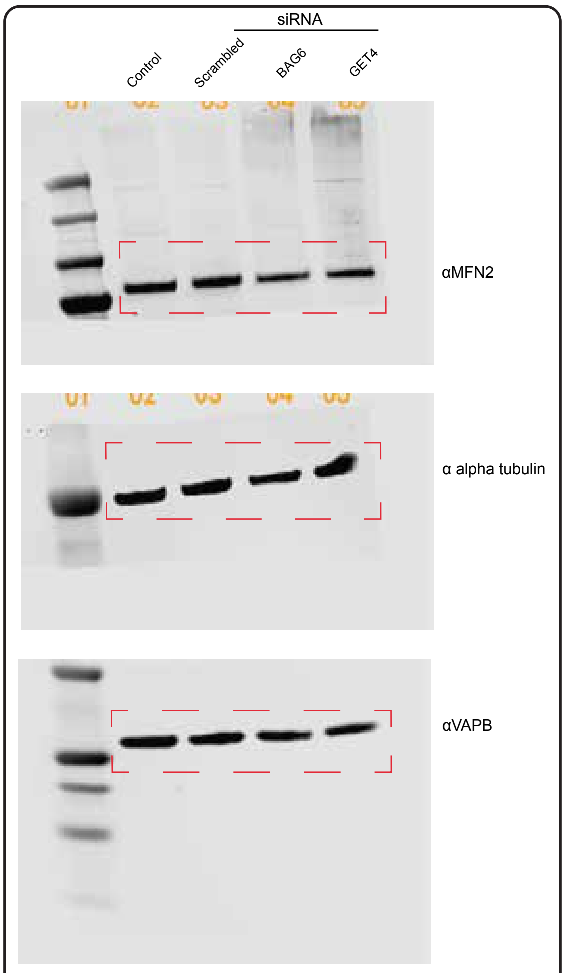

MFN2, alpha tubulin and VAPB 3rd repeat

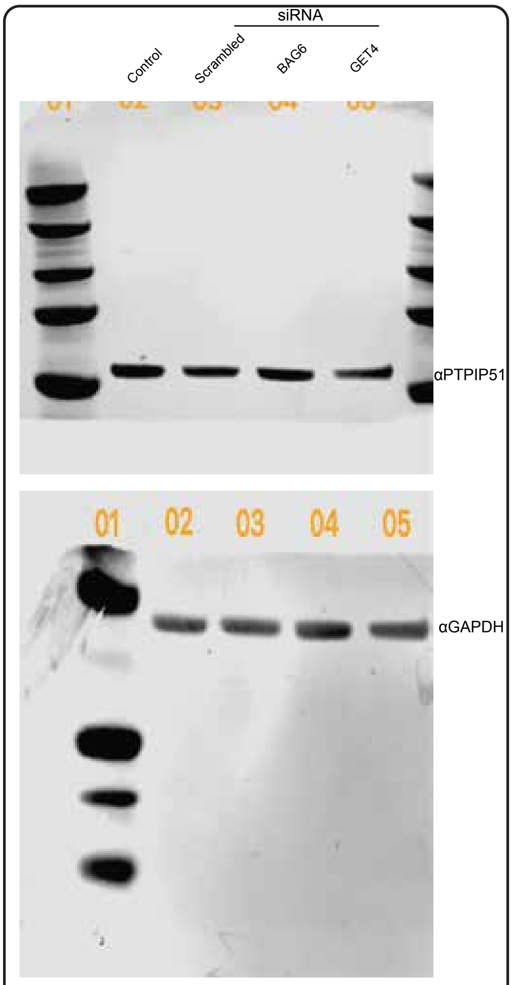

PTPIP51 and GAPDH 3rd repeat
