# Supplementary material for: Genome-wide CRISPR/Cas9 screen shows that loss of GET4 increases mitochondria-endoplasmic reticulum contact sites and is neuroprotective
Source: Cell Death Dis. 2024 Mar 11;15(3):203. doi: 10.1038/s41419-024-06568-y (PMC10928201; doi:10.1038/s41419-024-06568-y)
Supplement: Supplementary file 12 — Author Change Approval [file 41419_2024_6568_MOESM12_ESM.pdf]

bit.bio
THE CELL CODING COMPANY

Manos Metzakopian <manos.metzakopian@bit.bio>

URGENT: AUTHOR CHANGE APPROVAL REQUIRED::Re: Decision letter for CDDIS-23-2444-T

19 messages

Manos Metzakopian <manos.metzakopian@bit.bio> Sat, Dec 2, 2023 at 2:58 PM
To: Emma L Wilson <elwilson92@gmail.com>, yizhou0421@gmail.com, sf26@sanger.ac, plumbwong@gmail.com, jlm96@cam.ac.uk, vjop2@mrc-mbu.cam.ac.uk, njs76@cam.ac.uk, src78@mrc-mbu.cam.ac.uk, gl515@cam.ac.uk, pra29@cam.ac.uk, Julien Prudent <julien.prudent@mrc-mbu.cam.ac.uk>, m795@cam.ac.uk, shyl2@cam.ac.uk, npatikas@bwh.harvard.edu, yma24@cam.ac.uk
Cc: jaw209@cam.ac.uk

Dear Authors,

It is great pleasure to inform you that we are now ready to submit the manuscript revisions and reply to reviewers.
In order to finalise the new data we have had an excellent contribution of experiment and analysis done by James Woodward (cced) with which we have increased the quality of the manuscript and he will be added as an author on the author list.

Please reply to this email confirming your approval to this change in authorship as it is a requirement by the journal for progress of the manuscript in the review process and final acceptance if successful.

See journal rule here: *"Requests to change the authorship (such as to include or exclude an author, change an author's name or contribution) must be accompanied by a letter signed by all authors to show they concur with the change. New authors must also confirm that they fully comply with the journal's authorship requirements."*

Thank you for your quick reply and assistance! And congratulations once more on such great work on scientific progress of this manuscript.

Best regards,
Manos

On Fri, Oct 13, 2023 at 2:52 PM Emmanouil Metzakopian <mano982@gmail.com> wrote:

> Dear all,
>
> The reviewer comments are in. Please see below.
>
> I believe they are good and we can reply to them fairly quickly.
>
> Please share your thoughts and let's have a meeting to discuss.
>
> I want to thank you all again for the great effort we all put in and especially Emma!
>
> Best regards,
> Manos
>
> Begin forwarded message:
>
> **From:** cddisease@springernature.com
> **Date:** 13 October 2023 at 14:44:58 BST
> **To:** mano982@gmail.com
> **Subject: Decision letter for CDDIS-23-2444-T**
> **Reply-To:** cddisease@springernature.com
>
> Dear Dr Metzakopian
> RE: Manuscript CDDIS-23-2444-T, Genome-wide CRISPR/Cas9 screen shows that loss of GET4 increases mitochondria-endoplasmic reticulum contact sites and is neuroprotective. by Dr Metzakopian
>
> Many thanks for sending us the above-mentioned manuscript which has now been seen by expert referees.
>
> In view of their comments, your manuscript cannot be accepted in its present form. The referees have, however, made some suggestions that will help you to produce a revised version within 2 months. Please notify the editorial office if you need more time than this.
>
> Once these points have been fully addressed, your manuscript will be re-assessed. We cannot, of course, guarantee its final acceptance. Please note that the present *Cell Death & Disease* policy allows only one round of revision.
>
> Please ensure you include the following with your submission:
>
> - A rebuttal letter with a point-by-point response to each of the reviewer comments and a description of changes made.
> - A marked-up version of the manuscript with the changes highlighted in yellow. Please do not include track changes or comments. Please select the file type Revised Manuscript - Marked Up.
> - A clean version of the manuscript in Word with line numbers. Please select the file type Article File.
> - A reproducibility checklist that details key elements of the experimental and analytical design of the submission. This checklist is found at: http://www.nature.com/authors/policies/checklist_AJ.pdf . It is the author's responsibility to complete this checklist, which should be filled out to include all information relevant to your submission. The form must be uploaded with your manuscript and will be made available to the editor and reviewers.
> - Full and uncropped western blots, uploaded as 'Supplemental Material' & cited in the manuscript. Please note that, should your article be accepted for publication, the Supplementary Material that includes the original western blots will be published alongside the article.
> - Please do not include previous versions of your manuscript in your revised submission, by taking care to ensure the previous files are replaced on the submission site.
>
> *Cell Death & Disease* is committed to improving transparency in authorship. As part of our efforts in this direction, we are now requesting that all authors identified as 'corresponding author' create and link their Open Researcher and Contributor Identifier (ORCID) with their account on the Manuscript Tracking System prior to acceptance. ORCID helps the scientific community achieve unambiguous attribution of all scholarly contributions. You can create and link your ORCID from the home page of the Manuscript Tracking System by clicking on 'Modify my Springer Nature account' and following the instructions in the link below. Please also inform all co-authors that they can add their ORCIDs to their accounts and that they must do so prior to acceptance.
>
> https://www.springernature.com/gp/researchers/orcid/orcid-for-nature-research

- In Figure 6 – The authors indicate that the loss of GET4 increased mitochondrial calcium uptake upon ER-Ca2+ release and mitochondrial respiration. It is a valid way to measure, however, it needs additional evidence by using single cell measurements with appropriate mitochondrial fluorophores. I think it is difficult to assure the increase in mitochondrial calcium without these measurements.

Another important point is related to the direct effect of GET4 in mitochondrial function, as mentioned above. Authors could comment.

- In Figure 7 – Authors show that loss of GET4 rescues motor ability, improves lifespan and prevents neurodegeneration a Drosophila model of Alzheimer's disease (Aβ42Arc). Please, explain figure 7f, differences were not clear among images.

This email has been sent through the NPG Manuscript Tracking System NY-610A-NPG&MTS

Confidentiality Statement:

This e-mail is confidential and subject to copyright. Any unauthorised use or disclosure of its contents is prohibited. If you have received this email in error please notify our Manuscript Tracking System Helpdesk team at http://platformsupport.nature.com .

Details of the confidentiality and pre-publicity policy may be found here http://www.nature.com/authors/policies/confidentiality.html

Privacy Policy | Update Profile

--

Manos Metzakopian
VP, Research and Development

T +44 (0)1223 787297
manos.metzakopian@bit.bio

The Dorothy Hodgkin Building
Babraham Research Campus
Cambridge CB22 3FH
United Kingdom

info@bit.bio | www.bit.bio
Follow us

bit.bio
THE CELL CODING COMPANY

Notice: This message is the property of Bit Bio Ltd and contains information that may be confidential and/or privileged. If you are not the intended recipient, you should not use, disclose or take any action based on this message. If you have received this transmission in error, please immediately contact the sender by return e-mail and delete this e-mail, and any attachments, from any computer.

Manos Metzakopian <manos.metzakopian@bit.bio> Sat, Dec 2, 2023 at 3:01 PM
To: nunojsleal@gmail.com
Cc: Emma L Wilson <elwilson92@gmail.com>

[Quoted text hidden]

Yusuf Adia <yma24@cam.ac.uk> Sat, Dec 2, 2023 at 6:51 PM
To: Manos Metzakopian <manos.metzakopian@bit.bio>

Dear Manos,

I approve this change in authorship.

Best Wishes

Yusuf

From: Manos Metzakopian <manos.metzakopian@bit.bio>
Sent: Saturday, December 2, 2023 2:58 PM
To: Emma L Wilson <elwilson92@gmail.com>; yizhou0421@gmail.com; sf26@sanger.ac; plumbwong@gmail.com; Jordan Morris <jlm96@cam.ac.uk>; vjop2@mrc-mbu.cam.ac.uk; Nuno Santos Leal <njs76@cam.ac.uk>; src78@mrc-mbu.cam.ac.uk; Georgina Elizabeth Lindop <gl515@cam.ac.uk>; Philip Antrobus <pra29@cam.ac.uk>; Julien Prudent <julien.prudent@mrc-mbu.cam.ac.uk>; m795@cam.ac.uk; Sam Loh <shyl2@cam.ac.uk>; npatikas@bwh.harvard.edu; Yusuf Adia <yma24@cam.ac.uk>
Cc: James Woodward <jaw209@cam.ac.uk>
Subject: URGENT: AUTHOR CHANGE APPROVAL REQUIRED::Re: Decision letter for CDDIS-23-2444-T

Dear Authors,

It is great pleasure to inform you that we are now ready to submit the manuscript revisions and reply to reviewers.

In order to finalise the new data we have had an excellent contribution of experiment and analysis done by James Woodward (cced) with which we have increased the quality of the manuscript and he will be added as an author on the author list.

Please reply to this email confirming your approval to this change in authorship as it is a requirement by the journal for progress of the manuscript in the review process and final acceptance if successful.

See journal rule here: *"Requests to change the authorship (such as to include or exclude an author, change an author's name or contribution) must be accompanied by a letter signed by all authors to show they concur with the change. New authors must also confirm that they fully comply with the journal's authorship requirements."*

Thank you for your quick reply and assistance! And congratulations once more on such great work on scientific progress of this manuscript.

Best regards,

Manos

Manos Metzakopian

[Quoted text hidden]

manos.metzakopian@bit.bio

The Dorothy Hodgkin Building
Babraham Research Campus
Cambridge CB22 3FH
United Kingdom

info@bit.bio | www.bit.bio

Follow us

THE CELL CODING COMPANY

Notice: This message is the property of Bit Bio Ltd and contains information that may be confidential and/or privileged. If you are not the intended recipient, you should not use, disclose or take any action based on this message. If you have received this transmission in error, please immediately contact the sender by return e-mail and delete this e-mail, and any attachments, from any computer.

[Quoted text hidden]
[Quoted text hidden]
[Quoted text hidden]

Yizhou Yu <yizhou0421@gmail.com> Sat, Dec 2, 2023 at 7:02 PM
To: Manos Metzakopian <manos.metzakopian@bit.bio>
Cc: Emma L Wilson <elwilson92@gmail.com>, sf26@sanger.ac, plumbwong@gmail.com, jlm96@cam.ac.uk, vjop2@mrc-mbu.cam.ac.uk, njs76@cam.ac.uk, src78@mrc-mbu.cam.ac.uk, gl515@cam.ac.uk, pra29@cam.ac.uk, Julien Prudent <julien.prudent@mrc-mbu.cam.ac.uk>, m795@cam.ac.uk, shyl2@cam.ac.uk, npatikas@bwh.harvard.edu, yma24@cam.ac.uk, jaw209@cam.ac.uk

Dear Manos,

Many thanks again for allowing me to be part of this great collaboration. I am of course happy for James Woodward to be in the author list.
Congratulations to all for a great manuscript.

Best wishes
Yizhou

On Sat, Dec 2, 2023 at 2:58 PM Manos Metzakopian <manos.metzakopian@bit.bio> wrote:

> Dear Authors,
>
> It is great pleasure to inform you that we are now ready to submit the manuscript revisions and reply to reviewers.
> In order to finalise the new data we have had an excellent contribution of experiment and analysis done by James Woodward (cced) with which we have increased the quality of the manuscript and he will be added as an author on the author list.
>
> **Please reply to this email confirming your approval to this change in authorship** as it is a requirement by the journal for progress of the manuscript in the review process and final acceptance if successful.
>
> See journal rule here: *"Requests to change the authorship (such as to include or exclude an author, change an author's name or contribution) must be accompanied by a letter signed by all authors to show they concur with the change. New authors must also confirm that they fully comply with the journal's authorship requirements."*
>
> Thank you for your quick reply and assistance! And congratulations once more on such great work on scientific progress of this manuscript.
>
> Best regards,
> Manos
>
> [Quoted text hidden]

Patikas, Nikolaos <npatikas@bwh.harvard.edu> Sun, Dec 3, 2023 at 2:33 AM
To: Manos Metzakopian <manos.metzakopian@bit.bio>

Sounds good Manos

Manos Metzakopian <manos.metzakopian@bit.bio> writes:

> External Email - Use Caution
>
> Dear Authors,
>
> It is great pleasure to inform you that we are now ready to submit the manuscript revisions and reply to reviewers.
> In order to finalise the new data we have had an excellent contribution of experiment and analysis done by James Woodward (cced)
> with which we have increased the quality of the manuscript and he will be added as an author on the author list.
>
> Please reply to this email confirming your approval to this change in authorship as it is a requirement by the journal for
> progress of the manuscript in the review process and final acceptance if successful.
>
> See journal rule here: "Requests to change the authorship (such as to include or exclude an author, change an author's name or
> contribution) must be accompanied by a letter signed by all authors to show they concur with the change. New authors must also
> confirm that they fully comply with the journal's authorship requirements."

>
> Thank you for your quick reply and assistance! And congratulations once more on such great work on scientific progress of this
> manuscript.
>
> Best regards,
> Manos
>
> Manos Metzakopian
> VP, Research and Development
>
> T +44 (0)1223 787297
>
>
> manos.metzakopian@bit.bio
> The Dorothy Hodgkin Building
> Babraham Research Campus
> Cambridge CB22 3FH
> United Kingdom
>
> info@bit.bio | www.bit.bio
> Follow us * *
> bit.bio
> Notice: This message is the property of Bit Bio Ltd and contains information that may be confidential and/or privileged. If
> you are not the intended recipient, you should not use, disclose or take any action based on this message. If you have
> received this transmission in error, please immediately contact the sender by return e-mail and delete this e-mail, and
> any attachments, from any computer.
> *
> *
> bit.bio
> On Fri, Oct 13, 2023 at 2:52 PM Emmanouil Metzakopian <mano982@gmail.com> wrote:
>
> Dear all,
>
> The reviewer comments are in. Please see below.
>
> I believe they are good and we can reply to them fairly quickly.
>
> Please share your thoughts and let's have a meeting to discuss.
>
> I want to thank you all again for the great effort we all put in and especially Emma!
>
> Best regards,
> Manos
>
> Begin forwarded message:
>
> From: cddisease@springernature.com
> Date: 13 October 2023 at 14:44:58 BST
> To: mano982@gmail.com
> Subject: Decision letter for CDDIS-23-2444-T
> Reply-To: cddisease@springernature.com
>
> Dear Dr Metzakopian
> RE: Manuscript CDDIS-23-2444-T, Genome-wide CRISPR/Cas9 screen shows that loss of GET4 increases
> mitochondria-endoplasmic reticulum contact sites and is neuroprotective. by Dr Metzakopian
>
> Many thanks for sending us the above-mentioned manuscript which has now been seen by expert referees.
>
> In view of their comments, your manuscript cannot be accepted in its present form. The referees have, however, made
> some suggestions that will help you to produce a revised version within 2 months. Please notify the editorial office if you
> need more time than this.
>
> Once these points have been fully addressed, your manuscript will be re-assessed. We cannot, of course, guarantee its final
> acceptance. Please note that the present Cell Death & Disease policy allows only one round of revision.
>
> Please ensure you include the following with your submission:
>
> * A rebuttal letter with a point-by-point response to each of the reviewer comments and a description of changes made.
>
> * A marked-up version of the manuscript with the changes highlighted in yellow. Please do not include track changes or
> comments. Please select the file type Revised Manuscript - Marked Up.
>
> * A clean version of the manuscript in Word with line numbers. Please select the file type Article File.
>
> * A reproducibility checklist that details key elements of the experimental and analytical design of the submission. This
> checklist is found at: http://www.nature.com/authors/policies/checklist_AJ.pdf . It is the author's responsibility to
> complete this checklist, which should be filled out to include all information relevant to your submission. The form must be
> uploaded with your manuscript and will be made available to the editor and reviewers.
>
> * Full and uncropped western blots, uploaded as 'Supplemental Material' & cited in the manuscript. Please note that, should
> your article be accepted for publication, the Supplementary Material that includes the original western blots will be
> published alongside the article.
>
> * Please do not include previous versions of your manuscript in your revised submission, by taking care to ensure the

[Quoted text hidden]

Nikolaos Patikas, PhD
Post-doctoral Research Fellow
Brigham and Women's Hospital
Harvard Medical School

The information in this e-mail is intended only for the person to whom it is addressed. If you believe this e-mail was sent to you in error and the e-mail contains patient information, please contact the Mass General Brigham Compliance HelpLine at https://www.massgeneralbrigham.org/complianceline <https://www.massgeneralbrigham.org/complianceline> .
Please note that this e-mail is not secure (encrypted). If you do not wish to continue communication over unencrypted e-mail, please notify the sender of this message immediately.
Continuing to send or respond to e-mail after receiving this message means you understand and accept this risk and wish to continue to communicate over unencrypted e-mail.

Will Plumbly <plumbwong@gmail.com> Mon, Dec 4, 2023 at 9:46 AM
To: Manos Metzakopian <manos.metzakopian@bit.bio>

Hi Manos,

I approve of the addtion of James Woodward to the manuscript.

many thanks,

Will Plumbly

On Sat, Dec 2, 2023 at 2:58 PM Manos Metzakopian <manos.metzakopian@bit.bio> wrote:

> Dear Authors,
>
> It is great pleasure to inform you that we are now ready to submit the manuscript revisions and reply to reviewers.
> In order to finalise the new data we have had an excellent contribution of experiment and analysis done by James Woodward (cced) with which we have increased the quality of the manuscript and he will be added as an author on the author list.
>
> **Please reply to this email confirming your approval to this change in authorship** as it is a requirement by the journal for progress of the manuscript in the review process and final acceptance if successful.
>
> See journal rule here: *"Requests to change the authorship (such as to include or exclude an author, change an author's name or contribution) must be accompanied by a letter signed by all authors to show they concur with the change. New authors must also confirm that they fully comply with the journal's authorship requirements."*
>
> Thank you for your quick reply and assistance! And congratulations once more on such great work on scientific progress of this manuscript.
>
> Best regards,
> Manos
>
> [Quoted text hidden]

Roy Chowdhury <src78@mrc-mbu.cam.ac.uk> Mon, Dec 4, 2023 at 1:43 PM
To: Manos Metzakopian <manos.metzakopian@bit.bio>

Hi Manos,

Congratulations and good luck with the re sub. I'm not sure if my approval is needed but I'm happy to give it anyway.

Cheers,

Roiy

On 02-12-2023 14:58, Manos Metzakopian wrote:

> Dear Authors,
>
> It is great pleasure to inform you that we are now ready to submit the manuscript revisions and reply to reviewers.
> In order to finalise the new data we have had an excellent contribution of experiment and analysis done by James Woodward (cced) with which we have increased the quality of the manuscript and he will be added as an author on the author list.
>
> **Please reply to this email confirming your approval to this change in authorship** as it is a requirement by the journal for progress of the manuscript in the review process and final acceptance if successful.
>
> See journal rule here: *"Requests to change the authorship (such as to include or exclude an author, change an author's name or contribution) must be accompanied by a letter signed by all authors to show they concur with the change. New authors must also confirm that they fully comply with the journal's authorship requirements."*
>
> Thank you for your quick reply and assistance! And congratulations once more on such great work on scientific progress of this manuscript.
>
> Best regards,
> Manos

>
> [Quoted text hidden]


```
--
Roy Chowdhury

Head of Light Microscopy Facility (MRC-MBU)
Postdoctoral Fellow (Mitochondrial Physiology Group)

MRC Mitochondrial Biology Unit
University of Cambridge
The Keith Peters Building
Cambridge Biomedical Campus
Hills Road
Cambridge CB2 0XY
```

Manos Metzakopian <manos.metzakopian@bit.bio> Mon, Dec 4, 2023 at 6:50 PM
To: Emma L Wilson <elwilson92@gmail.com>, Julien Prudent <julien.prudent@mrc-mbu.cam.ac.uk>, gl515@cam.ac.uk, jlm96@cam.ac.uk, m795@cam.ac.uk, njs76@cam.ac.uk, pra29@cam.ac.uk, sf26@sanger.ac, shyl2@cam.ac.uk, vjop2@mrc-mbu.cam.ac.uk
Cc: jaw209@cam.ac.uk

Hello everyone,

A gentle reminder for request below to provide your approval. We plan to
Submit on Thursday/Friday at the latest please.

People not on this email have already provided their approval. Thank you!

Exciting progress!

Best regards,
Manos
[Quoted text hidden]

Manos Metzakopian <manos.metzakopian@bit.bio> Tue, Dec 5, 2023 at 9:02 AM
To: sf26@sanger.ac.uk
Cc: Emma L Wilson <elwilson92@gmail.com>

Your reply is appreciated.

Thanks

Manos

[Quoted text hidden]

Jordan Morris <jlm96@cam.ac.uk> Tue, Dec 5, 2023 at 11:57 AM
To: Manos Metzakopian <manos.metzakopian@bit.bio>

Hello,

Yes I approve, thank you for your work!

Best
Jordan

Sent from Outlook for iOS

From: Manos Metzakopian <manos.metzakopian@bit.bio>
Sent: Monday, December 4, 2023 6:50:24 PM
To: Emma L Wilson <elwilson92@gmail.com>; Julien Prudent <julien.prudent@mrc-mbu.cam.ac.uk>; Georgina Elizabeth Lindop <gl515@cam.ac.uk>; Jordan Morris <jlm96@cam.ac.uk>; m795@cam.ac.uk <m795@cam.ac.uk>; Nuno Santos Leal <njs76@cam.ac.uk>; Philip Antrobus <pra29@cam.ac.uk>; sf26@sanger.ac <sf26@sanger.ac>; Sam Loh <shyl2@cam.ac.uk>; vjop2@mrc-mbu.cam.ac.uk <vjop2@mrc-mbu.cam.ac.uk>
Cc: James Woodward <jaw209@cam.ac.uk>
Subject: Re: URGENT: AUTHOR CHANGE APPROVAL REQUIRED::Re: Decision letter for CDDIS-23-2444-T

Hello everyone,

A gentle reminder for request below to provide your approval. We plan to
Submit on Thursday/Friday at the latest please.

People not on this email have already provided their approval. Thank you!

Exciting progress!

Best regards,
Manos

[Quoted text hidden]

Emma Wilson <elwilson92@gmail.com> Tue, Dec 5, 2023 at 8:44 AM
To: Manos Metzakopian <manos.metzakopian@bit.bio>

Dear Manos

I am very happy for James to be added to the paper CDDIS-23-2444-T.

Thanks

Emma

On Mon, 4 Dec 2023, 18:50 Manos Metzakopian, <manos.metzakopian@bit.bio> wrote:

> Hello everyone,
>
> A gentle reminder for request below to provide your approval. We plan to
> Submit on Thursday/Friday at the latest please.
>
> People not on this email have already provided their approval. Thank you!
>
> Exciting progress!
>
> Best regards,
> Manos
>
> [Quoted text hidden]

---

4 attachments

image755305.png
4K

image784815.png
3K

image669034.png
22K

image784815.png
3K

Manos Metzakopian <manos.metzakopian@bit.bio> Tue, Dec 5, 2023 at 7:16 PM
To: vjop2@mrc-mbu.cam.ac.uk, gl515@cam.ac.uk, pra29@cam.ac.uk, m795@cam.ac.uk, shyl2@cam.ac.uk
Cc: jaw209@cam.ac.uk, Julien Prudent <julien.prudent@mrc-mbu.cam.ac.uk>, Luis Miguel <lm850@cam.ac.uk>, Emma L Wilson <elwilson92@gmail.com>

Dear Authors,

Your reply would be appreciated to the email below. You remain to provide your approval to the author change.
Thank you for your attention.

Miguel, James and Emma are cced for visibility.

Best regards,
Manos

On Sat, Dec 2, 2023 at 2:58 PM Manos Metzakopian <manos.metzakopian@bit.bio> wrote:
[Quoted text hidden]
[Quoted text hidden]

Vincent Paupe <vjop2@mrc-mbu.cam.ac.uk> Tue, Dec 5, 2023 at 7:35 PM
To: Manos Metzakopian <manos.metzakopian@bit.bio>

Hello

I approve the change of authorship.
Good luck!

Best wishes

Vincent Paupe

> On 5 Dec 2023, at 19:16, Manos Metzakopian <manos.metzakopian@bit.bio> wrote:
>
> Dear Authors,
>
> Your reply would be appreciated to the email below. You remain to provide your approval to the author change.
> Thank you for your attention.
>
> Miguel, James and Emma are cced for visibility.
>
> Best regards,
> Manos
>
> **Manos Metzakopian**
> VP, Research and Development
>
> T +44 (0)1223 787297
> manos.metzakopian@bit.bio
>
> The Dorothy Hodgkin Building
> Babraham Research Campus
> Cambridge CB22 3FH
> United Kingdom
>
> info@bit.bio | www.bit.bio
> Follow us <image634222.png> <image547740.png>
>
> <image591749.png>
>
> Notice: This message is the property of Bit Bio Ltd and contains information that may be confidential and/or privileged. If you are not the intended recipient, you should not use, disclose or take any action based on this message. If you have received this transmission in error, please immediately contact the sender by return e-mail and delete this e-mail, and any attachments, from any computer.
>
> [Quoted text hidden]
> [Quoted text hidden]
> [Quoted text hidden]

Sam Loh <shyl2@mrc-tox.cam.ac.uk> Wed, Dec 6, 2023 at 8[illegible]
To: Manos Metzakopian <manos.metzakopian@bit.bio>

Dear Manos,
I am writing to confirm my approval to the change in authorship.
Thank you.
Best regards,
Sam

Dr. Samantha Loh
MRC Toxicology Unit
University of Cambridge
Gleeson Building, Tennis Court Road
Cambridge CB2 1QR
shyl2@cam.ac.uk

On 4 Dec 2023, at 18:50, Manos Metzakopian <manos.metzakopian@bit.bio> wrote:

Hello everyone,

A gentle reminder for request below to provide your approval. We plan to
Submit on Thursday/Friday at the latest please.

People not on this email have already provided their approval. Thank you!

Exciting progress!

Best regards,
Manos

Manos Metzakopian
VP, Research and Development

T +44 (0)1223 787297
manos.metzakopian@bit.bio

The Dorothy Hodgkin Building
Babraham Research Campus
Cambridge CB22 3FH
United Kingdom

info@bit.bio | www.bit.bio
Follow us <image686102.png> <image864965.png>

<image094539.png>

Notice: This message is the property of Bit Bio Ltd and contains information that may be confidential and/or privileged. If you are not the intended recipient, you should not use, disclose or take any action based on this message. If you have received this transmission in error, please immediately contact the sender by return e-mail and delete this e-mail, and any attachments, from any computer.

[Quoted text hidden]
[Quoted text hidden]

Nuno Leal <nunojsleal@gmail.com> Wed, Dec 6, 2023 at 9:16 AM
To: Manos Metzakopian <manos.metzakopian@bit.bio>

Hi Manos,

I confirm my approval of adding James Woodward to this publication.

Nuno

On 2 Dec 2023, at 15:01, Manos Metzakopian <manos.metzakopian@bit.bio> wrote:

Dear Authors,

It is great pleasure to inform you that we are now ready to submit the manuscript revisions and reply to reviewers.
In order to finalise the new data we have had an excellent contribution of experiment and analysis done by James Woodward (cced) with which we have increased the quality of the manuscript and he will be added as an author on the author list.

Please reply to this email confirming your approval to this change in authorship as it is a requirement by the journal for progress of the manuscript in the review process and final acceptance if successful.

See journal rule here: *"Requests to change the authorship (such as to include or exclude an author, change an author's name or contribution) must be accompanied by a letter signed by all authors to show they concur with the change. New authors must also confirm that they fully comply with the journal's authorship requirements."*

Thank you for your quick reply and assistance! And congratulations once more on such great work on scientific progress of this manuscript.

Best regards,
Manos

Manos Metzakopian
VP, Research and Development

T +44 (0)1223 787297
manos.metzakopian@bit.bio

The Dorothy Hodgkin Building
Babraham Research Campus
Cambridge CB22 3FH
United Kingdom

info@bit.bio | www.bit.bio
Follow us <image229224.png> <image543071.png>

<image424821.png>

Notice: This message is the property of Bit Bio Ltd and contains information that may be confidential and/or privileged. If you are not the intended recipient, you should not use, disclose or take any action based on this message. If you have received this transmission in error, please immediately contact the sender by return e-mail and delete this e-mail, and any attachments, from any computer.

[Quoted text hidden]
[Quoted text hidden]
[Quoted text hidden]
[Quoted text hidden]

Manos Metzakopian <manos.metzakopian@bit.bio> Wed, Dec 6, 2023 at 10:30 AM
To: Julien Prudent <julien.prudent@mrc-mbu.cam.ac.uk>, Luis Miguel <lm850@cam.ac.uk>

Hi Julien and Miguel,

Not sure it is needed since you are corresponding but just in case could you give me a response to the email below.

Thanks!

Manos

On Sat, 2 Dec 2023 at 14:58, Manos Metzakopian <manos.metzakopian@bit.bio> wrote:
[Quoted text hidden]

Emma Wilson <elwilson92@gmail.com> Thu, Dec 7, 2023 at 9:27 AM
To: Julien Prudent <jmpp2@mrc-mbu.cam.ac.uk>, Luis Miguel Martins <lm795@mrc-tox.cam.ac.uk>, Manos Metzakopian <manos.metzakopian@bit.bio>, Georgina Elizabeth Lindop <gl515@cam.ac.uk>, Philip Antrobus <pra29@cam.ac.uk>

Hi Everyone

Can you please see Manos's email below and please reply to him or me with your confirmation for authorship changes. We will be submitting today.

Thanks

Emma

---------- Forwarded message ---------
From: **Manos Metzakopian** <manos.metzakopian@bit.bio>
Date: Sat, Dec 2, 2023 at 2:58 PM
Subject: URGENT: AUTHOR CHANGE APPROVAL REQUIRED::Re: Decision letter for CDDIS-23-2444-T
To: Emma L Wilson <elwilson92@gmail.com>, <yizhou0421@gmail.com>, <sf26@sanger.ac>, <plumbwong@gmail.com>, <jlm96@cam.ac.uk>, <vjop2@mrc-mbu.cam.ac.uk>, <njs76@cam.ac.uk>, <src78@mrc-mbu.cam.ac.uk>, <gl515@cam.ac.uk>, <pra29@cam.ac.uk>, Julien Prudent <julien.prudent@mrc-mbu.cam.ac.uk>, <m795@cam.ac.uk>, <shyl2@cam.ac.uk>, <npatikas@bwh.harvard.edu>, <yma24@cam.ac.uk>
Cc: <jaw209@cam.ac.uk>

Dear Authors,

It is great pleasure to inform you that we are now ready to submit the manuscript revisions and reply to reviewers.
In order to finalise the new data we have had an excellent contribution of experiment and analysis done by James Woodward (cced) with which we have increased the quality of the manuscript and he will be added as an author on the author list.

Please reply to this email confirming your approval to this change in authorship as it is a requirement by the journal for progress of the manuscript in the review process and final acceptance if successful.

See journal rule here: *"Requests to change the authorship (such as to include or exclude an author, change an author's name or contribution) must be accompanied by a letter signed by all authors to show they concur with the change. New authors must also confirm that they fully comply with the journal's authorship requirements."*

Thank you for your quick reply and assistance! And congratulations once more on such great work on scientific progress of this manuscript.

Best regards,
Manos

[Quoted text hidden]

Philip Antrobus <pra29@cam.ac.uk> Thu, Dec 7, 2023 at 10:51 AM
To: Emma Wilson <elwilson92@gmail.com>, Julien Prudent <jmpp2@mrc-mbu.cam.ac.uk>, Luis Martins <lm795@mrc-tox.cam.ac.uk>, Manos Metzakopian <manos.metzakopian@bit.bio>, Georgina Elizabeth Lindop <gl515@cam.ac.uk>

Apologies for the delay, the email was directed to my junk folder. I'm happy with the pro[posed changes.

Regards,

Robin

From: Emma Wilson <elwilson92@gmail.com>
Sent: Thursday, December 7, 2023 9:27 AM
To: Julien Prudent <jmpp2@mrc-mbu.cam.ac.uk>; Luis Martins <lm795@mrc-tox.cam.ac.uk>; Manos Metzakopian <manos.metzakopian@bit.bio>; Georgina Elizabeth Lindop <gl515@cam.ac.uk>; Philip Antrobus <pra29@cam.ac.uk>
Subject: Fwd: URGENT: AUTHOR CHANGE APPROVAL REQUIRED::Re: Decision letter for CDDIS-23-2444-T

[Quoted text hidden]

Georgina Elizabeth Lindop <gl515@cam.ac.uk> Thu, Dec 7, 2023 at 11:03 AM
To: Manos Metzakopian <manos.metzakopian@bit.bio>
Cc: Emma Wilson <elwilson92@gmail.com>

Hi Manos,
I also give approval to the changes.
Sorry for the delay as this also went to my junk folder
Best wishes,
Georgina

Sent from Outlook for Android

From: Manos Metzakopian <manos.metzakopian@bit.bio>
Sent: Saturday, December 2, 2023 2:58:00 PM
To: Emma L Wilson <elwilson92@gmail.com>; yizhou0421@gmail.com <yizhou0421@gmail.com>; sf26@sanger.ac <sf26@sanger.ac>; plumbwong@gmail.com <plumbwong@gmail.com>; Jordan Morris <jlm96@cam.ac.uk>; vjop2@mrc-mbu.cam.ac.uk <vjop2@mrc-mbu.cam.ac.uk>; Nuno Santos Leal <njs76@cam.ac.uk>; src78@mrc-mbu.cam.ac.uk <src78@mrc-mbu.cam.ac.uk>; Georgina Elizabeth Lindop <gl515@cam.ac.uk>; Philip Antrobus <pra29@cam.ac.uk>; Julien Prudent <julien.prudent@mrc-mbu.cam.ac.uk>; m795@cam.ac.uk <m795@cam.ac.uk>; Sam Loh <shyl2@cam.ac.uk>; npatikas@bwh.harvard.edu <npatikas@bwh.harvard.edu>; Yusuf Adia <yma24@cam.ac.uk>
Cc: James Woodward <jaw209@cam.ac.uk>
Subject: URGENT: AUTHOR CHANGE APPROVAL REQUIRED::Re: Decision letter for CDDIS-23-2444-T

Dear Authors,

It is great pleasure to inform you that we are now ready to submit the manuscript revisions and reply to reviewers.

bit.bio
THE CELL CODING COMPANY

Manos Metzakopian <manos.metzakopian@bit.bio>

Confirm adding James Woodward as author

3 messages

Manos Metzakopian <manos.metzakopian@bit.bio> Thu, Dec 7, 2023 at 8:32 AM
To: Luis Martins <lm795@cam.ac.uk>
Cc: Julien Prudent <jmpp2@mrc-mbu.cam.ac.uk>, Luis Martins <lm795@mrc-tox.cam.ac.uk>, Luis Martins <lm795@cam.ac.uk>

Hi Julien and Miguel,

Could you reply confirming you approve adding James as an author to the manuscript? Unless you think corresponding authors do not need to confirm.

Thank you!

Manos

Luis Martins <lm795@cam.ac.uk> Thu, Dec 7, 2023 at 9:23 AM
To: Manos Metzakopian <manos.metzakopian@bit.bio>
Cc: Julien Prudent <jmpp2@mrc-mbu.cam.ac.uk>

Dear Manos,

I agree with adding James Woodward as a co-author in our manuscript entitled "Genome-wide CRISPR/Cas9 screen shows that loss of GET4 increases mitochondria-endoplasmic reticulum contact sites and is neuroprotective"

All the best,

Miguel

Dr. L. M. Martins, MRC Investigator
MRC Toxicology Unit, University of Cambridge
Gleeson Building
Tennis Court Road
Cambridge CB2 1QR
United Kingdom
https://www.mrc-tox.cam.ac.uk/research/l-m-martins-programme
Authored Publications (Google Scholar):
https://scholar.google.com/citations?user=-1xTiLYAAAAJ&hl=en&oi=ao

[Quoted text hidden]
[Quoted text hidden]

Manos Metzakopian
VP, Research and Development

T +44 (0)1223 787297
manos.metzakopian@bit.bio

The Dorothy Hodgkin Building
Babraham Research Campus
Cambridge CB22 3FH
United Kingdom

info@bit.bio | www.bit.bio
Follow us <image957414.png> <image457662.png>

<image688809.png>

Notice: This message is the property of Bit Bio Ltd and contains information that may be confidential and/or privileged. If you are not the intended recipient, you should not use, disclose or take any action based on this message. If you have received this transmission in error, please immediately contact the sender by return e-mail and delete this e-mail, and any attachments, from any computer.

Julien Prudent <jmpp2@mrc-mbu.cam.ac.uk> Thu, Dec 7, 2023 at 10:18 AM
To: Luis Martins <lm795@cam.ac.uk>
Cc: Manos Metzakopian <manos.metzakopian@bit.bio>

Dear Manos.

I confirm that I agreed to add James Woodward as co-author in our study "Genome wide CRISPR/Cas9 screen shows that loss of GET4 increases mitochondria-ebdoplasmic reticulum contact sites and is neuroprotective".

Best wishes,
Julien

- -

Dr. Julien Prudent
MRC Investigator and Mitochondrial Cell Biology Programme Leader
Medical Research Council Mitochondrial Biology Unit
The Keith Peters Building
University of Cambridge
Cambridge Biomedical Campus
Hills Road
Cambridge
CB2 0XY
julien.prudent@mrc-mbu.cam.ac.uk
https://www.mrc-mbu.cam.ac.uk/
+44 (0)1223 252770

UKRI MRC Mitochondrial Biology Unit UNIVERSITY OF CAMBRIDGE

[Quoted text hidden]

bit.bio
THE CELL CODING COMPANY

Manos Metzakopian <manos.metzakopian@bit.bio>

Re: URGENT: AUTHOR CHANGE APPROVAL REQUIRED::Re: Decision letter for CDDIS-23-2444-T [EXT]

2 messages

Sarah Frances Field <sf26@sanger.ac.uk> Tue, Dec 5, 2023 at 9:34 AM
To: Manos Metzakopian <manos.metzakopian@bit.bio>
Cc: Emma L Wilson <elwilson92@gmail.com>

Hi Manos,

I absolutely approve the change in authorship to include James Woodward.

Do I need to send this reply to the publishers as well, or just to you?

All the best, and congratulations Emma!

Sally

From: Manos Metzakopian <manos.metzakopian@bit.bio>
Date: Tuesday, 5 December 2023 at 09:02
To: Sarah Frances Field <sf26@sanger.ac.uk>
Cc: Emma L Wilson <elwilson92@gmail.com>
Subject: Fwd: URGENT: AUTHOR CHANGE APPROVAL REQUIRED::Re: Decision letter for CDDIS-23-2444-T [EXT]

Your reply is appreciated.

Thanks

Manos

Manos Metzakopian
VP, Research and Development

T +44 (0)1223 787297

manos.metzakopian@bit.bio

The Dorothy Hodgkin Building
